# Circulating VEGF-A Levels in Relation to Retinopathy of Prematurity and Treatment Effects: A Systematic Review and Meta-Analysis

**DOI:** 10.1016/j.xops.2024.100548

**Published:** 2024-05-07

**Authors:** Ulrika Sjöbom, Tove Hellqvist, Jhangir Humayun, Anders K. Nilsson, Hanna Gyllensten, Ann Hellström, Chatarina Löfqvist

**Affiliations:** 1 Learning and Leadership for Health Care Professionals At the Institute of Health and Care Science at Sahlgrenska Academy at University of Gothenburg, Gothenburg, Sweden; 2 Department of Clinical Neuroscience At the Institution of Neuroscience and Physiology at Sahlgrenska Academy at University of Gothenburg, Gothenburg, Sweden

**Keywords:** Vascular-endothelial growth factor, ROP treatment, VEGF concentrations, Biomarker, Systemic treatment effect

## Abstract

**Topic:**

Retinopathy of prematurity (ROP) is a severe retinal vascular disorder affecting preterm infants, potentially leading to retinal detachment and blindness. This review aims to elucidate the relationship between systemic VEGF levels and ROP.

**Clinical Relevance:**

This systematic review aims to consolidate evidence from available studies to guide future research and inform clinical practice. In particular, the role of circulating VEGF-A levels in predicting ROP onset and progression, and evaluating the impact of anti-VEGF therapy on these levels, is crucial in ensuring efficacy and safety in patient care.

**Methods:**

Scopus and PubMed were searched to identify studies investigating circulating VEGF-gene products in ROP patients using immunologic assays. Two authors independently screened the literature and extracted data, employing a random-effects meta-analysis to compare VEGF levels as the ratio of means between ROP patients and controls before and after treatment, heterogeneity was reported by I^2^-statistics. Risks of bias and publication bias were assessed using Quality Assessment of Diagnostic Accuracy Studies-2 and funnel plots/Egger’s tests, respectively.

**Results:**

Out of 941 papers, 54 were included, with 26 providing posttreatment data and 31 providing biomarker data. Findings show a significant decrease in VEGF-A levels in the first week after ROP treatment (ratio of means [95% confidence interval] 0.34 [0.25–0.45], I^2^ = 97%, 17 publications). Anti-VEGF therapy showed a significantly more pronounced decrease (0.31 [0.25–0.38], I^2^ = 40%, 7 publications) than laser treatment in the first week after treatment (0.77 [0.61–0.97], I^2^ = 42%, 2 publications, subgroup difference, *P* < 0.01), among studies with a low risk of bias. Serum samples demonstrated a more marked decrease in VEGF-A than plasma (subgroup difference *P* < 0.01). However, the use of blood VEGF-A concentration as a biomarker for ROP prediction has shown inconsistent trends. The risk of bias mainly stems from unclear patient selection and lack of sample timing or analytical method details.

**Conclusion:**

While anti-VEGF treatment significantly reduced blood VEGF-A levels in the first week post-ROP treatment, blood VEGF-A levels did not consistently predict ROP development. Heterogeneity in the results underscores the need for optimized analytical methods and emphasizes the importance of considering individual variation in VEGF-A concentrations independent of ROP diagnosis.

**Financial Disclosure(s):**

The author(s) have no proprietary or commercial interest in any materials discussed in this article.

Retinopathy of prematurity (ROP), a retinal neuro-vascular disorder primarily observed in preterm infants, stands as one of the leading causes of preventable childhood blindness globally.^1^ Characterized by aberrant neovascularization within the avascular regions of the retina, ROP can lead to detrimental outcomes such as retinal detachment and blindness.^2^

The complex pathophysiology of ROP involves abnormal development of retinal blood vessels.^3^ VEGF plays a key role in this process and is vital for retinal angiogenesis. The first phase of ROP is induced by hyperoxia exposure followed by decreased VEGF levels, and results in attenuation and cessation of retinal vessel growth. The second phase is characterized by abnormal retinal vessel growth due to increased VEGF concentrations following retinal hypoxia and increasing insulin growth factor 1 levels.^3^ Interestingly, the association between systemic circulating VEGF levels and ROP severity in infants is inconsistent,^4^ adding complexity to our understanding of the precise role of VEGF in ROP pathogenesis.

Retinopathy of prematurity management strategies encompass anti-VEGF therapy and laser photocoagulation.^5^ However, diverse methodological approaches in conducted studies and heterogeneity in patient cohorts have led to inconsistent findings regarding the efficacy of these treatments and their effect on systemic VEGF levels.^6^ Notably, a Cochrane review underscored the potential benefits of anti-VEGF therapy, such as simplicity of administration and cost-effectiveness, but simultaneously raised safety concerns regarding its long-term use in the pediatric population.^7^ Systematic reviews have been performed regarding anti-VEGF efficiency, refractive outcomes, ocular safety, recurrence,^8^, ^9^, ^10^, ^11^, ^12^, ^13^ adverse events,^10^^,^^14^ and neurodevelopmental outcomes.^15^ A systematic review investigating VEGF levels in relation to ROP development and ROP treatment with laser and bevacizumab (an anti-VEGF drug) was conducted in 2017, which included 12 publications but did not include a meta-analysis.^4^

By compiling and synthesizing the available evidence on VEGF levels, the extent of variability and its potential sources can be discerned. By illuminating key methodologic differences across studies investigating infants diagnosed with ROP and treatment cohorts, we built evidence based on the most rigorous and reliable studies that will benefit practicing ophthalmologists, researchers, and, ultimately, the patients affected by this potentially blinding condition. This review will improve our understanding of the role of VEGF in ROP, and pave the way for the development of effective and safe treatment strategies.

Considering the scarcity of systematic reviews that concentrate on comparing VEGF levels in preterm infants with respect to ROP occurrence and severity, including different treatment regimens, there is a need for an updated review on the topic. Our approach was comprehensive and quantitative, systematically scouring major databases for relevant studies, critically evaluating their quality, and extracting and analyzing data, all in line with the Preferred Reporting Items for Systematic Reviews and Meta-Analyses guidelines.^16^ This review summarizes the reported levels of VEGF in blood samples associated with ROP. The secondary aim was to evaluate whether ROP treatment in premature infants affects circulating VEGF levels. Finally, we aimed to summarize and describe systemic VEGF concentrations determined using different blood sampling systems.

## Methods

The protocol for this systematic review was registered in August 2021 at Prospero (CRD42021265914). The study reports on published and available data and does not require ethical approval.

### Eligibility Criteria for Considering Studies for This Review

The eligibility criteria for including studies in this review encompassed all types of studies that investigated concentrations of any VEGF-gene product in blood using an immunologic assay, irrespective of isoform specificity, in connection to ROP. Publications lacking reports on VEGF concentrations or those devoid of VEGF concentrations related to ROP were excluded. Studies were grouped based on if they reported VEGF concentration as a biomarker for ROP or if they reported VEGF concentrations in relation to ROP treatment.

### Search Methods for Identifying Studies

Scopus and PubMed were initially searched on August 13, 2021, and updated on May 15, 2023 (no additional publications were identified) without restrictions on language, publication status, or date of publication. This search strategy is based on 2 blocks of terms derived from 2 completed and related reviews, one focusing on ROP and the other on VEGF. An additional block aimed to capture the analytical measurement, and this was reviewed by a librarian with expertise in search strategies for systematic reviews at the University of Gothenburg; search strategies are presented in Supplement Appendix 1. Additionally, the citations and references of the included publications were manually searched for relevant publications on November 10, 2022. Google Scholar was searched on May 9, 2023, using the terms (ROP, VEGF, serum, and plasma), and the first 100 hits were assessed to include possible gray literature.

### Study Selection

The inclusion/exclusion process in Rayyan QCRI, was conducted independently by 2 researchers (U. S. and J. H., or T. H.), with conflicts resolved by a third (C. L.).

### Data Collection and Risk of Bias Assessment

Data extraction from each publication was performed independently in duplicate by 2 researches (U. S. and C. L. or J. H., or T. H.). An Excel extraction sheet was utilized for data collection, which included an assessment of the risk of bias following the Quality Assessment of Diagnostic Accuracy Studies-2 guidelines.^17^^,^^18^ Quality Assessment of Diagnostic Accuracy Studies-2 has been the standard for evaluating the risk of bias in diagnostic accuracy studies. The risk of bias assessment was presented per included outcome (ROP treatment and biomarker of ROP) and weighted by the relative effective sample size, as recommended by Deeks et al,^19^ where effective sample size =(4n1n2)/(n1+n2). In cases where multiple reports were generated from the same study, the average number (n) for each group was employed. Evaluation of bias considered 4 domains: *patient selection, index test, reference standard,* and *flow and timing*, with particular attention to parameters influencing the interpretation and measurement of VEGF levels. Visualization of results was accomplished using the web-based app robvis.^20^

### Data Synthesis and Analysis

VEGF concentrations were extracted for groups with ROP and corresponding controls, both before and after treatment, along with the reported time points for measurement. Concentrations were converted to pg/mL if they were reported in an alternative unit, and if no unit was reported, the standard unit from the analytical kit was assumed. Results from statistical comparisons in the publications included important demographic information about the infants or groups of infants, as well as the definition of ROP and/or control. This data were tabulated together with general study information, ie, time and place for study and study design. The extraction protocol is presented in Supplement Appendix 2. If VEGF concentrations were solely depicted in figures, GetData Graph Digitizer, version 2.26.0.20, was employed for extraction.

Meta-analysis was conducted using the meta-package^21^ in RStudio, version 1.2.5033, R, version 4.0.4 (R Foundation for Statistical Computing).^22^ Means and standard deviations (SDs) were, when appropriate, approximated from available sample sizes, medians, ranges, and/or interquartile ranges, following the methods proposed by Luo et al^23^ for means and those for SDs proposed by Wan et al^24^ and Shi et al.^25^ If necessary, groups were summarized using the meta.mean function, and the resulting confidence intervals (CIs) were utilized to calculate SDs according to SD = √Nx(upper limit–lower limit)/3.92.

Groups were compared using the meta.cont function to calculate the ratio of means (ROM), facilitating the comparison of results measured using different analytical methods and scales.^26^ Meta-analysis was conducted using a random-model and inverse variance methods for pooling. Ratio of means was employed for result reporting, accompanied by a 95% CI, to enhance interpretation. The standardized mean difference was calculated as a sensitivity method to investigate whether the outcome was consistent to ROM concerning heterogeneity and subgroup differences. Mean concentrations for each group (ROP treatment and biomarker of ROP) are reported together with the SDs in Supplement Appendix 3.

VEGF levels were illustrated longitudinally after treatment both as reported mean concentrations together with 95% CI and as ROM. For VEGF as a biomarker for ROP, the ROM of VEGF concentrations for the ROP group compared with the control gorup were illustrated longitudinally. Figures were created with the R ggplot-package.

**Figure 1:**
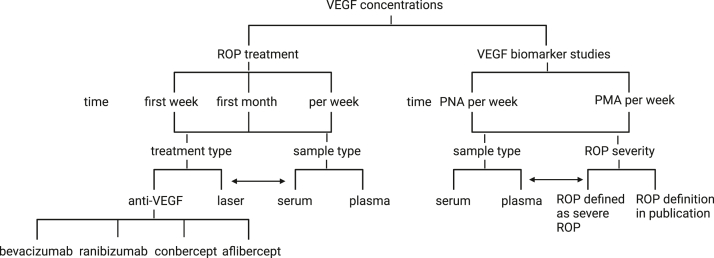
Strategy for subgroup analysis to explore heterogeneity in meta-analysis. Subgroups were investigated for the treatment type and sample type for ROP treatment based on the time after treatment. For VEGF as a biomarker for ROP, subgroups were investigated based on the sample type and severity of ROP per time for VEGF measurement. PMA = postmenstrual age; PNA = postnatal age; ROP = retinopathy of prematurity.

Heterogeneity was investigated by using the chi-square test and reported as I^2^ statistics, and this was further evaluated in subgroup analysis illustrated in Figure 1.

Heterogeneity is categorized into the following groups.^27^

• 0% to 40%: might not be important
• 30% to 60%: may represent moderate heterogeneity
• 50% to 90%: may represent substantial heterogeneity
• 75% to 100%: considerable heterogeneity

Additionally, observed outliers were individually excluded to identify potential impacts on the results. Meta-analyses were then conducted with and without studies estimated to have a high risk of bias in one or more domains. If no differences were observed in the interpretation of the results, they were presented for all included studies. Publication bias was evaluated by examining funnel-plot asymmetry, plotting the ROM against standard error and 1/√effective sample size, along with Eggers test using the standard definition.

*P* values and the direction of differences were summarized according to synthesis without meta-analysis in systematic reviews reporting guidelines^28^ as a sensitivity analysis to control the direction achieved in the meta-analysis.

Forest and funnel plots supporting these results are presented in Supplemental Appendix 3 (Supplemental figures).

### Quality of Evidence

The quality of evidence for the reported findings was assessed using the Grading of Recommendations, Assessment, Development, and Evaluation tool to determine the certainty of these findings. Grading of Recommendations, Assessment, Development, and Evaluation is a recognized tool for evaluating the best available evidence in the development of health care recommendations.^29^ The assessment considered various factors, including the risk of bias, inconsistency, indirectness, imprecision, risk of publication bias, magnitude of the effect*,* plausible confounding factors, and the presence of a dose-response gradient.

## Results

### Literature Search

**Figure 2:**
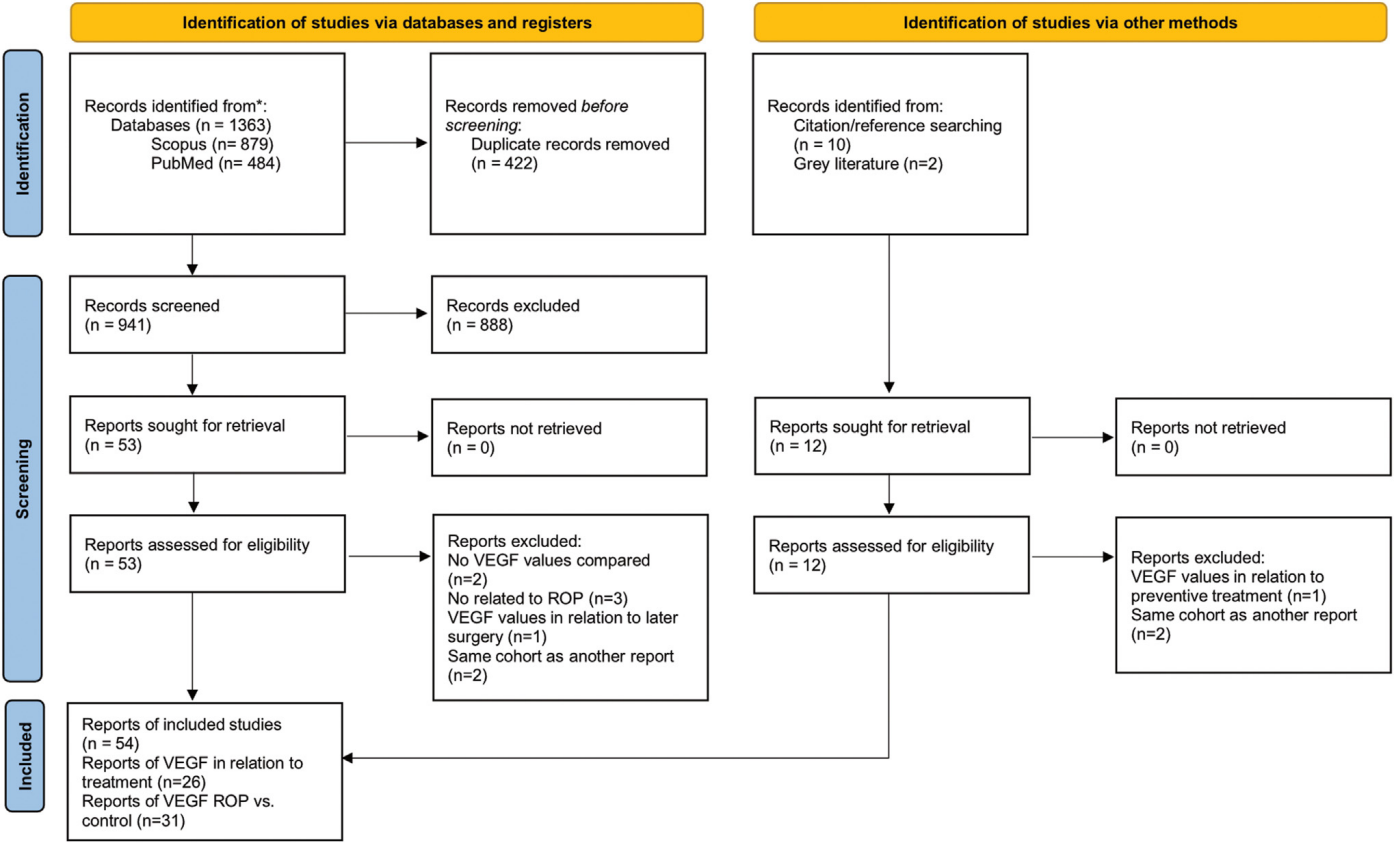
PRISMA flowchart over the inclusion/exclusion process. PRISMA = Preferred Reporting Items for Systematic Reviews and Meta-Analyses; ROP = retinopathy of prematurity.

The inclusion and exclusion processes are detailed the flowchart (Fig 2). A total of 54 publications were included in the study: 45 were identified through the search strategy in databases and 9 from alternative sources. Two was found by searching gray literature, 1 of those originated from a conference abstract, and 10 were discovered through exploration of citations and references in the included publications. Details regarding the subsequently excluded publications are reported in the Supplement Appendix 3, Table S1 (available at www.ophthalmologyscience.org). Among the diverse VEGF isoforms, VEGF-A emerged as the most extensively examined, with levels reported in all included studies. Consequently, our meta-analysis was centered on the assessment of VEGF-A levels.

### VEGF Levels after ROP Treatment

A total of 26 studies investigating VEGF-A levels before and after ROP treatment were identified^30^, ^31^, ^32^, ^33^, ^34^, ^35^, ^36^, ^37^, ^38^, ^39^, ^40^, ^41^, ^42^, ^43^, ^44^, ^45^, ^46^, ^47^, ^48^, ^49^, ^50^, ^51^, ^52^, ^53^, ^54^, ^55^ (Table 2). Two studies were excluded from the meta-analysis, 1 due to the absence of specified sampling time points,^53^ and the other involving a combination of 2 treatments.^52^

**Table 2:** Included Publications Evaluating VEGF Levels Before and After Treatment of ROP

Study	Country	Study Design	Time for Study	Treatment	Sample System	Number Treated	Time-points before Treatment	Time-points after Treatment	Reason for Treatment	Statistical Method	Direction of Difference in VEGF From Baseline
Berrington et al. (2012) (abstract)	UK	C	2011–2012	IVB and laser	Plasma	laser 5, IVB 2	1–4 d	1–33 d	ROP stage 3	NA	NA
Cakir et al. (2019)	Turkey	P O C	February 2017–August 2018	Laser	Serum	73	1 d	4 d	Threshold ROP (CRYO-ROP), prethreshold ROP (ETROP) ICROP	Friedman and Wilcoxon test, *P* < 0.05	↓4 days after treatment,
Chen et al. (2019)	China	P O C	June 2017–July 2018	IVR (0.25 mg)	Serum	15	1 d	1, 3, and 7 d	Type 1 ROP was defined as stage 1 or 2 ROP in zone I with plus disease or stage 3 ROP in zone I, or stage 2 or 3 ROP with plus disease in zone II, ETROP/ICROP	Kruskal–Wallis test.	↓ d 1, 3, and 7
Cheng et al. (2020)	China	Consecutive P O C	November 2017–September 2018	IVC	Serum	60	--	1 and 4 w	Zone I/II Stage 2/3 ROP with plus disease or AP-ROP ICROP	Wilcoxon test, *P* < 0.05	↓ VEGF-A and D at 1 w, ND at 4 weeks
Cheng et al. (2022)	China	P multicenter nonrandomized clinical trial	January 2020–September 2020	IVC (0.25 mg)	Serum	40	--	1 and 4 w	Aggressive posterior ROP (APROP) or type 1 ROP ICROP	One-way repeated measures analysis of variance	↓ 1 w ND 4 w
Furuncuoglu et al. (2022)	Turkey	P O C	November 2018- July 2019	IVA (1 mg)	Serum	15	--	1 d, 1, 2, 4, and 8 w	Type 1 ROP in zone I and posterior zone II ETROP/ICROP	Friedman or Wilcoxon test, multiple imputation for missing	↓ at 1 d and 1, 2, 4, and 8 w
Hartnett et al. (2022)	US	phase 1, P dose de-escalation	May 2015–May 2019	IVB (0.002–0.875 mg)	Plasma	83	--	2 and 4 w	type 1 ROP ICROP	Tabulated but not tested	NA ↓ by 50% or more in 40 of 66 infants at w 2 and 31 of 61 infants at w 4. The proportions of infants with an increase in plasma VEGF-A levels, or a reduction in plasma VEGF-A levels by 1% to <25%, 25% to <50%, or 50% or more from preinjection levels
Hoerster et al. (2013)	Germany	CS	--	IVR (0.2 mg)	Serum	1	--	1, 3, and 4 w	ROP stage 3 in all quadrants in zones 1–2 with plus disease in both eyes	Not statistically tested	NA levels below the detection limit for 2 weeks
Hong et al. (2015)	South Korea	P O C	--	IVB (0.6 mg)	Plasma	6	--	1, 2, 3, 4, 5, 6, 7, and 8 w	Classified as having threshold ROP with a plus disease in Zone 1 or Zone 2 ICROP	Wilcoxon test or Mann–Whitney U-test	↓ from 1 to 7 w
Huang et al. (2018)	Taiwan	P O C	September 2014–August 2016	IVA (1 mg) and IVB (0.625 mg)	Serum	IVB: 9, IVA:5	1–2 d	2, 4, 8, and 12 w	Type 1 ROP ETROP/ICROP	Wilcoxon test and trends with Friedmans test	↓ for 12 w after treatment with IVA and IVB both in serum as well as the ratio of VEGF-A/platelet
Kong et al. (2015)	US	RCT	July 2012–January 2014	IVB (0.625 or 0.25 mg) laser	Serum	0.625 mg: 7, 0.25 mg: 10	--	2, 14, 42, and 60 d	Type 1 ROP ETROP/ICROP	Unclear statistical methods	↓ in all groups 2 d after treatment, more significant reduction in IVB-treated groups.
Kong et al. (2016)	US	P O C	--	IVB	Plasma	13	--	6 w	Type 1 ROP AAOP and APOS/ICROP	Unclear statistical methods	↓ VEGF-A and D 6 w after ↑ VEGF-C 6 w after
Imamoglu et al. (2014)	Turkey	P O C	November 2012–October 2013	Laser	Plasma	30	--	1 and 7 d	Prethreshold type 1 ROP was defined as follows: (1) any ROP in zone I with plus disease, or (2) zone I, stage 3 without plus disease, or (3) zone II, stage 2 or 3 with plus disease ICROP	Friedman test	↓ progressive decrease 1 d and 1 w after laser treatment
Iwahashi et al. (2021)	Japan	R CS	January 2012–February 2018	IVR (0.25 mg)	Serum	18	--	1 d, 1, 2, and 4 w	Type 1 or worse ICROP	Student t-test or the Wilcoxon test	↓1 and 7 d after--at 14 d after
Jiang et al. (2021)	China	R CS	January- July 2019	IVC (0.15, 0.20 or 0.25 mg)	Serum	23	--	1 d and 1 w	Type 1 prethreshold ROP ICROP	Mann–Whitney U-test	↓ first and seventh d after
Machalińska et al. (2013)	Poland	P O C	--	Laser	Plasma	29	PN age 10.1 (SD 3.5) w	PN age 15.9 (SD 6.1) w	ROP grade 3 or higher ICROP	Calculated by Wilcoxon test	ND
Saito et al. (2011)	Japan	CS	--	Laser and IVB in combination	Serum	1	--	--	ROP stage 3 zone 2	NA	NA
Sato et al. (2012)	Japan	CS	--	IVB (0.25/0.5 mg)	Serum	5	--	1 d, 1, and 2 w	Highly vascularative ROP ICROP	One-way repeated measures ANOVA followed by Holm-Sidak or Friedman repeated-measures followed by Dunn’s method.	↓ at 1 w after
Sedaghat et al. (2020)	Iran	P I CS	July 2016–December 2017	IVB (0.625 mg)	Plasma	10	--	1 and 2 m	ROP type 1, stage 3+ ROP in zone I or zone II posterior in both eyes	Repeated measures ANOVA with a Greenhouse–Geisser correction	↓ at month 1 and remained lower than pretreatment levels after 2 months
Stahl et al. (2018)	Germany	RCT multicenter, double-masked, investigator-initiated	September 2014–August 2016	IVR (0,12 and 0.2 mg)	Plasma	0.12 mg: 10, 0.2 mg: 9	--	1, 2, 3, 4, 5, 6 w	Patients with bilateral ROP in zone I (stages 1 with plus disease, 2 with plus disease, 3 with or without plus disease and aggressive posterior ROP) or posterior zone II (stage 3 with plus disease or aggressive posterior ROP) were eligible. ICROP	No statistical test reported	NA No sustained suppression of mean VEGF-A
Stahl et al. (2019)	26 countries	RCT open-label, superiority trial	December 2015–June 2017	IVR 0.1 mg, 0.2 mg and laser	Plasma	0.1 mg: 77, 0.2 mg: 74, laser: 74	Within 24 h	15 and 29 d, sparse sampling	Diagnosis of bilateral ROP zone I stage 1+, 2+, 3, or 3+, or zone II stage 3+, or aggressive posterior ROP ICROP	No statistical test reported	NA No clear evidence of suppression
Villegas Becerril et al. (2005)	Spain	CS	--	Cryotherapy and laser	Serum	2	--	Before and after treatment- no time-point defined	ROP stage 3	NA	NA
Wu et al. (2015)	Taiwan	P O C	December 2011–February 2013	IVB (0.625 mg)	Serum	8	1–2 d	1 d, 1, 2, 3, 4, 5, 6, 8 w	Type 1 ROP, defined as zone I, any stage ROP with plus disease (a degree of dilation and tortuosity of the posterior retinal blood vessels meeting or exceeding that of a standard photograph); zone I, stage 3 ROP without plus disease; or zone II, stage 2 or 3 ROP with plus disease. ETROP/ICROP	Paired t-test.	↓ 1, 2, 3, 4, and 6 w after-- at 1 day or 5 weeks after
Wu et al. (2017)	Taiwan	P O C	February 2013–December 2014	IVB (0.625 mg) or IVR (0.25 mg)	Serum	IVB: 6, IVR: 4	1–2 d	2, 4, 8, and 12 w	Patients with Type 1 ROP, as defined by the ETROP study/ICROP	Wilcoxon signed-rank test.	↓ at 2, 4, and 8 w after IVB-- at any w for IVR
Yang, Wang and Chen (2020)	China	P O C	December 2014 –January 2015	Laser	Serum	11	1 d	7 d	ICROP	Student t-test or Mann-Whitney U-test	↓ 7 d after
Zhou et al. (2016)	China	CS	--	IVR (0.25 mg)	Plasma	11	1 d	1 d, 1, 2, and 4 w	Stage 3+ located in zone 1 or posterior zone 2 ICROP	Student t-test, Mann-Whitney test or paired t-test	↓ on the group level was observed 1 d after

AAOP = American Academy of Ophthalmology and Pediatrics; ANOVA = analysis of variance; APOS = Association for Pediatric Ophthalmology and Strabismus; APROP = aggressive posterior retinopathy of prematurity; C = cohort; CRYO-ROP = cryotherapy for retinopathy of prematurity; CS = case series/study/report; d = day/days; ETROP = The early treatment for ROP; ICROP = International Classification of Retinopathy of Prematurity; I = interventional; IVA = aflibercept; IVB = bevacizumab; IVC = conbercept; IVR = ranibizumab; m= month/months; O = observational; P = prospective; NA = not applicable; ND = no difference; NS = not specified; PN = postnatal; R = retrospective; RCT = randomized controlled trial; --, information missing; ROP = retinopathy of prematurity; SD = standard deviation; UK = United Kingdom; US = United States; w = week/weeks; ↑ = increased, ↓ = decreased.

**Figure 3:**
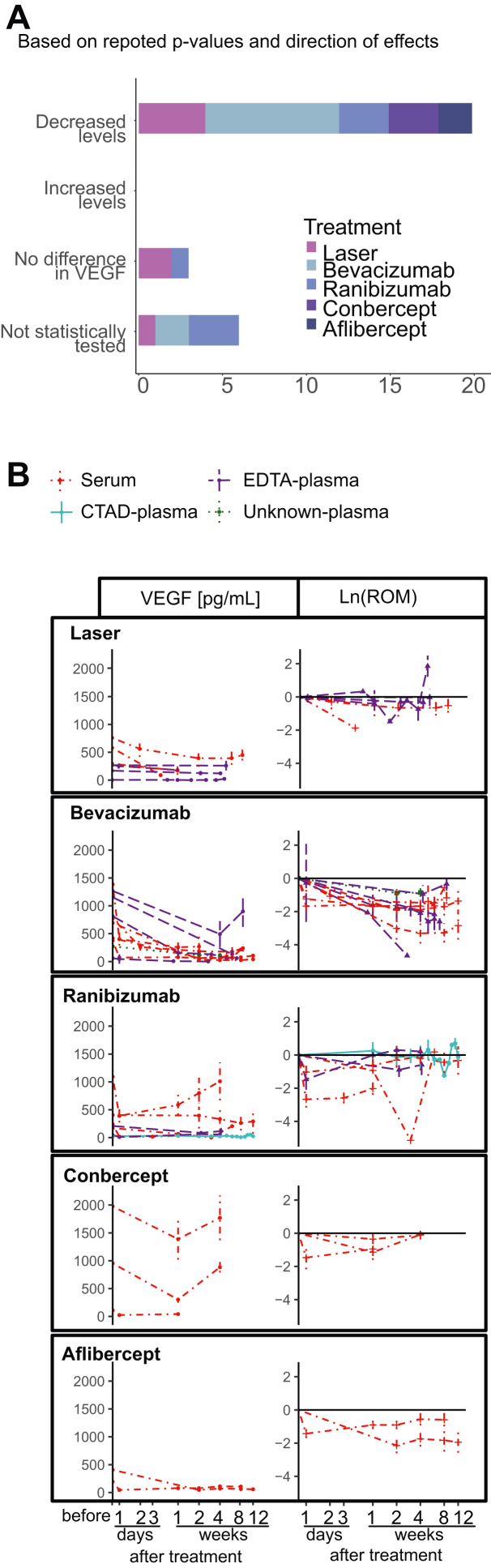
Changes in blood levels of VEGF-A after treatment of retinopathy of prematurity. (**A**) Numbers of publications per treatment and the reported change in VEGF after treatment (on ≥1 time point after treatment). (**B**) Reported mean VEGF-A concentrations per study before and after treatment, and the ratio of mean (ROM) VEGF-A from before treatment, divided on the type of treatment. CTAD = citrate, theophylline, adenosine and dipyridamole; EDTA = ethylenediaminetetraacetic acid.

In total, 24 studies were included in the meta-analysis of VEGF-A levels after ROP treatment. Seven of them evaluated laser treatment,^31^^,^^40^^,^^44^^,^^48^^,^^49^^,^^54^^,^^55^ and 20 studies explored anti-VEGF treatment,^30^, ^31^, ^32^, ^33^, ^34^, ^35^, ^36^, ^37^, ^38^, ^39^^,^^41^, ^42^, ^43^^,^^45^, ^46^, ^47^^,^^50^^,^^51^^,^^54^^,^^55^ with 3 reporting both laser and anti-VEGF treatment.^31^^,^^54^^,^^55^ One study evaluated VEGF-A levels up to 161 days after treatment;^42^ however, this study only reported levels beyond 12 weeks after treatment. Circulating VEGF-A levels were assessed after treatment with bevacizumab in 10 publications,^30^, ^31^, ^32^, ^33^, ^34^, ^35^, ^36^, ^37^, ^38^^,^^55^ ranibizumab in 7 publications,^34^^,^^41^^,^^42^^,^^45^^,^^47^^,^^50^^,^^54^ conbercept in 3 publications,^39^^,^^43^^,^^46^ and aflibercept in 2 publications.^37^^,^^51^ Direction of reported differences in VEGF-A levels after treatment are summarized in Table 2 and Figure 3A. VEGF-A concentrations per treatment and the ROM from before treatment are longitudinally depicted in Figure 3B.

### Risk of Bias for Studies Evaluating VEGF-A after ROP Treatment

**Figure 4:**
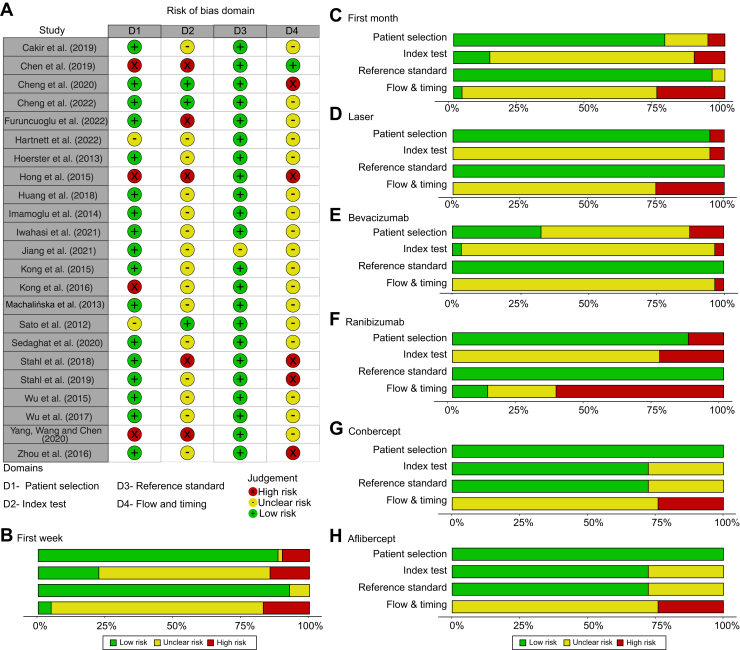
Risk of bias assessment according to Quality Assessment of Diagnostic Accuracy Studies-2 for publications evaluating VEGF-A level after retinopathy of prematurity treatment. (**A**) Overview of bias evaluation per study and domain, (**B**) summary per domain for studies evaluating VEGF-A levels in the first week after treatment (n = 16), (**C**) summary for studies evaluating VEGF-A levels in the first month after treatment (n = 23), and (**D**-**H**) summary per domain for each included treatment type. Summaries are weighted by the effective sample size (ESS) (ESS=(4n1n2)/n1+n2).

The risk of bias for studies evaluating VEGF-A after ROP treatment is depicted in Figure 4, summarizing the quality assessment of each study. One of the studies included was a conference abstract; though VEGF-A values were reported, the quality evaluation was not possible due to insufficient study details.^55^

Concerning the *patient selection* domain, 75% of the studies were deemed to have a low risk of bias. However, 4 publications^30^^,^^35^^,^^41^^,^^44^ were identified with a high risk of bias in *patient selection,* suggesting potential inclusion based on convenience sampling and a risk of enrolling infants with more severe ROP. Studies with an unclear risk lacked information on the inclusion/exclusion criteria or participant descriptions, hampering bias assessment.

In the *index test* domain, numerous studies did not report the analytical method performance. Some publications presented circulating VEGF-A levels with limited samples and few repeated longitudinal samples, leading to a high risk of bias for the *index test* domain in these studies.^35^^,^^41^^,^^42^^,^^44^^,^^51^ In the study by Hong et al.^35^ an extreme outlier was included as baseline value; however, this concentration was excluded in the meta-analysis.

Concerning the *reference standard domain*, all studies were judged to have a low risk of bias, employing commercially available assays for VEGF-A measurement. An exception was a study in Chinese,^38^ where extraction of information about the method was not possible, resulting in an unclear risk. The classification of ROP had a low risk of bias in all included publications.

High variability in systemic VEGF-A levels below detection/quantification levels and irregularities in sample frequency at follow-up after treatment contributed to a high risk of bias in the *flow and timing* domain.^35^^,^^42^^,^^43^^,^^45^^,^^54^ Additionally, most studies did not report details on sample analysis timelines, impending judgement on how authors managed methodologic uncertainty between batches or follow-up samples.

Publications on VEGF-A levels post-ranibizumab treatment exhibited the highest frequency of high bias risk concerning the *index test* and *flow and timing* domains, primarily due to numerous samples falling below detection/quantification limits and limited follow-up samples (Fig 4F).

Meta-analyses were conducted, encompassing all publications and a subset excluding 9 publications^30^^,^^35^^,^^41^, ^42^, ^43^, ^44^, ^45^^,^^51^^,^^54^ with an estimated high risk of bias, alongside data from the conference abstract.^55^

### Laser Versus Anti-VEGF Treatment

VEGF-A levels were lower in the first week after ROP treatment (laser or anti-VEGF) compared with pretreatment levels (ROM [95% CI] 0.34 [0.25–0.45], I^2^ = 97%). When comparing the impact on VEGF-A levels after laser or anti-VEGF treatment, a more substantial reduction in VEGF-A levels was observed after anti-VEGF treatment (laser 0.55 [0.32–0.97], I^2^ = 98%; anti-VEGF: 0.29 [0.21–0.40], I^2^ = 94%; subgroup difference *P* = 0.05) (Supplement Appendix 3, Fig S5A, available at www.ophthalmologyscience.org). After excluding studies with a high risk of bias and identifying one study as an outlier (Cakir et al^40^), VEGF-A levels remained reduced posttreatment, and the results were significantly different between laser and anti-VEGF treatments (laser 0.77 [0.61–0.97], I^2^ = 42%; anti-VEGF: 0.31 [0.25–0.38], I^2^ = 40%; subgroup difference *P* < 0.01) (Supplement Appendix 3, Fig S5B, available at www.ophthalmologyscience.org).

Additionally, systemic VEGF-A levels were consistently reduced 1 month after any ROP treatment (0.35 [0.28–0.43], I^2^ = 96%). The reduction of VEGF-A levels after the first month was more prominent following anti-VEGF treatment (laser 0.56 [0.38–0.83], I^2^ = 98%; anti-VEGF: 0.32 [0.25–0.41], I^2^ = 95%; subgroup difference *P* = 0.02) (Supplement Appendix 3, Fig S6, available at www.ophthalmologyscience.org).

### Comparing Treatment with Different Anti-VEGF Drugs

There was no significant difference in the reduction of VEGF-A in the first week among various anti-VEGF drugs: bevacizumab (0.25 [0.19–0.32] I^2^ = 2%), ranibizumab (0.28 [0.13–0.61] I^2^ = 96%), conbercept (0.39 [0.24–0.63] I^2^ = 86%), and aflibercept (0.31 [0.19–0.52] I^2^ = 87%), subgroup difference *P* = 0.38 (Supplement Appendix 3, Fig S7A, available at www.ophthalmologyscience.org). Exclusion of publications deemed to have a high risk of bias did not alter the results. However, aflibercept was no longer included in the comparison, and while the heterogeneity increased for bevacizumab, it decreased for ranibizumab and conbercept (bevacizumab I^2^ = 15%, ranibizumab I^2^ = 45%, conbercept I^2^ = 0%) (Supplement Appendix 3, Fig S7B, available at www.ophthalmologyscience.org).

In the first month posttreatment, a significant difference was observed among anti-VEGF drugs (subgroup difference *P* < 0.01). Bevacizumab exhibited the numerically highest reduction (0.19 [0.15–0.25] I^2^ = 83%), followed by aflibercept (0.28 [0.18–0.45], I^2^ = 89%). Ranibizumab and conbercept showed a similar reduction (ranibizumab: 0.53 [0.34–0.82], I^2^ = 96%; conbercept: 0.53 [0.34–0.84] I^2^ = 94%) (Supplement Appendix 3, Fig S8, available at www.ophthalmologyscience.org).

### Length of VEGF-A Reduction after ROP Treatment

**Figure 9:**
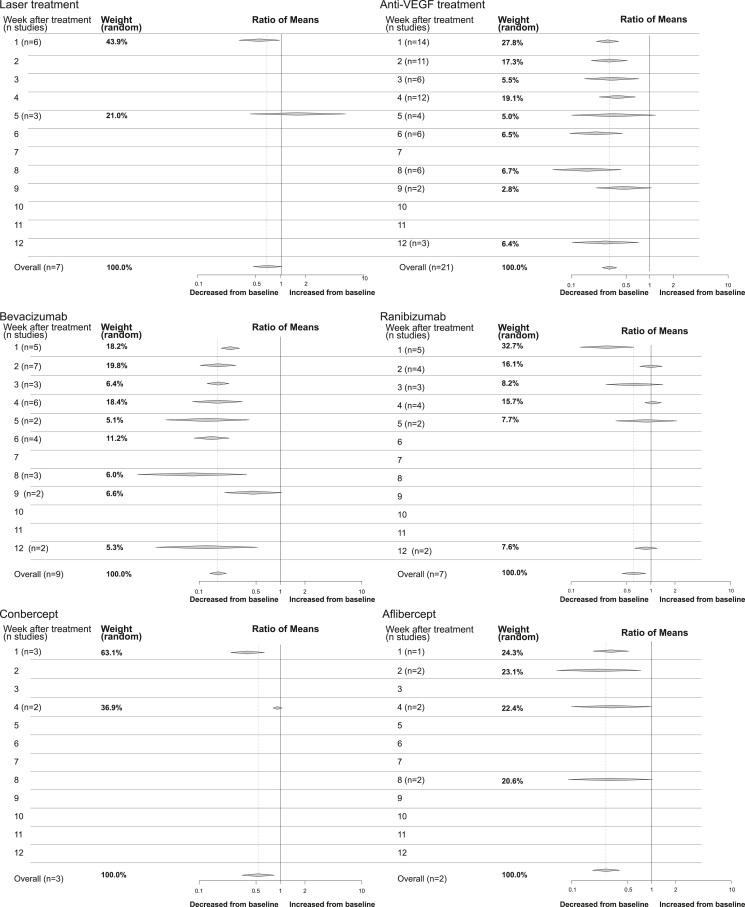
Decreased levels of VEGF-A compared before and after retinopathy of prematurity (ROP) treatment. Including all reported VEGF-A levels until 12 weeks after ROP treatment, the random model calculated meta-analysis was used to calculate the ratio of means per week, and the number of included studies is reported. Note that each publication may report on several measurements per week. Results reported for laser treatment: all anti-VEGF drugs, bevacizumab treatment, ranibizumab treatment, conbercept treatment, and aflibercept treatment.

The length of VEGF-A reduction following ROP treatment was investigated through a comprehensive analysis. Four publications reported decreased VEGF-A levels within the initial week postlaser treatment,^31^^,^^40^^,^^44^^,^^48^ with 1 study indicating sustained reduction up to 60 days after treatment.^31^ Meta-analysis corroborates reduced VEGF-A levels within the first week after treatment; however, no statistically significant reduction was observed at week 5 posttreatment (Fig 9 and Supplement Appendix 3, Fig S10, available at www.ophthalmologyscience.org).

In the case of anti-VEGF treatment, a meta-analysis indicated decreased VEGF-A levels within the first 4 weeks posttreatment, with a significant reduction observed at weeks 6, 8, and 12. Nevertheless, weeks 5 and 9 did not exhibit a significant reduction (Fig 9 and Supplement Appendix 3, Fig S11, available at www.ophthalmologyscience.org).

Huang et al^37^ reported sustained reduction in VEGF-A levels up to 12 weeks post-bevacizumab treatment. However, a study by Wu et al^34^ did not show a significant reduction at this time point. Meta-analysis demonstrated reduced levels up to 8 weeks post-bevacizumab treatment, with no significant reduction at week 9 but a significant reduction at week 12 (Fig 9 and Supplement Appendix 3, Fig S12, available at www.ophthalmologyscience.org).

For ranibizumab, 3 studies reported decreased VEGF-A levels within the first week posttreatment,^41^^,^^45^^,^^47^ and 1 case study reported reduced levels 3 weeks after treatment.^50^ Conversely, 3 studies reported no differences in VEGF-A levels post-ranibizumab treatment,^34^^,^^42^^,^^54^ with 2 studies not evaluating the first week posttreatment.^34^^,^^54^ Meta-analysis indicated reduced VEGF-A levels only within the first week post-ranibizumab treatment (Fig 9 and Supplement Appendix 3, Fig S13, available at www.ophthalmologyscience.org).

All included studies uniformly reported significantly reduced VEGF-A levels during the first week post-conbercept treatment.^39^^,^^43^^,^^46^ However, 2 studies evaluating levels after 4 weeks found no reduction in VEGF-A levels at this time point.^43^^,^^46^ The meta-analysis revealed similarly reduced VEGF-A levels in the first week but no reduction at week 4 after conbercept treatment (Fig 9 and Supplement Appendix 3, Fig S14, available at www.ophthalmologyscience.org).

After aflibercept treatment, 1 study reported VEGF-A levels to be reduced for 8 weeks,^51^ while another reported sustained reduction for 12 weeks.^37^ Meta-analysis of these studies indicated reduced levels at 1–2 weeks post-aflibercept treatment, but insignificant changes at weeks 4 and 8 (Fig 9 and Supplement Appendix 3, Fig S15, available at www.ophthalmologyscience.org).

### Dependency of the Blood Sampling System to Identify a Reduction in VEGF-A Levels after ROP Treatment

During the first week following treatment, serum showed a more pronounced decrease in VEGF-A than plasma samples (serum 0.27 [0.20–0.36], I^2^ = 94%; plasma 0.63 [0.38–1.03]; I^2^ = 85%, subgroup difference *P* < 0.01) (Supplement Appendix 3, Fig S16A, available at www.ophthalmologyscience.org). The mean number of days from treatment to the follow-up samples was 5.0 days for plasma and 4.9 days for serum. Excluding publications with a high risk of bias reduced heterogeneity, but only 1 publication reporting VEGF-A levels in plasma was included (Supplement Appendix 3, Fig S16B, available at www.ophthalmologyscience.org).

Comparison of samples taken in the first month after treatment revealed an overall more pronounced decrease in VEGF-A levels measured in serum than in plasma (serum 0.28 [0.21–0.36] I^2^ = 96%; plasma 0.54 [0.40–0.73]; I^2^ = 91%; subgroup difference *P* < 0.01, Supplement Appendix 3, Fig S17, available at www.ophthalmologyscience.org). The mean number of days after treatment was 15 for plasma and 12 for serum.

**Figure 18:**
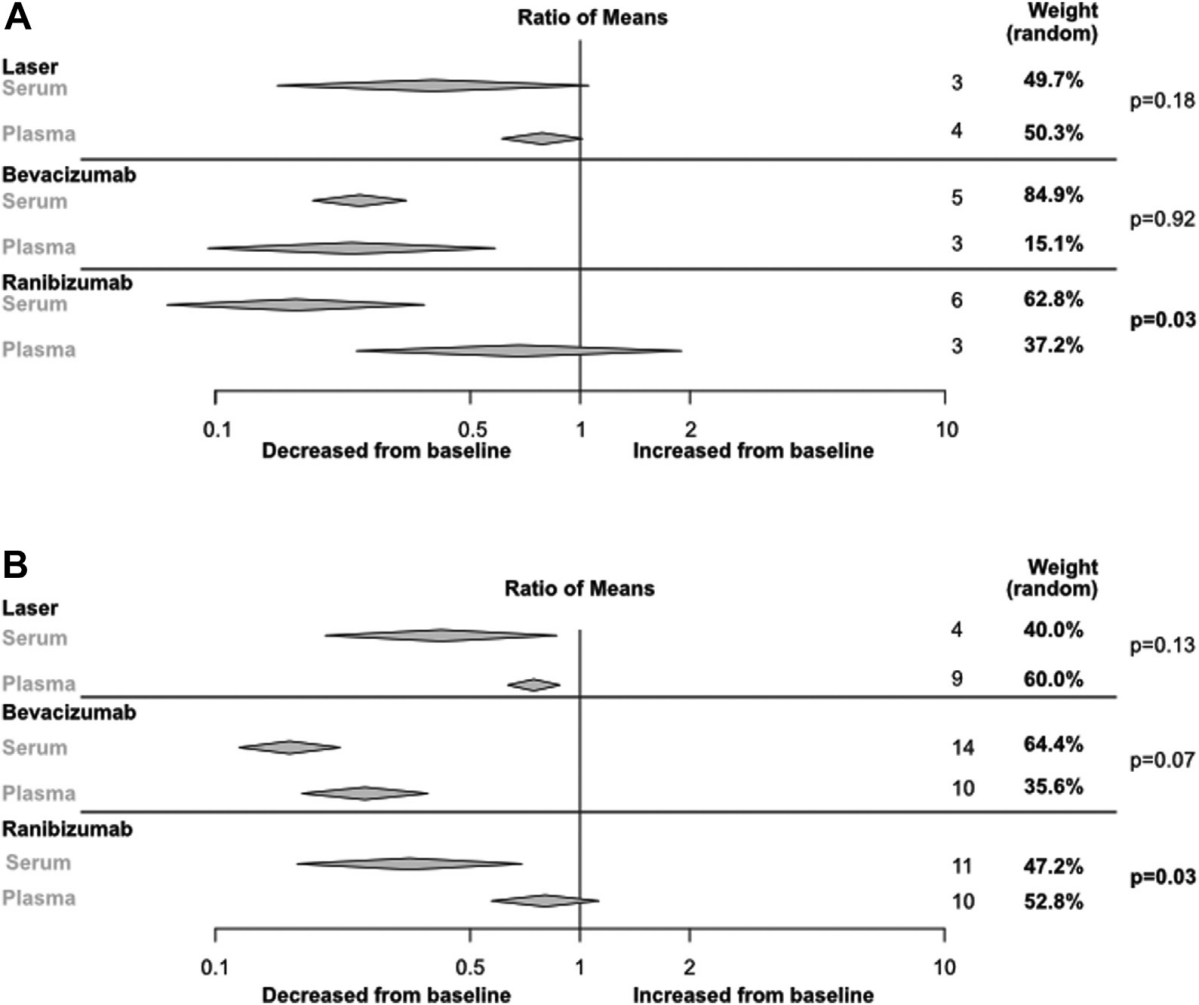
Changes in serum or plasma VEGF-A levels after treatment with laser and different anti-VEGF drugs. The ratio of means for VEGF levels after treatment for retinopathy of prematurity against before treatment was compared between serum and plasma samples within each treatment type. The comparison includes VEGF levels reported (**A**) within the first week after treatment and (**B**) within the first month after treatment. Analysis was performed with random model meta-analysis with metacont in R-studio using the meta-package.

Plasma and serum samples were utilized to investigate VEGF-A levels after treatment with laser, bevacizumab, and ranibizumab (Fig 18). For laser and bevacizumab treatments, no differences in reduction were observed between plasma and serum samples in the first week or month after treatment (Supplement Appendix 3, Figs S19–S22, available at www.ophthalmologyscience.org). However, for ranibizumab, the reduction was more pronounced in serum than in plasma, both in the first week and month after treatment (subgroup difference first week and first month *P* = 0.03) (Supplement Appendix 3, Figs S23–S24, available at www.ophthalmologyscience.org). Plasma and serum samples were collected within the first week after ranibizumab treatment at an average of 5.8 days for plasma and 4.2 days for serum, and plasma and serum samples were collected during the first month after ranibizumab treatment at an average of 16 and 12 days, respectively.

### Publication Bias for Studies Evaluating VEGF-A after ROP Treatment

The evaluation of publication bias in studies reporting VEGF-A levels during the first week after ROP treatment was conducted. Egger’s test did not indicate publication bias when considering all publications (t = 1.47, *P* = 0.155) (Supplement Appendix 3, Fig S25, available at www.ophthalmologyscience.org). However, a detailed examination of publication bias per treatment during the first week revealed asymmetry in the Funnel plot for laser treatment, and the test was statistically significant (t = 2.95, *P* = 0.042) (Supplement Appendix 3, Fig S26A and B, available at www.ophthalmologyscience.org). Subsequent exclusion of the study by Cakir et al,^40^ identified as an outlier, resulted in a nonsignificant test (t = −0.41, *P* = 0.708, Supplement Appendix 3, Fig S26C and D, available at www.ophthalmologyscience.org). For bevacizumab, the Funnel plot did not indicate publication bias (t = −0.30, *P* = 0.774, Supplement Appendix 3, Fig S27, available at www.ophthalmologyscience.org). Despite large variability in the result for ranibizumab, the test for asymmetry did not suggest publication bias (t = −1.53, *P* = 0.176, Supplement Appendix 3, Fig S28, available at www.ophthalmologyscience.org). Aflibercept and conbercept were excluded from evaluation due to the limited number of included publications (<5).

### VEGF-A Levels as a Biomarker for ROP

We included 31 publications that reported VEGF levels in ROP cases compared with those in a control group^30^^,^^40^^,^^55^, ^56^, ^57^, ^58^, ^59^, ^60^, ^61^, ^62^, ^63^, ^64^, ^65^, ^66^, ^67^, ^68^, ^69^, ^70^, ^71^, ^72^, ^73^, ^74^, ^75^, ^76^, ^77^, ^78^, ^79^, ^80^, ^81^, ^82^, ^83^ (Tables 3 and 4). One study lacked information about the time-point for sampling,^82^ 1 only reported results for VEGF-C,^78^ and 1 did not report ROP stages that could be interpreted^57^; these studies are excluded from further analysis but are presented in Table 3. Three studies did not report VEGF concentrations,^72^, ^73^, ^74^ and 1 did not report the blood sampling system used;^65^ these are excluded from the meta-analysis.

**Table 3:** Included Publications Comparing VEGF-A Levels Between ROP and Control at Different PNAges

Study	Country	Study Design	Time for Study	Sample System	Definition of ROP	GA at Birth ROP	Specification of Control	GA at Birth Control	N-ROP	N-Control	Sample Taken	Statistical Test	Direction of Difference for ROP vs Control
Aydogan et al. (2022)	Turkey	P O C	June 2017–June 2018	Serum	Untreated ROP, treated ROP NS	Mean (IQR) ROP- untreated: 28 (27–29) weeks ROP-treated: 27 (25.5–28.5) weeks	No-ROP preterm	Mean (IQR): 29 (27–30) weeks	7 untreated, 5 treated	25	Within first 5 d and at first examination (no information about age at examination)	Kruskal-Wallis test	ND
Babaei et al. (2019)	Iran	P O C	October 2014–November 2017	Serum	ROP AAOP and APOS/ICROP	Mean (SD) 31.3 (0.9) weeks	No-ROP preterm and term infants	Mean (SD) preterm 31.3 (0.9) weeks, term 38.3 (0.9) weeks	22	Preterm 35, full-term 42	At birth	One-way ANOVA with Tukey post hoc	↑ ROP compared to preterm and term control,
Bartkevičienė et al. (2021)	Lithuania	P O C	2007–2010	Plasma	All stages, ICROP	Median (q1-q3) 28.0 (26–30) weeks	No-ROP	Median (q1-q3) 33.0 (32–34) weeks	37	112	At birth, cord blood	Student t-test or Mann-Whitney U-test	ND
Berrington et al. (2012) (abstract)	UK	C	2011–2012	Plasma	All stages (1, 2 and 3) NS	Median (range) 26 (23–29) weeks	No-ROP	Median (range) 27 (25–29) weeks	22	5	PN d 1–134	Not tested	NA
Cakir et al. (2019)	Turkey	P O C	February 2017–August 2018	Serum	Severe ROP and treated ICROP	Mean (SD, range) 26.3 (1.5, 24–28) weeks	No-ROP preterm	Mean (SD, range) 29.3 (1.9, 25–30) weeks	73	73	At birth and at PN 4 w	Mann-Whitney U-test	↑ for ROP at birth and PN 4 w
Demir et al. (2013)	Turkey	P O C	--	Serum?	ROP stage 2–3, threshold and prethreshold ICROP	Mean (SD) 30.5 (2.3) weeks	No-ROP or ROP stage 1 in zone 3 preterm	Mean (SD) 30.5 (2.3) weeks	11	11	7 w	Student t-test or Mann-Whitney U-test	ND
Du, Chen and Shi (2010)	China	P O C	January–August 2005	Serum	ROP ICROP	Mean (SD) 29.8 (0.8) weeks	No-ROP preterm <32 weeks, 32-<37 weeks and full-term, further divided according to oxygen-group	Mean (SD) preterm, <32 weeks: 30.4 (0.14/1.4) weeks, 32-<37 weeks: 34.2 (1.3), full-term: (39.7 (1.4) weeks	6	37	First w of life, w 3, 5 and 7	Unpaired t-test and one-way ANOVA	↑ VEGF-A levels in preterm (32–36 weeks) compared with term at week 1 and 3, and for infants born <32 weeks compared with term at week 1.
Feng et al. (2017)	China	P O C	--	Plasma	ROP (all stages) and severe ROP (Type 1 prethreshold and threshold, stages 4 and 5) and mild ROP ICROP	--	No-ROP preterm	--	53	12	--	NS	↓
Hellgren et al. (2016)	Sweden	P O C	January 2005–May 2007	Plasma	Nonproliferative ROP, treated for ROP ICROP	Median (range) nonproliferative 25.1 (23.4–26.4) weeks. Treated for ROP 24.1 (23.0–27.1) weeks	No-ROP preterm	Median (range) 27.4 (24.3–30.6) weeks	nonproliferative ROP 9, treated for ROP 5	25	At birth	Kruskal-Wallis test	ND
Holm et al. (2017)	US	P O C	2002–2004	Whole blood dried blood spot	Severe ROP (prethreshold) ICROP	41% 23–24 weeks 51% 25–26 weeks 7% 27 weeks	No ROP	18% 23–24 weeks 46% 25–26 weeks 36% 27 weeks	168	1037	PN day 1, 7, 14, 21 and 28	Logistic regression models to calculate OR and 95% CI. Adjusted for GA and birthweight z-score <1.	↓ High levels of VEGF-A on d 7 was associated with a reduced risk for prethreshold ROP
Katsan et al. (2019)	Ukraine	P O C	--	Serum	Progressive or stable ROP NS	Mean (SD) 29.2 (1.8) weeks	Regressive ROP	Mean (SD) 30.7 (1.5) weeks	15	8	At first examination and follow up at 14 d after	Student t-test or Wilcoxon test.	ND between groups at follow-up
Kwinta et al. (2008)	Poland	P O C	May 2003–October 2006	Serum	ROP not requiring treatment, ROP requiring treatment according to current recommendations NS	Mean (SD) not requiring treatment 27.5 (1.6) weeks, requiring treatment 26.7 (2.3) weeks	No-ROP preterm	Mean (SD) 29.2 (2.05) weeks	Not requiring treatment 20 Requiring treatment 60	101	PN 10, 20, 30–40 d	Kruskal-Wallis test	↓ for ROP requiring treatment at all PN time-points
Machalińska et al. (2010)	Poland	P O C	--	Plasma	ROP- proliferative, stage 3 and above ICROP	Mean (SD) 27.1 (2.5) weeks	No-ROP preterm, no-ROP term	Mean (SD) preterm 30.5 (2.7) weeks, full-term 39.2 (1.1) weeks	29	Preterm 29, full-term 30	PNA 10 w	Kruskal-Wallis test followed by Mann-Whitney U-test results adjusted for GA with multiple regression with logarithmic transformation	↑ VEGF-A compared to preterm control group, but not statistically higher compared with term infants.
Markasz et al. (2020)	Sweden	P O C	November 2012–May 2015	Serum	ROP stage 1–3 ICROP	Mean (95% CI) 24.6 (23.5–25.6) weeks summarized from figure	No-ROP	Mean (95% CI) 26.3 (25.6–27) weeks Median (IQR) 26.6 (26.4–27.1) weeks From figure	23	12	PN day 2	One-way analysis of variance tests to evaluate differences between the hierarchically clustered groups. Differences analyzed with 2-sided paired Student t-test and Bonferroni corrections	↓ for ROP than non-ROP when non-ROP were divided in subgroups.
Movsas and Muthusamy (2020)	US	Pilot study- CC	August 2012–March 2015	Whole blood dried blood spot	Nonproliferative ROP ICD 9 codes	24–28 weeks	No-ROP preterm	24–28 weeks	27	38	Within first w of life	Mann-Whitney U-test, *P* < 0.05, trend *P* < 0.15	↑ VEGF-165 for males with ROP
Peirovifar et al. (2013)	Iran	CC	March 2009–June 2010	NS	Proliferative ROP ICROP	Mean (SD, range) at birth 28.4 (1.6, 26–32) weeks	No-ROP preterm	Mean (SD, range) at birth 28.8 (1.6, 26–34) weeks	30	41	PN 6–8 w	T-test or Mann-Whitney U-test	ND
Qi et al. (2013)	China	P O C	October 2010–June 2011	Plasma	ROP NS	Whole cohort Mean (SD) 29.5 (1.7) weeks	No-ROP preterm	whole cohort Mean (SD) 29.5 (1.7) weeks	13	47	At birth- peripheral blood	Student t-test or Mann-Whitney U-test	ND
Sehgal et al. (2022)	India	P O C	January–October 2020	Serum	ROP requiring treatment as per ETROP criteria (Type1 ROP) and ICROP	Mean (SD) 30.2 (1.8) weeks	Regressed ROP/no ROP/fully vascularized retina	Mean (SD) 32.0 (1.5) weeks	35	36	First screening, 3–4 weeks PN age	Wilcoxon rank sum test	ND
Villegas-Becerril et al. (2006)	Spain	P O C	October 2003–January 2005	Serum	ROP AAOP and APOS/ICROP	Mean (SD) 29.21 (2.46) weeks	No-ROP	Mean (SD) 31.23 (2.45) weeks	37	37	At ROP screening	Student t-test	↑
Woo et al. (2013)	South Korea	R age matched CC	June 2004–July 2010	Plasma	ETROP (all stages) AAOP and APOS/ICROP	Mean (SD) 29.0 (1.3) weeks	No-ROP preterm	Mean (SD) 28.9 (1.9) weeks	20	40	At birth- cord blood	Conditional logistic regression analysis for matched case-control with logarithmic values	ND
Yenice et al. (2013)	Turkey	P O C	June 2006–October 2008	Serum	ROP (all stages), ROP stage 1, 2 and 3, AAOP and APOS/ICROP	Mean (SD) 29.65 (2.34) weeks	No-ROP preterm	Mean (SD) 32.22 (1.49) weeks	57, 23 stage 1, 27 stage 2, 7 stage 3	36	At birth- cord blood	Mann-Whitney U-test, Kruskal-Wallis test	↓ No significant association with ROP stage (*P* = 0.058)
Zepeda-Romero et al. (2017)	Mexico	P CC	February 2012–January 2013	Serum	ROP all stages ICROP	Mean (SD) at birth 30.2 (0.3) weeks	No-ROP preterm	Mean (SD) at birth 30.8 (0.3) weeks	49	41	PN 1–6 w	T-test or Mann-Whitney U-test, longitudinal development by Kruskal-Wallis test and 1-way ANOVA and Bonferroni’s post hoc	ND
Zhang et al. (2022)	China	P CC	January 2016–January 2018	Plasma	Any stage of ROP, ICROP	Mean (SD) 29.5 (2.0) weeks	No ROP	Mean (SD) 29.4 (2.0) weeks	33	33	PN weeks 4–6	Student t-test or Mann-Whitney U-test	↑
Zhu, Chen and Shi (2015)	China	R CC	July 2006–January 2007	Serum	ROP CRYO-ROP/ICROP	Mean (SD) 29.2 (1.73) weeks	No-ROP preterm	Mean (SD) 29.7 (1.28) weeks	11	43	PN 7, 14, 21, 28 and 35 d	Mann-Whitney U-test	↓ at PND 7, 14 and 21,
Yang et al. (2017)	China	P CC	March 2014–September 2014	Serum	ROP stage 2 or more according to ICROP	Mean (SD) 29.3 (1.73) weeks	No-ROP	29.1 (0.90) weeks	6	4	PN weeks 4–6	Student t-test or Mann-Whitney U-test	↓ VEGF-C

AAOP = American Academy of Ophthalmology and Pediatrics; ANOVA = analysis of variance; APOS = Association for Pediatric Ophthalmology and Strabismus; C = cohort; CC = case-control; CI = confidence interval; CRYO-ROP = cryotherapy for retinopathy of prematurity; d = day/days; ETROP = early treatment for ROP; GA = gestational age; ICD = International Classification of Diseases; ICROP = International Classification of Retinopathy of Prematurity; IQR = interquartile range; NA = not applicable; ND = no difference; NS = not specified; O = observational; OR = odds ratio; P = prospective; PN = postnatal; PNA = postnatal age; PND = postnatal day; R = retrospective; w = week/weeks; --, Information missing; ROP = retinopathy of prematurity; SD = standard deviation; UK = United Kingdom; US = United States; ↑ = increased; ↓ = decreased.

**Table 4:** Included Publications Evaluating VEGF Levels Between ROP and Control at Different PMAs

Study	Country	Study Design	Time for Study	Sample System	Definition of ROP	GA at Birth ROP	Specification of Control	GA at Birth Control	n-ROP	n-control	Sample Taken	Statistical Test	Direction of Difference for ROP vs Control
Berrington et al (2012) (abstract)	UK	C	2011–2012	Plasma	All stages (1, 2 and 3) NS	Median (range) 26 (23–29) weeks	No-ROP	Median (range) 27 (25–29) weeks	22	5	PMA w 26–43	Not tested	NA
Du, Chen and Shi (2010)	China	P O C	January–August 2005	Serum	ROP ICROP	Mean (SD) 29.8 (0.8) weeks	No-ROP preterm = oxygen and no-oxygen group	Mean (SD) 30.4 (0.14/1.4) weeks	6	37	Each w 28–40	Unpaired t-test	↑ at PMA 31 weeks,
Cakir et al (2019)	Turkey	P O C	February 2017–August 2018	Serum	Severe ROP and treated ICROP	Mean (SD, range) 26.3 (1.5, 24–28) weeks	No-ROP preterm	Mean (SD, range) 29.3 (1.9, 25–30) weeks	73	73	PMA 34 w corrected age	Mann-Whitney U-test	↑
Feng et al (2022)	China	CrossSec, P, O	--	Plasma	Mild and severe (severe = type 1, stages 4 and 5). ICROP	Mean (SD) 30.3 (2.1) weeks	No-ROP	Mean (SD) 31.4 (1.5) weeks	50	50	PMA 38–39 w	Mann-Whitney U-test	↓ for infants with ROP compared with no-ROP. No differences between mild and severe ROP
Goswami et al (2015)	India	O comparative study	No information	Serum	ROP ICROP	Mean (SD) 29.4 (1.99) weeks	No-ROP preterm	Mean (SD) 31.6 (1.99) weeks	30	30	32–34 w and 38–40 w	Mann-Whitney U-test or student t-test	ND
Kong et al (2016)	US	P O C	No information	Plasma	Type 1 ROP AAOP and APOS/ICROP	Mean (SD): 25.6 (0.6) weeks	No-ROP preterm	Mean (SD): 28.0 (0.6) weeks	13	13	ROP before treatment mean (SD) PMA 34.9 (1.7) w and corresponding age for control	Not tested	NA
Hellgren et al (2016)	Sweden	P O C	January 2005–May 2007	Serum	Nonproliferative ROP, treated for ROP ICROP and ETROP	Median (range) nonproliferative 25.1 (23.4–26.4) weeks treated for ROP 24.1 (23.0–27.1) weeks	No-ROP preterm	Median (range) 27.4 (24.3–30.6) weeks	Nonproliferative 10, treated 9	33	Each w PMA 23–40	Kruskal-Wallis test	↑ for infants treated for ROP at PMA 34, 35 and 36 weeks,
Hellgren et al (2021)	Sweden	R O C	April 2013–September 2015	Serum	Severe ROP ICROP and ETROP	Mean (SD) 24.3 (1.1) weeks	No/mild ROP	Mean (SD) no-ROP 26.1 (1.4) weeks Mild ROP 25.4 (1.1) weeks	31	47	Each w 24–29 and 32, 36, and 40 w	Mann-Whitney U-test	↓ at PMA 32 and 36 weeks compared with no/mild ROP
Kandasamy et al (2018)	Australia	P CC	July 2014–July 2016	Serum	ROP ETROP/ICROP	Median (range) 25.4 (24.2–26.0) weeks	No-ROP preterm	Median (range) 27.1 (26.8–27.9) weeks	33	20	32–36 w	Mann-Whitney U-test or student t-test	ND
Kwinta et al (2008)	Poland	P O C	May 2003–October 2006	Serum	ROP not requiring treatment, ROP requiring treatment Current recommendations	Mean (SD) not requiring treatment 27.5 (1.6) weeks requiring treatment 26.7 (2.3) weeks	No-ROP preterm	Mean (SD) 29.2 (2.05) weeks	Not requiring treatment 20 requiring treatment 60	101	Each w PMA 28–34	One-way ANOVA or Kruskal-Wallis test	↑ for PM week 30 and 31 for both ROP groups vs. controls
Pieh et al (2008)	Germany	P O C	No information	Plasma	ROP CRYO-ROP/ICROP	Mean (range) 25.5 (23.1–29.3) weeks	No-ROP preterm	Mean (range) 28.82 (25.6–32.6) weeks	21	42	W 32 and 36	Kernel smoothing was applied to fit the data with a Gaussian kernel bandwidth of 2.1 days. To assess statistically significant differences in the time course of plasma levels, a subgroup with pairs of measurements for all angiogenic factors were used. Possible significant differences were assessed by analysis of variance with repeated measures using as factors.	ND

AAOP = American Academy of Ophthalmology and Pediatrics; APOS = Association for Pediatric Ophthalmology and Strabismus; C = cohort; CC = case-control; CrosSec = cross-sectional; CRYO-ROP = cryotherapy for retinopathy of prematurity; d = day/days; ETROP = early treatment for ROP; GA = gestational age; ICROP = International Classification of Retinopathy of Prematurity; NA = not applicable; ND = no difference; NS = not specified; O = observational; P = prospective; PM = postmenstrual; PMA = postmenstrual age; R = retrospective; w = week/weeks; --, Information missing; ROP = retinopathy of prematurity; SD = standard deviation; UK = United Kingdom; US = United States; ↑ = increased; ↓ = decreased.

Four of the included publications compared VEGF-A levels in infants with ROP with term controls.^43^^,^^61^^,^^69^^,^^76^ However, no meta-analysis was performed for this study setup because the time of measurement differed between these studies; however, 1 of these studies by Cheng et al ^43^ was included in the meta-analysis of VEGF-A levels in relation to ROP treatment.

### Risk of Bias Assessment of Publications Reporting VEGF-A Levels as a Biomarker for ROP

**Figure 29:**
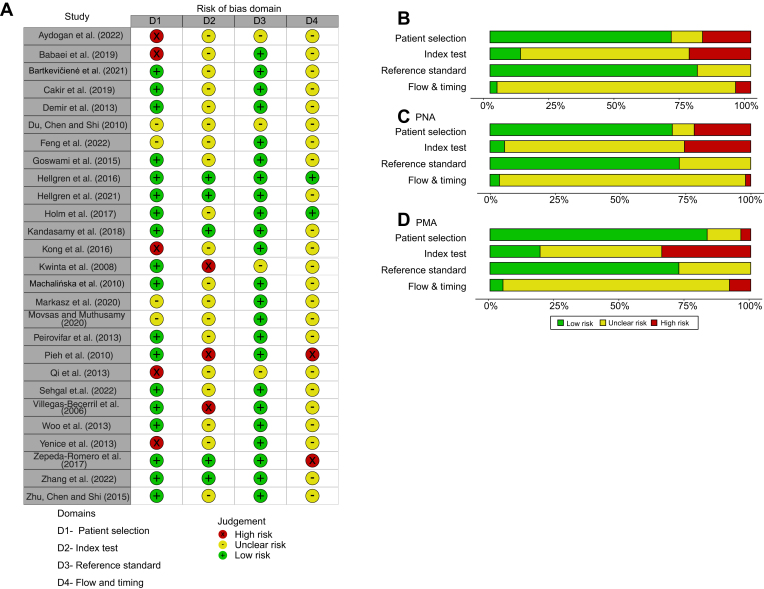
Risk of bias assessment according to Quality Assessment of Diagnostic Accuracy Studies-2 for publications evaluating VEGF-A level concerning retinopathy of prematurity development. (**A**) Overview of bias evaluation per study and domain, (**B**) summary per domain for all 27 included publications, (**C**) summary for studies evaluating on a PNA time scale, and (**D**) summary for studies evaluating on a PMA time scale. PNA = postnatal age; PMA = postmenstrual age; ROP = retinopathy of prematurity.

The quality rating for studies evaluating VEGF-A as a biomarker for ROP is depicted in Figure 29. One study included was a conference abstract ^55^ and although VEGF-A values from this publication were reported, the quality assessment could not be conducted due to insufficient study details.

The *patient selection* domain demonstrated a low risk of bias in >75% of the included publications. However, 4 publications^61^^,^^63^^,^^74^^,^^83^ lacked sufficient information for domain evaluation, and 5^30^^,^^56^^,^^58^^,^^59^^,^^76^ were deemed to have a high risk, primarily because of the risk of convenience sampling potentially impacting the results. Regarding the *index test* domain, most publications lacked adequate information on the analytical method, making risk of bias assessment challenging. Three publications^62^^,^^68^^,^^79^ were identified as having a high risk due to comparing unequal groups without result adjustment or using pooled samples without specifying gestational or postnatal ages.

In the *reference standard* domain, the overall risk of bias was deemed low. However, some publications^56^^,^^58^^,^^68^ did not report the standard used for ROP classification, and 1 study in Chinese ^61^ was classified as having an unclear risk.

Crucial information regarding sample collection and measurement timing was largely unreported in most publications, making it impossible to assess *the flow and timing* domain. Two publications^66^^,^^79^ received a high risk of bias in this domain because not all mentioned samples in the methods reported results, and no dropout information was provided (Fig 29).

Meta-analyses were conducted, encompassing all publications, and a subset excluding 9 publications^30^^,^^56^^,^^58^^,^^59^^,^^62^^,^^66^^,^^68^^,^^76^^,^^79^ with a high risk of bias and data from a conference abstract^55^ (if the total number of included VEGF-A levels allowed).

### VEGF-A Concentration in Relation to ROP Using a Postnatal Timescale

The concentration of VEGF-A in relation to ROP was examined using a postnatal timescale in 22 publications^40^^,^^55^^,^^56^^,^^58^, ^59^, ^60^, ^61^, ^62^, ^63^, ^64^^,^^66^, ^67^, ^68^, ^69^, ^70^, ^71^, ^72^, ^73^, ^74^, ^75^, ^76^, ^77^ to compare VEGF-A levels between the ROP and control groups (Table 3). Movsas and Muthsamy^63^ investigated VEGF isoforms, utilizing a specific assay for VEGF121, and one developed for VEGF165; the assay for VEGF165 was employed as the method corresponding to VEGF-A.

**Figure 31:**
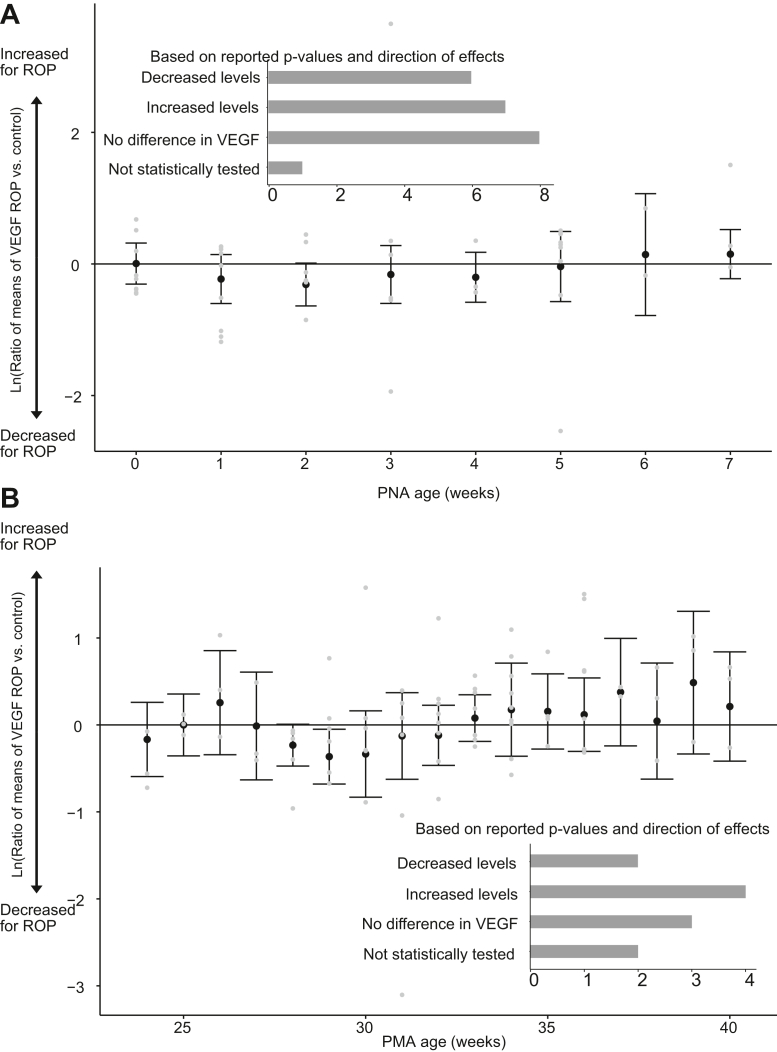
No apparent difference in serum VEGF-A levels between infants with ROP and control. Ratio of means, calculated by random model meta-analysis black dots, shows mean values and error bars 95% CI. Individual study results are illustrated by gray dots. Bar charts show the number of included publications and reported difference between ROP and a preterm control (on ≥1 reported time point). (**A**) VEGF-A values reported on a postnatal time scale are illustrated at birth (0) and postnatal weeks 1 to 7, and (**B**) VEGF-A values reported on a postmenstrual time scale, weeks 24 to 40. CI = confidence interval; PNA = postnatal age; PMA = postmenstrual age; ROP = retinopathy of prematurity.

In the meta-analysis summarizing the difference in VEGF-A levels between ROP infants and a preterm control group without ROP, 19 publications were included. However, no clear trend in VEGF-A levels in relation to ROP diagnosis was observed (0.92 [0.80-1.06] with I^2^ = 90%) (Supplement Appendix 3, Fig S30A, available at www.ophthalmologyscience.org). No significant differences were identified among the postnatal periods (at birth and weeks 1-7). In addition, a similar number of publications reported increased or decreased levels for groups with ROP compared with control (Fig 31A). Excluding publications with a high risk of bias did not affect the overall results (Supplement Appendix 3, Fig S30B, available at www.ophthalmologyscience.org).

Subsetting the 10 studies reporting VEGF-A, concentrations for severe ROP did not indicate an overall tendency for increased or decreased VEGF levels compared with infants without ROP (0.85 [0.66–1.09] I^2^ = 94%). No significant differences were identified among postnatal periods (Supplement Appendix 3, Fig S32, available at www.ophthalmologyscience.org).

Among the samples collected at birth, plasma was evaluated in 4 studies, and serum in 5 studies. The meta-analysis did not show any difference between the sample systems (subgroup difference *P* = 0.70), and no significant change in VEGF levels for infants with ROP was identified in either of the systems (Supplement Appendix 3, Fig S33, available at www.ophthalmologyscience.org).

### VEGF-A Concentration in Relation to ROP Using a Postmenstrual Timescale

A postmenstrual timescale was employed to compare VEGF-A levels between infants with ROP and controls in 11 studies^30^^,^^40^^,^^55^^,^^61^^,^^64^^,^^68^^,^^70^^,^^79^, ^80^, ^81^^,^^83^ (Table 4). No discernible trend of reduced or increased VEGF-A levels was observed in relation to ROP (0.98 [0.87–1.10] with I^2^ = 93%) and included publications reported both increased as decreased levels for ROP (Fig 31B). A significant difference was identified among different postmenstrual weeks (*P* subgroup difference <0.01), but excluding publications with a high risk of bias rendered differences among postmenstrual weeks nonsignificant (*P* = 0.73) (Supplement Appendix 3, Fig S34, available at www.ophthalmologyscience.org).

Seven studies reported VEGF-A concentrations in severe ROP; among these, 1 did not report data on the number of infants with severe ROP.^83^ Meta-analysis on the remaining 6 studies did not identify differences in VEGF-A levels between infants with or without ROP (Fig S35).

### Publication Bias for Publication Reporting VEGF-A Levels for ROP as a Biomarker

Publication bias for papers reporting VEGF-A levels on a postnatal time scale was evaluated including studies reporting levels at birth using the Eggers test. The Funnel plot indicated publication bias (t = −2.46, *P* = 0.043, Supplement Appendix 3, Fig S36, available at www.ophthalmologyscience.org). Results depicting reduced VEGF-A levels in ROP exhibited both high and low standard errors; however, increased VEGF-A levels in ROP were only reported for smaller standard errors. Publications with a high risk of bias could not be excluded due to the reported VEGF-A levels being too low.

Evaluating publication bias for papers reporting VEGF-A in relation to a postmenstrual time scale was not possible due to the low number of reported VEGF-A measurements (<5 per week).

### Summary of Findings and Quality of Evidence

Table 5 presents a summary of the findings, along with the estimated quality of evidence according to Grading of Recommendations, Assessment, Development, and Evaluation.

**Table 5:** Summary of Findings According to GRADE: Because of the Different Designs of Included Studies (It is Advised to Start With a Low grade of Evidence)

VEGF Levels Measured	Justification	Question	Number of Publications in Meta-Analysis	The Total Number of VEGF Measurements Summarized for Meta-Analysis∗	Outcome and Weighted Results; ROM [95% CI]	Specific Justification	Evidence grade
First week after treatment	1. High risk of bias for >1 domains in 9 of 16 estimated studies; however, excluding those did not change the results 2. High heterogeneity but reduced when publications with a high risk of bias were excluded, see above 3. No indirectness of evidence 4. Low imprecision 5. Low to moderate risk of publication bias 6. No dose-response gradient	Does ROP treatment affect VEGF-A levels?	17	441	Reduced VEGF-A levels after treatment 0.34 [0.25–0.45]	+1 large magnitude of effect +1 based on common effect and the magnitude of the effect. VEGF-A levels after treatment was significantly reduced in 22 of 29 comparisons (76%).	High ⊕⊕⊕⊕
		Is there a difference how VEGF-A levels are affected by laser and anti-VEGF treatment?	Anti-VEGF: 15 Laser: 5	Anti-VEGF: 287 Laser: 154	Significantly higher reduction after anti-VEGF treatment, *P* = 0.05 and *P* < 0.01 excluding high risk of bias and outlier Anti-VEGF: 0.29 [0.21–0.40] laser: 0.55 [0.32–0.97] Without high risk of bias and outlier: Anti-VEGF: 0.31 [0.25–0.38] laser: 0.77 [0.61–0.97]	+1 large magnitude of effect +1 based on the consistent direction even if including an outlier in the results for laser treatment.	High ⊕⊕⊕⊕
		Is there a difference how VEGF-A levels are affected in serum and plasma?	Serum: 13 Plasma: 5	Serum: 342 Plasma: 99	Significantly higher reduction in serum *P* < 0.01 Serum: 0.27 [0.20–0.36] Plasma: 0.63 [0.38–1.03]	+1 large magnitude of effect	Moderate ⊕⊕⊕◯
		Is there a difference how VEGF-A levels are affected by different anti-VEGF drugs?	Bevacizumab: 5 Ranibizumab: 5 Conbercept: 3 Aflibercept: 1	Bevacizumab: 47 Ranibizumab: 109 Conbercept: 102 Aflibercept: 29	No significant difference between anti-VEGF drugs, *P* = 0.38 Bevacizumab: 0.25 [0.19–0.32] Ranibizumab: 0.28 [0.13–0.61] Conbercept: 0.39 [0.24–0.63] Aflibercept: 0.31 [0.19–0.52]	−1 based on the high-rated risk of bias and when excluding those the number of available VEGF-A measurements limited the comparisons and excluded aflibercept.	Very low ⊕◯◯◯
First month after treatment	1. High risk of bias for ≥1 domains in 8 of 21 estimated studies; however, excluding those did not change the results 2. High heterogeneity since the time factor introduce inconsistency results in −1 3. No indirectness of evidence 4. Low to moderate Imprecision 5. Moderate risk of publication bias 6. No dose-response gradient	Does ROP treatment affect VEGF-A levels?	22	976	Reduced VEGF-A levels after treatment 0.35 [0.28–0.43]	+1 large magnitude of effect +1 based on common effect. VEGF levels after treatment were significantly reduced in 48 of 68 comparisons (71%).	Moderate ⊕⊕⊕◯
		Is there a difference in how VEGF-A levels are affected by anti-VEGF treatment and laser?	Anti-VEGF: 20 Laser: 6	Anti-VEGF: 736 Laser: 239	Significantly higher reduction after treatment with anti-VEGF *P* = 0.02 Anti-VEGF: 0.32 [0.25–0.41] Laser: 0.56 [0.38–0.83]	+1 large magnitude of effect	Low ⊕⊕◯◯
		Is there a difference in how VEGF-A levels are affected in serum and plasma?	Serum: 14 Plasma: 8	Serum: 535 Plasma: 441	Significantly higher reduction in serum *P* < 0.01 Serum: 0.28 [0.21–0.36] Plasma: 0.54 [0.40–0.73]	+1 large magnitude of effect	Low ⊕⊕◯◯
		Is there a difference in how VEGF-A levels are affected by different anti-VEGF drugs?	Bevacizumab: 9 Ranibizumab: 7 Conbercept: 3 Aflibercept: 2	Bevacizumab: 265 Ranibizumab: 261 Conbercept: 150 Aflibercept: 60	A significant difference in the reduction of VEGF-A among anti-VEGF drugs, *P* < 0.01 Bevacizumab: 0.19 [0.15–0.25] Ranibizumab: 0.53 [0.34–0.82] Conbercept: 0.53 [0.34–0.84] Aflibercept: 0.28 [0.18–0.45]		Very low ⊕◯◯◯
VEGF as a biomarker for ROP	1. Differences in study designs and definitions of ROP and controls result in −1 2. High risk of bias for ≥1 domains in 9 of 30 estimated studies; however, excluding those did not substantially change the results 3. Moderate to high heterogeneity 4. No indirectness of evidence 5. Moderate Imprecision 6. Moderate risk of publication bias 7. No dose-response gradient	Are postnatal VEGF-A levels a good predictive biomarker for ROP?	19	ROP: 902 Control: 1273	VEGF-A levels showed no difference between ROP and control 0.92 [0.80–1.06] None of the included postnatal weeks showed a significant change in VEGF-A levels.	+1 based on that results showed both higher and lower levels of VEGF -A for a group with ROP compared with a group with no-ROP.	Low ⊕⊕◯◯
		Are VEGF-A levels at a postmenstrual time scale a good biomarker for ROP?	11	ROP: 1345 Control: 1865	VEGF-A levels showed no difference between ROP and control 0.98 [0.87–1.10] Only postmenstrual week 29 showed reduced VEGF-A levels for infants with ROP, but subsetting studies evaluating severe ROP did not show any difference in VEGF-A levels.	−1 since there is a risk that the ROP group can include infants both treated and untreated for ROP at later postmenstrual weeks.	Very low ⊕◯◯◯

CI = confidence interval; GRADE = Grading of Recommendations, Assessment, Development, and Evaluation; ROM = ratio of means; ROP = retinopathy of prematurity.

∗ Reported follow-up samples for treatment.

## Discussion

This meta-analysis incorporated data from 24 studies to assess circulating VEGF-A levels after ROP treatment. The key finding was a significant reduction in VEGF-A levels after ROP treatment, with a more substantial decrease observed with anti-VEGF drugs compared with laser therapy alone. Notably, among the anti-VEGF drugs, bevacizumab treatment resulted in the most pronounced reduction in VEGF-A. Furthermore, systemic VEGF-A levels were found to be unreliable indicators of ROP development in the 28 publications included in the meta-analysis.

### Factors Impacting Measured VEGF-A Concentrations

Several variables can impact VEGF-A measurements, including the sampling system and preanalytical conditions, such as time and temperature.^6^ The specificity of assays for VEGF variants and isoforms may also contribute to variations. Reviewed publications employed diverse assays, preventing a meta-analysis specifically on the analytical method because of the limited number of studies using a similar approach. Given that platelets release VEGF-A during coagulation, a distinct difference in VEGF-A concentration exists between serum and plasma samples.^6^ Notably, serum samples generally exhibited a clearer reduction in VEGF-A levels after ROP treatment compared with plasma samples; however, both systems demonstrated a similar trend of reduced levels.

Heterogeneity in results reflects substantial intra- and interindividual variation, highlighting the need to assess intraindividual variation before and after ROP treatment. Considering of denser data, potentially involving a larger number of patients, is crucial. Additionally, a more extended monitoring period is necessary to acquire further evidence regarding the long-term effects of treatment on systemic VEGF levels. Among the 14 publications that assessed VEGF-A concerning ROP-treatment, a statistic test for repeated sampling was reported. However, a few publications omitted a statistical comparison for repeated sampling or failed to disclose the statistical test used. Some publications did not statistically report a comparison of VEGF-A levels before and after treatment.

### ROP Treatment Influences VEGF-A Levels

Both anti-VEGF and laser treatments showed reduced VEGF-A levels, with the former demonstrating a more pronounced effect, particularly during the initial posttreatment week, where all anti-VEGF drugs exhibited a similar decrease in VEGF-A levels. Among the anti-VEGF drugs, bevacizumab and aflibercept displayed the most substantial reduction in VEGF-A levels within the first month after treatment, while conbercept and ranibizumab exhibited comparable outcomes.^72^ The duration of VEGF-A reduction varied after treatment. In the case of laser treatment, the reported levels after the first week were limited. Ranibizumab and conbercept demonstrated a pattern of normalized VEGF-A levels in the first week or weeks posttreatment. However, bevacizumab and aflibercept exhibited significantly reduced levels lasting up to 8 weeks after treatment, and potentially beyond. This discrepancy may be attributed to distinct antibody properties, including affinity for VEGF,^84^ vitreous,^85^ and systemic^86^ half-lives of the drug, as well as compartment distribution.^87^ However, it is essential to acknowledge that interference in the analytical method might also contribute to these variations.^88^ However, it is important to investigate the normal development of VEGF for infants not treated for ROP during a similar time period further.

### VEGF-A Levels Do Not Predict ROP Development

Our study did not find a link between systemic VEGF-A levels and the development of ROP. Discrepancies were evident when comparing cases of ROP with controls, with influential factors including the timing of VEGF-A measurements, the sample system employed, and the analytical method used. Moreover, inconsistencies in the definitions of ROP and control groups across publications likely impacted the results. However, the findings did not reveal any consistent trend indicating higher or lower VEGF levels as a predictive factor for ROP.

In terms of the association between VEGF-A levels and the pathology of ROP, no definitive conclusions can be drawn regarding whether VEGF-A levels are altered during phase II ROP. During later postmenstrual weeks, it remains uncertain whether the ROP group comprises infants who received treatment for ROP and those who did not. As demonstrated in this study, the inclusion of infants treated for ROP can affect circulating VEGF-A levels. Some publications investigated the relationship between VEGF-A in the vitreous or aqueous humor and ROP, reporting higher VEGF-A levels in severe stages of ROP compared with controls or a group with vascularly inactive ROP.^89^, ^90^, ^91^ Conversely, 1 study measuring VEGF-A in tear fluid reported lower levels in infants with ROP.^92^ Given the close association between ROP pathology and VEGF-A levels, particularly the elevation of VEGF-A during the second phase of ROP when pathological vessel growth occurs,^93^ it is plausible that systemic levels of VEGF-A also increase during this phase.

### Risk of Bias in Included Publications

Concerning the quality of the included publications, a potential issue arises as they were not primarily designed to measure VEGF levels. This lack of focusing on VEGF measurement may explain the absence of information regarding the performance of analytical methods that influence both the index test and the flow and timing domains. Nonetheless, three-quarters of the studies demonstrated a low risk of bias related to patient selection. Most studies utilized commercially available analytical methods and references, and ROP diagnosis was predominantly reported to align with international standards.^94^

### Publication Bias

When examining VEGF-A levels after treatment for ROP, a reduction in the number of individuals and samples over time was observed, introducing a heightened risk of publication bias in subsequent follow-up results. This decline may be attributed to ethical considerations and the judicious use of samples. Egger’s test identified a potential publication bias in studies assessing laser treatment and the utilization of VEGF-A as a biomarker for predicting ROP. Contrarily, an alternative method recommended for meta-analysis, employing standardized mean difference as proposed by Pustejovsky and Rogers,^95^ yielded opposing results, a finding consistent with the observation of Lin Lefeng.^96^ Furthermore, upon exclusion of a study identified as an outlier following laser treatment, Egger’s test no longer indicated publication bias. Upon omitting publications deemed to possess a high risk of bias in the assessment of VEGF-A as a biomarker, an analysis on the first day of life was precluded because of the limited number of eligible publications.

### Strengths and Limitations

This study, adhering to Preferred Reporting Items for Systematic Reviews and Meta-Analyses guidelines, synthesized a comprehensive dataset not previously summarized. The study protocol was meticulously designed, incorporating a broad search strategy. Additional publications were identified through reference, citation, and gray literature searches; however, the primary studies were mostly captured by the search strategy. Exclusion of publications with a high risk of bias in ≥1 domains refined the heterogeneity and strengthened the evidence of posttreatment effects on VEGF levels within the first week and month.

Nevertheless, challenges persisted, including variations in study design that limit the number of available publications reporting VEGF-A levels with similar subgroup properties, and absence of well-suited protocols and tools for systematic review and meta-analysis of longitudinal data on biomarker levels. Although the Quality Assessment of Diagnostic Accuracy Studies-2 tool was developed for evaluating diagnostic tests, its applicability to studies assessing biomarker concentrations including omics-based publications has been debated.^97^ However, no superior alternative has been identified to date, as VEGF-A concentrations do not fit the omics data category. Consequently, a quality assessment was conducted, incorporating considerations related to the preanalytical and analytical method performance within the index-test domain.

The results exhibited not only high heterogeneity, likely attributed to sampling time, as indicated in subgroup analysis, but also other factors influencing VEGF-A concentrations. Inclusion of longitudinal data in the meta-analysis was suboptimal, prompting the summarization of time periods to compare data and mitigate the influence of time when evaluating subgroups. Although excluding studies with a high risk of bias reduced heterogeneity, it significantly limited available data, particularly for investigating VEGF-A levels in relation to different anti-VEGF drugs. However, using an alternative strategy to summarize data according to the synthesis without meta-analysis in systematic reviews guidelines,^28^ only requiring *P* values and the direction of reported effects, indicated similar results as the meta-analysis.

There is a possibility of overlooking reports on VEGF-A and ROP. The search strategy utilized 2 databases, PubMed and Scopus, while other databases such as Embase and Web of Science are available in the field. A gray literature search, comprising the first 100 hits in Google Scholar, was conducted; however, references and citations of the included publications were also scrutinized.

### Future Directions

A meta-analysis of recurrence after ROP treatment indicated a higher retreatment rate after anti-VEGF compared with laser, especially for ranibizumab.^8^ The pharmacodynamics after anti-VEGF treatment of ROP have been extensively investigated.^31^^,^^38^^,^^46^^,^^98^ Results from these studies suggest that all anti-VEGF drugs persist in circulation after treatment, but their half-life in circulation varies between drugs. Garcia-Quintanilla et al ^99^ compiled available pharmacokinetic preclinical and clinical studies of anti-VEGF antibodies related to age-related macular degeneration. They identified factors potentially affecting anti-VEGF pharmacokinetics, including varying molecular properties of antibodies, distribution-diffusion in the vitreous humor, drug elimination from the vitreous humor, surgical ocular procedures, and the analytical methods used in pharmacokinetic studies.

The factors identified as affecting circulating VEGF-A levels likely influenced free VEGF-A in pharmacodynamic studies of anti-VEGF after ROP treatment. An alternative analytical method to specifically measure circulating VEGF-A and the antibody comprising the anti-VEGF drug, whether free or in complex with VEGF-A, may be necessary, ideally to eliminate uncertainties associated with antibody use.

When planning future studies to evaluate circulating VEGF-A levels, it is crucial to consider the blood sampling system and preanalytical handling procedures, as these can impact measured VEGF-A levels.^6^ The analytical method must detect VEGF-A levels within a stable concentration range. Additionally, potential interference with the analytical method using anti-VEGF drugs, as reported by Sumner et al,^88^ should be considered and further evaluated. In designing future studies on VEGF-A as a biomarker for ROP pathophysiology, the timing of ROP diagnosis and treatment needs to be considered. The limited blood volume in preterm infants is another critical aspect when planning prospective collection of blood samples, explaining why adults can be used for larger studies later validated by sparse data from preterm infants.

For future systematic reviews with meta-analyses of biomarker concentrations, the development of relevant optimized tools and recommendations for risk-of-bias evaluation and estimation of publication bias are needed.^96^ The odds ratio and adjusted odds ratio are preferable alternatives when conducting a meta-analysis to assess the potential of VEGF-A as a predictive blood-based biomarker, or data sharing of deidentified raw data, time points, and ROP outcomes.

The administration of anti-VEGF for treatment of ROP significantly diminishes circulating VEGF-A levels within the initial week, with serum samples providing a more distinct representation than plasma samples. Nevertheless, the inherent variability observed in the results underscores the imperative for improved analytical methods, recognizing the considerable individual variation in VEGF-A levels. Notably, neither serum nor plasma concentrations of VEGF-A correlated with the development of ROP. Future research aimed at monitoring the suppression of VEGF-A levels after ROP treatment should address existing knowledge gaps, particularly concerning preanalytical factors such as the optimal sample system.

## Declaration of Generative AI and AI-Assisted Technologies in the Writing Process

During the preparation of this work, the author(s) used Google Translate in order to translate from Swedish to English. In addition, this article was edited by Elsevier Language Editing Service. After using this tool/service, the author(s) reviewed and edited the content as needed and took(s) full responsibility for the content of the publication.
