# Supplementary material for: Circulating VEGF-A Levels in Relation to Retinopathy of Prematurity and Treatment Effects: A Systematic Review and Meta-Analysis
Source: Ophthalmol Sci. 2024 May 7;4(6):100548. doi: 10.1016/j.xops.2024.100548 (PMC11342886; doi:10.1016/j.xops.2024.100548)
Supplement: Supplement Appendix 3 [file mmc1.pdf]

Supplement Appendix 3

Circulating VEGF-A levels in relation to retinopathy of prematurity and treatment effects: A systematic review and meta-analysis

Short title- VEGF levels in ROP

Précis: Systemic VEGF levels were reduced after treatment of retinopathy of prematurity. Serum shows a more pronounced change in VEGF levels than plasma after the intraocular injection of anti-VEGF.

Ulrika Sjöbom[1,2 *], Tove Hellqvist[2], Jhangir Humayun[1], Anders K. Nilsson[2], Hanna Gyllensten[1], Ann Hellström[2], Chatarina Löfqvist[1,2]

List of content

Supplement Appendix 3 1

Table S1. Identified but excluded publications. 4

Subgroup analysis treatment – Time and treatment type 6

Figure S5 VEGF levels the first week after ROP treatment: laser, vs. anti-VEGF 6

Figure S6 VEGF levels the first month after ROP treatment: laser, vs. anti-VEGF 7

Subgroup analysis treatment- Time and type of anti-VEGF 8

Figure S7 VEGF-levels the first week after anti-VEGF treatment, comparing types of anti-VEGF 8

Figure S8 VEGF-levels the first month after anti-VEGF treatment, comparing types of anti-VEGF 9

Subgroup analysis treatment- Laser treatment and Time, per week 10

Figure S10 VEGF-levels after laser treatment per week 10

Subgroup analysis treatment- Anti-VEGF treatment, Time and type, per week 11

Figure S11 VEGF-levels after anti-VEGF treatment per week 11

Figure S12 VEGF-levels after treatment with Bevacizumab per week 12

Figure S13 VEGF-levels after treatment with Ranibizumab per week 13

Figure S14 VEGF-levels after treatment with Conbercept per week 13

Figure S15 VEGF-levels after treatment with Aflibercept per week 14

Subgroup analysis treatment- Time and sample system 15

Figure S16 The change of VEGF levels per sample system the first week after treatment 15

Figure S17 The change of VEGF levels per sample system the first month after treatment 16

Subgroup analysis treatment- Time, sample system and treatment type 17

Figure S19 The change of VEGF levels per sample system the first week after treatment with Laser 17

Figure S20 The change of VEGF levels per sample system the first month after treatment with Laser 17

Figure S21 The change of VEGF levels per sample system the first week after treatment with Bevacizumab 18

Figure S22 The change of VEGF levels per sample system the first month after treatment with Bevacizumab 18

Figure S23 The change of VEGF levels per sample system the first week after treatment with Ranibizumab 19

Figure S24 The change of VEGF levels per sample system the first month after treatment with Ranibizumab 19

Funnel plot- Treatment 20

Figure S25 Funnel plots for publications investigating VEGF levels one week after treatment of ROP 20

Figure S26 Funnel plots for publications investigating VEGF levels one week after laser treatment of ROP 21

Figure S27 Funnel plots for publications investigating VEGF levels one week after treatment with Bevacizumab for ROP 22

Figure S28 Funnel plots for publications investigating VEGF levels one week after treatment with Ranibizumab for ROP 23

Subgroup analysis ROP as a biomarker for ROP- PNA and time 24

Figure S30 VEGF levels as a biomarker in relation to ROP for postnatal ages 24

Subgroup analysis ROP as a biomarker for ROP- PNA, time and ROP severity 25

Figure S32 VEGF levels as a biomarker in relation to severe ROP for postnatal ages 25

Subgroup analysis ROP as a biomarker for ROP- PNA, sample system and time 26

Figure S32 VEGF levels in serum and plasma at birth as biomarker in relation to ROP 26
Subgroup analysis ROP as a biomarker for ROP- PMA and time........ 27
Figure S34 VEGF levels as a biomarker in relation to ROP for postmenstrual ages 28
Subgroup analysis ROP as a biomarker for ROP- PMA, time and ROP severity 29
SFigure 35 VEGF levels as a biomarker in relation to severe ROP for postmenstrual ages........ 30
Funnel plot VEGF as a biomarker for ROP 31
Figure S36 Funnel plots for publications investigating VEGF levels the first postnatal week after birth and comparing levels between a group with ROP and a control. 31

Table S1. Identified but excluded publications.

Reference	Reason for exclusions
Fidler M, Fleck BW, Stahl A, Marlow N, Chastain JE, Li J, Lepore D, Reynolds JD, Chiang MF, Fielder AR; RAINBOW study group†. Ranibizumab Population Pharmacokinetics and Free VEGF Pharmacodynamics in Preterm Infants With Retinopathy of Prematurity in the RAINBOW Trial. Transl Vis Sci Technol. 2020 Jul 29;9(8):43. doi: 10.1167/tvst.9.8.43.	The publication uses the same cohort as: Stahl A, Lepore D, Fielder A, Fleck B, Reynolds JD, Chiang MF, et al. Ranibizumab versus laser therapy for the treatment of very low birthweight infants with retinopathy of prematurity (RAINBOW): an open-label randomised controlled trial. The Lancet. 2019;394(10208):1551-9.
Filippi L, Cavallaro G, Bagnoli P, Dal Monte M, Fiorini P, Donzelli G, Tinelli F, Araimo G, Cristofori G, la Marca G, Della Bona ML, La Torre A, Fortunato P, Furlanetto S, Osnaghi S, Mosca F. Oral propranolol for retinopathy of prematurity: risks, safety concerns, and perspectives. J Pediatr. 2013 Dec;163(6):1570-1577.e6.	VEGF concentration in relation to propanolol as prevention of ROP, not treatment and does not compare ROP and control
Ma IT, McConaghy S, Namachivayam K, Halloran BA, Kurundkar AR, MohanKumar K, Maheshwari A, Ohls RK. VEGF mRNA and protein concentrations in the developing human eye. Pediatr Res. 2015 Apr;77(4):500-5. doi: 10.1038/pr.2015.15. Epub 2015 Jan 14.	No VEGF-A levels in relation to ROP
Sang-Joon Lee, Soo-Yong Kim, Byeng chul Yoo, Hyun Woong Kim, Young Ho Kim; Plasma Level Of Vascular Endothelial Growth Factor In Retinopathy Of Prematurity After Intravitreal Injection Of Bevacizumab. *Invest. Ophthalmol. Vis. Sci.* 2011;52(14):3165.	The same cohort as: Hong YR, Kim YH, Kim SY, Nam GY, Cheon HJ, Lee SJ. PLASMA CONCENTRATIONS OF VASCULAR ENDOTHELIAL GROWTH FACTOR IN RETINOPATHY OF PREMATURITY AFTER INTRAVITREAL BEVACIZUMAB INJECTION. Retina. 2015;35(9):1772-7.
Lopez Yomayuza CC, Preissner KT, Lorenz B, Stieger K. Optimizing Measurement of Vascular Endothelial Growth Factor in Small Blood Samples of Premature Infants. Sci Rep. 2019 May 1;9(1):6744.	No VEGF-A levels in relation to ROP
Krzysztof Zięba, Elżbieta Kasprzak, Dorota Tomaszkiewicz-Mondry, Ada Pomykalska, Małgorzata Bierczyńska-Kicińska, Janusz Gadzinowski, Porównanie stężeń IGF-1 i VEGF w surowicy krwi z tętnicy pępowinowej u noworodków urodzonych przedwcześnie, zagrożonych wystąpieniem retinopatii i u noworodków urodzonych o czasie, Pediatria Polska,Volume 82, Issues 5–6, 2007, Pages 437-440, ISSN 0031-3939,	No VEGF-A levels in relation to ROP
Machalińska A, Modrzejewska M, Dziedziejko V, Kotowski M, Safranow K, Herbowska A, Karczewicz D. Ocena stezenia VEGF i IGF-1 we krwi obwodowej wcześniaków--próba korelacji z rozwojem retinopatii wcześniaczej, implikacje kliniczne [Evaluation of VEGF and IGF-1 plasma levels in preterm infants--potential correlation with retinopathy of prematurity, clinical implications]. Klin Oczna. 2009;111(10-12):302-6.	Uses the same cohort as Machalinska et al. but with less information: Machalinska A, Modrzejewska M, Kotowski M, Dziedziejko V, Kucia M, Kawa M, et al. Circulating stem cell populations in preterm infants: implications for the development of retinopathy of prematurity. Archives of ophthalmology (Chicago, Ill : 1960). 2010;128(10):1311-9
Phalak D, Rani PK, Balakrishnan D, Jalali S. Central retinal vein obstruction in a neonate occurring during laser photocoagulation treatment for retinopathy of prematurity. J Pediatr Ophthalmol Strabismus. 2014 Dec 1;51 Online:e72-4.	No VEGF-A concentrations were compared
Tayman, C. , Çakır, U. , Özdemir, Ö. "The value of VEGF and IGF-1 in the diagnosis retinopathy of prematurity and follow-up of response to laser therapy" . Cukurova Medical Journal 44 (2019): 745-752	Uses partly the same cohort as Cakir et al.: Cakir U, Tayman C, Yucel C, Ozdemir O. Can IL-33 and Endocan be New Markers for Retinopathy of Prematurity? Comb Chem High Throughput Screen. 2019;22(1):41-8.
Velez-Montoya R, Clapp C, Rivera JC, Garcia-Aguirre G, Morales-Cantón V, Fromow-Guerra J, Guerrero-Naranjo JL, Quiroz-Mercado H. Intraocular and systemic levels of vascular endothelial growth factor in advanced cases of	VEGF-A concentrations in relation to later surgery of advanced ROP and not ROP treatment

retinopathy of prematurity. Clin Ophthalmol. 2010 Sep 7;4:947-53.	
Wallace DK, Kraker RT, Freedman SF, Crouch ER, Bhatt AR, Hartnett ME, Yang MB, Rogers DL, Hutchinson AK, VanderVeen DK, Haider KM, Siatkowski RM, Dean TW, Beck RW, Repka MX, Smith LE, Good WV, Kong L, Cotter SA, Holmes JM; Pediatric Eye Disease Investigator Group (PEDIG). Short-term Outcomes After Very Low-Dose Intravitreous Bevacizumab for Retinopathy of Prematurity. JAMA Ophthalmol. 2020 Jun 1;138(6):698-701.	No VEGF-A concentrations compared

Subgroup analysis treatment – Time and treatment type

a)

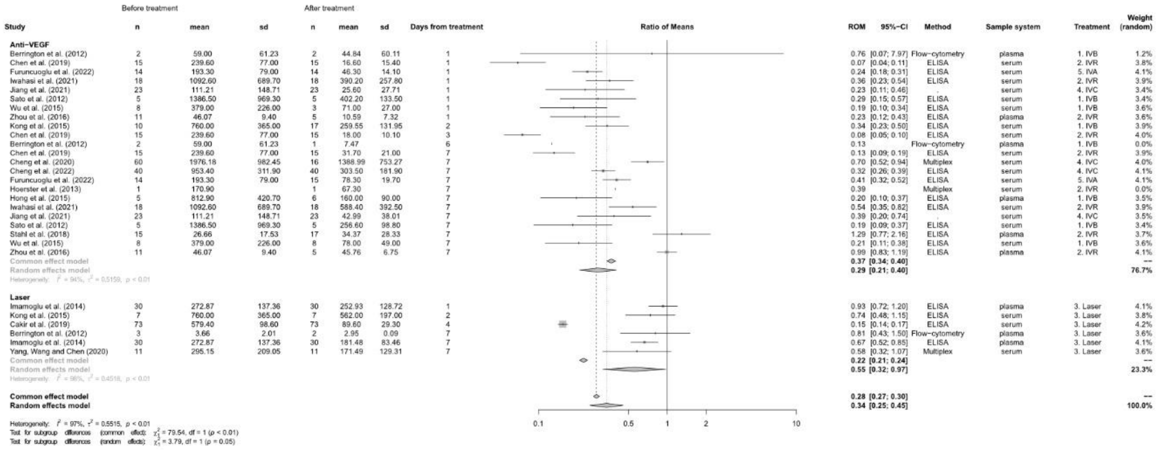

b)

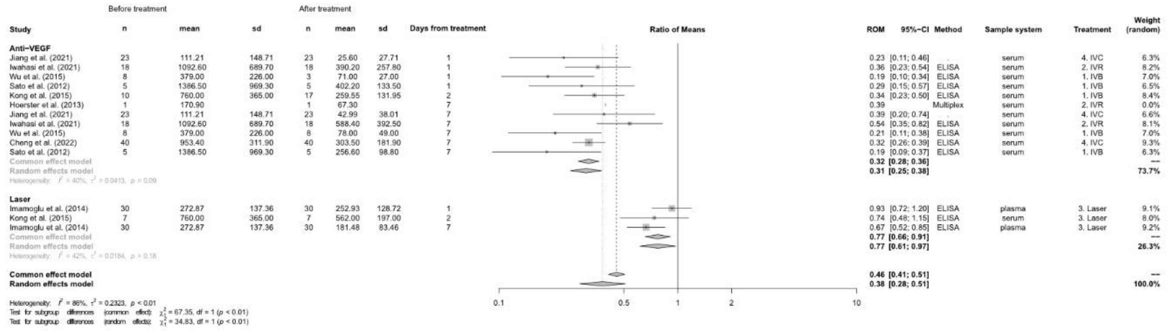

Figure S5 VEGF levels the first week after ROP treatment: laser, vs. anti-VEGF

a) Ratio of means meta-analysis performed with meta.cont in RStudio for reported vascular endothelial growth factor (VEGF) concentrations the first week after treatment for retinopathy of prematurity (ROP), ratio of VEGF concentrations calculated against before treatment including a subgroup analysis comparing anti-VEGF with laser.
b) Publications with a high risk of bias and the study by Cakir et.al. (2019) identified as an outlier excluded.

Abbreviations: CI: confidence interval, IVA: intravitreal aflibercept, IVB: intravitreal bevacizumab, IVC: intravitreal conbercept, IVR: intravitreal ranibizumab

a)

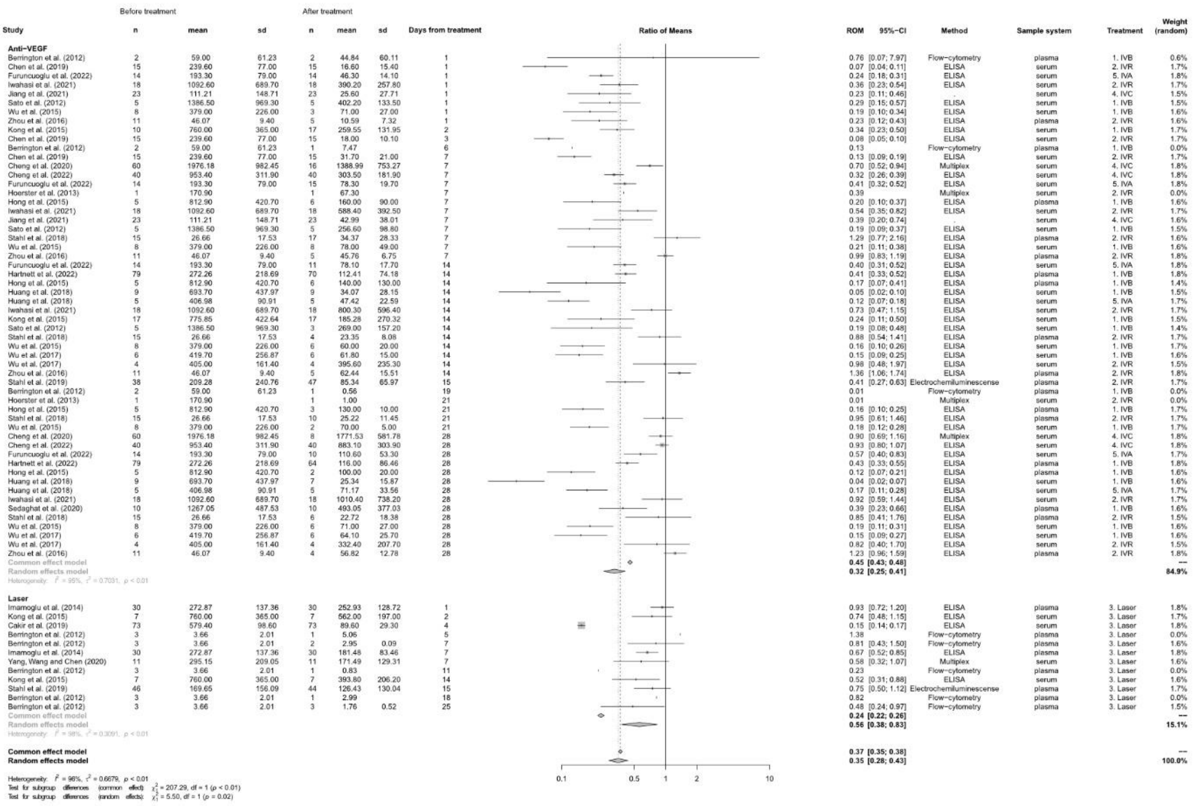

b)

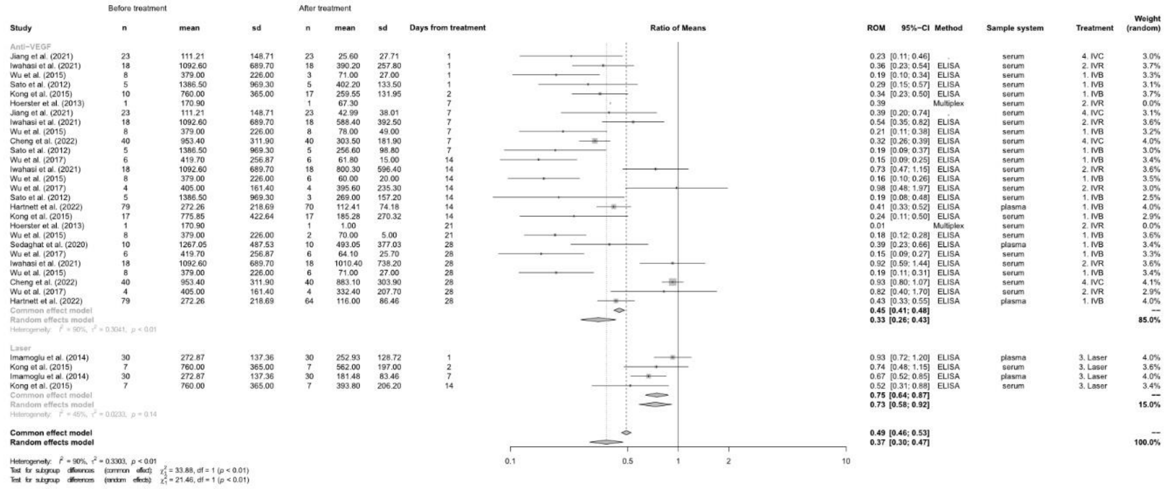

Figure S6 VEGF levels the first month after ROP treatment: laser, vs. anti-VEGF

a) Ratio of means meta-analysis performed with meta.cont in RStudio for reported vascular endothelial growth factor (VEGF) concentrations the first month after treatment for retinopathy of prematurity (ROP), ratio of VEGF concentrations calculated against before treatment including a subgroup analysis comparing anti-VEGF with laser.

b) Publications with a high risk of bias and the study by Cakir et.al. (2019) identified as an outlier excluded.

Abbreviations: CI: confidence interval, IVA: intravitreal aflibercept, IVB: intravitreal bevacizumab, IVC: intravitreal conbercept, IVR: intravitreal ranibizumab

Subgroup analysis treatment- Time and type of anti-VEGF

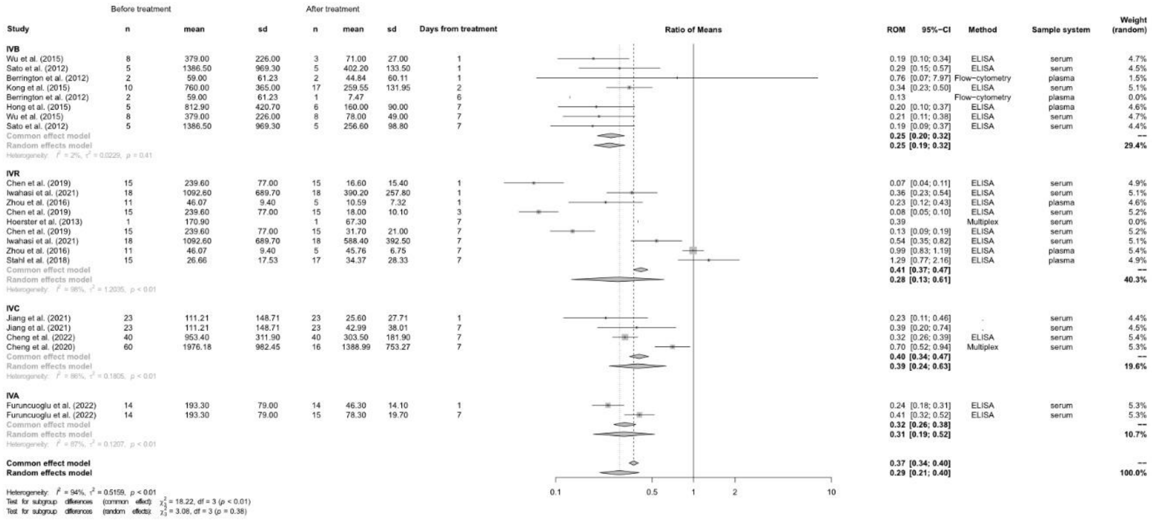

b)

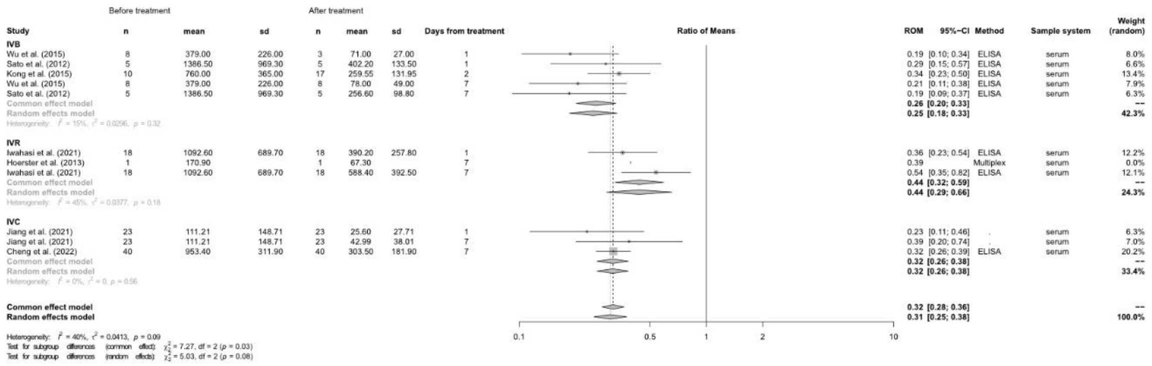

Figure S7 VEGF-levels the first week after anti-VEGF treatment, comparing types of anti-VEGF

a) Ratio of means meta-analysis performed with meta.cont in RStudio for reported vascular endothelial growth factor (VEGF) concentrations the first week after treatment for retinopathy of prematurity (ROP), ratio of VEGF concentrations calculated against before treatment including a subgroup analysis comparing different types of anti-VEGF treatments.

b) Publications with a high risk of bias excluded

Abbreviations: CI: confidence interval, IVA: intravitreal aflibercept, IVB: intravitreal bevacizumab, IVC: intravitreal conbercept, IVR: intravitreal ranibizumab

a)

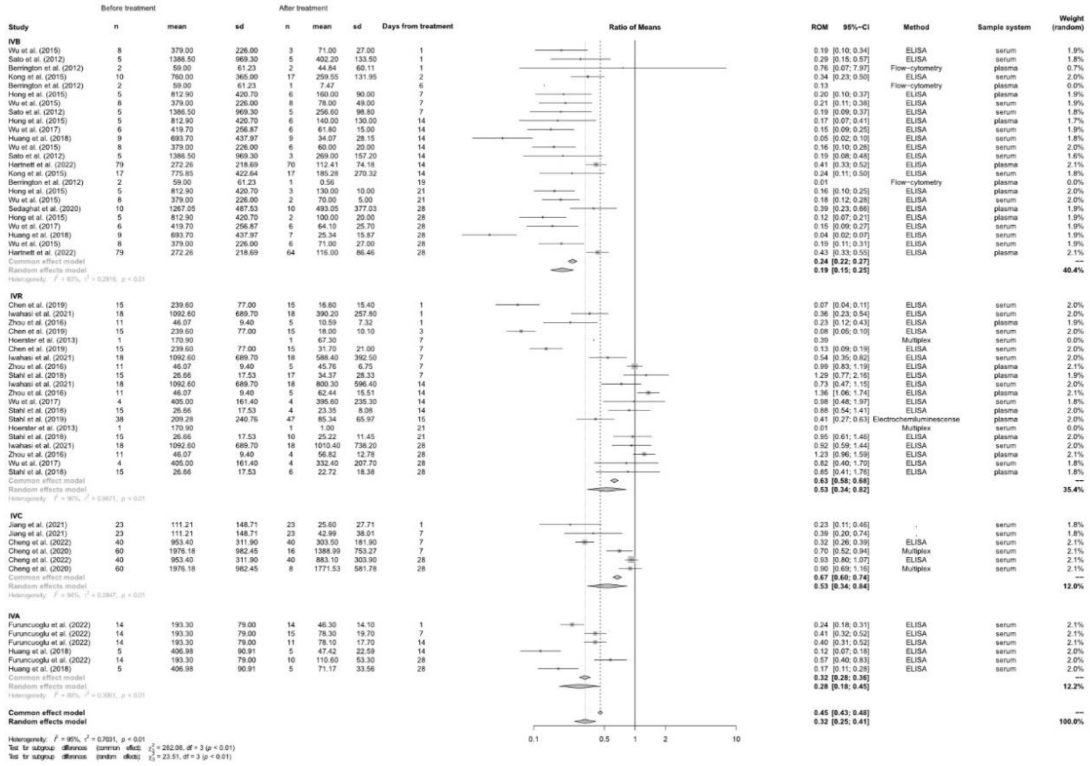

b)

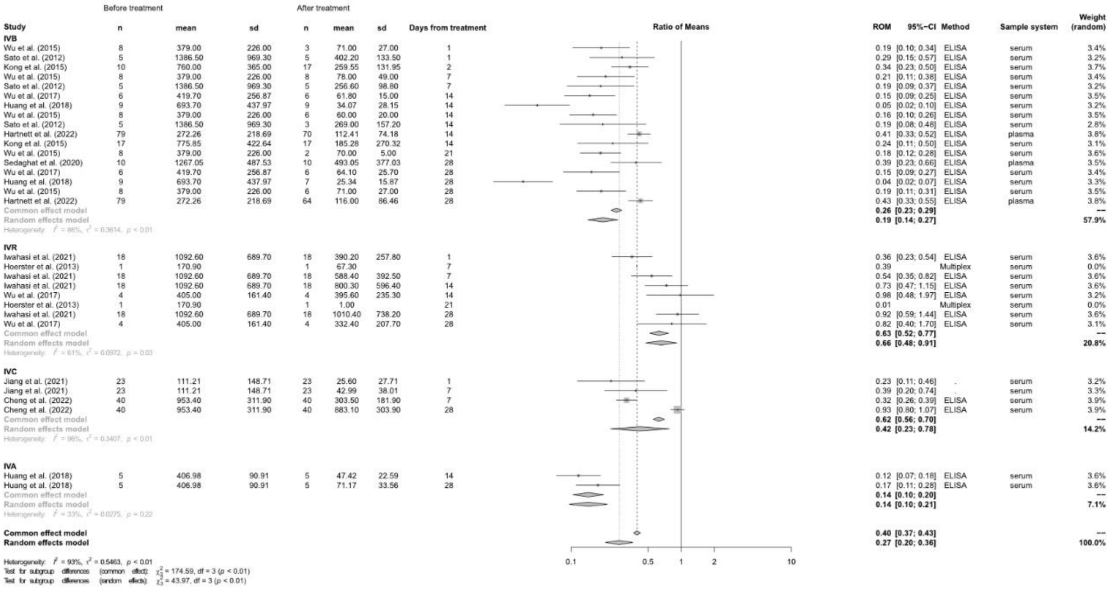

Figure S8 VEGF-levels the first month after anti-VEGF treatment, comparing types of anti-VEGF

a) Ratio of means meta-analysis performed with meta.cont in RStudio for reported vascular endothelial growth factor (VEGF) concentrations the first month after treatment for retinopathy of prematurity (ROP), ratio of VEGF concentrations calculated against before treatment including a subgroup analysis comparing different types of anti-VEGF treatments.
b) Publications with a high risk of bias excluded.

Abbreviations: CI: confidence interval, IVA: intravitreal aflibercept, IVB: intravitreal bevacizumab, IVC: intravitreal conbercept, IVR: intravitreal ranibizumab

Subgroup analysis treatment- Laser treatment and Time, per week

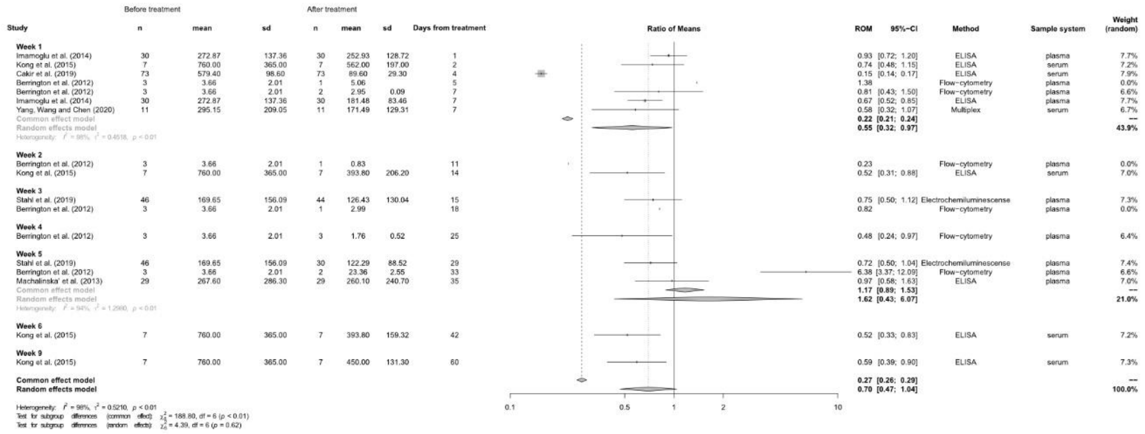

Figure S10 VEGF-levels after laser treatment per week

Ratio of means meta-analysis performed with meta.cont in RStudio for reported vascular endothelial growth factor (VEGF) concentrations after laser treatment of retinopathy of prematurity (ROP), ratio of VEGF concentrations calculated against before treatment including a subgroup analysis comparing the weeks after treatment.

Abbreviations: SD: standard deviation, CI: confidence interval

Subgroup analysis treatment- Anti-VEGF treatment, Time and type, per week

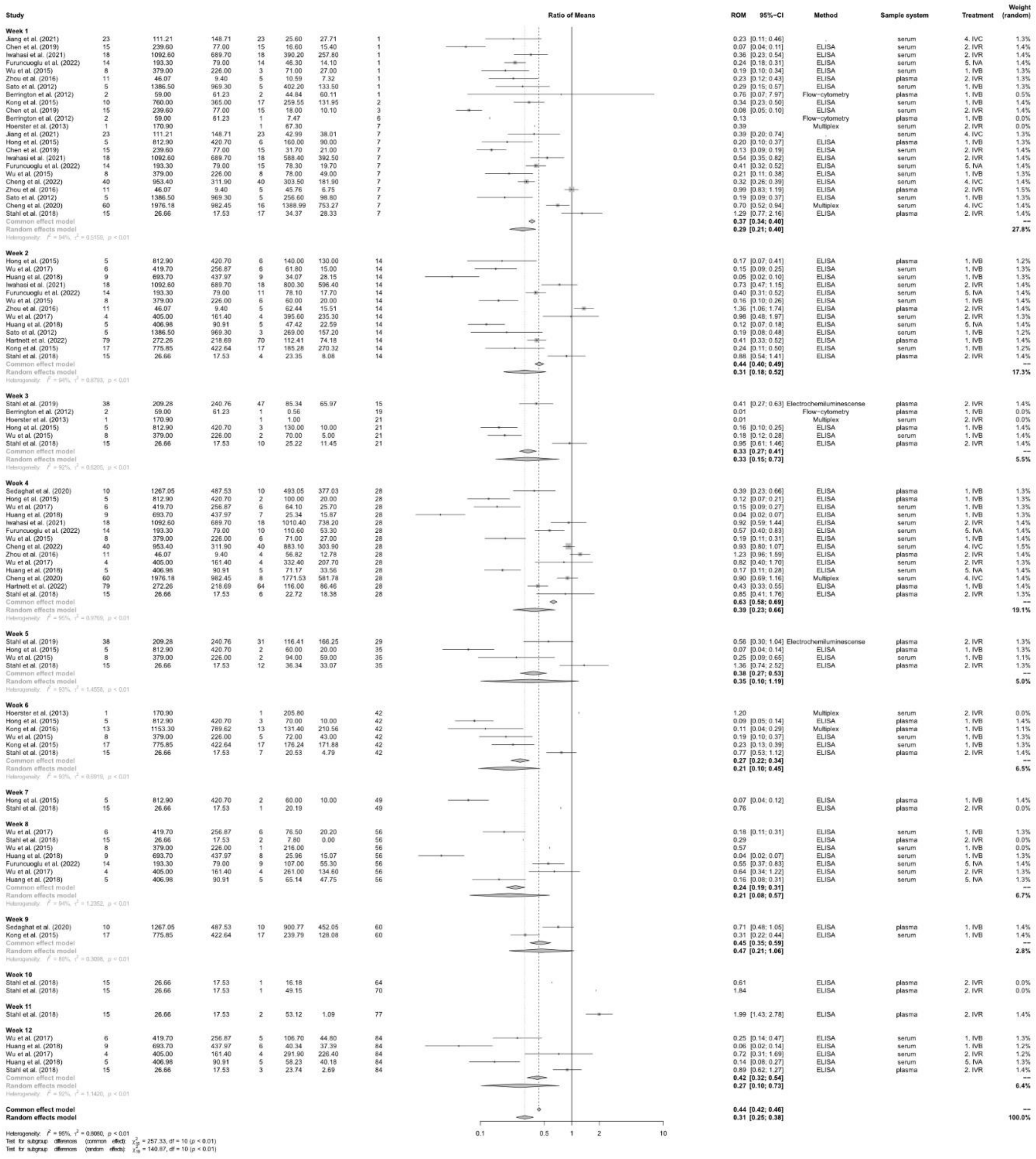

Figure S11 VEGF-levels after anti-VEGF treatment per week

Ratio of means meta-analysis performed with meta.cont in RStudio for reported vascular endothelial growth factor (VEGF) concentrations after anti-VEGF treatment of retinopathy of prematurity (ROP), ratio of VEGF concentrations calculated against before treatment including a subgroup analysis comparing the weeks after treatment.

Abbreviations: SD: standard deviation, CI: confidence interval

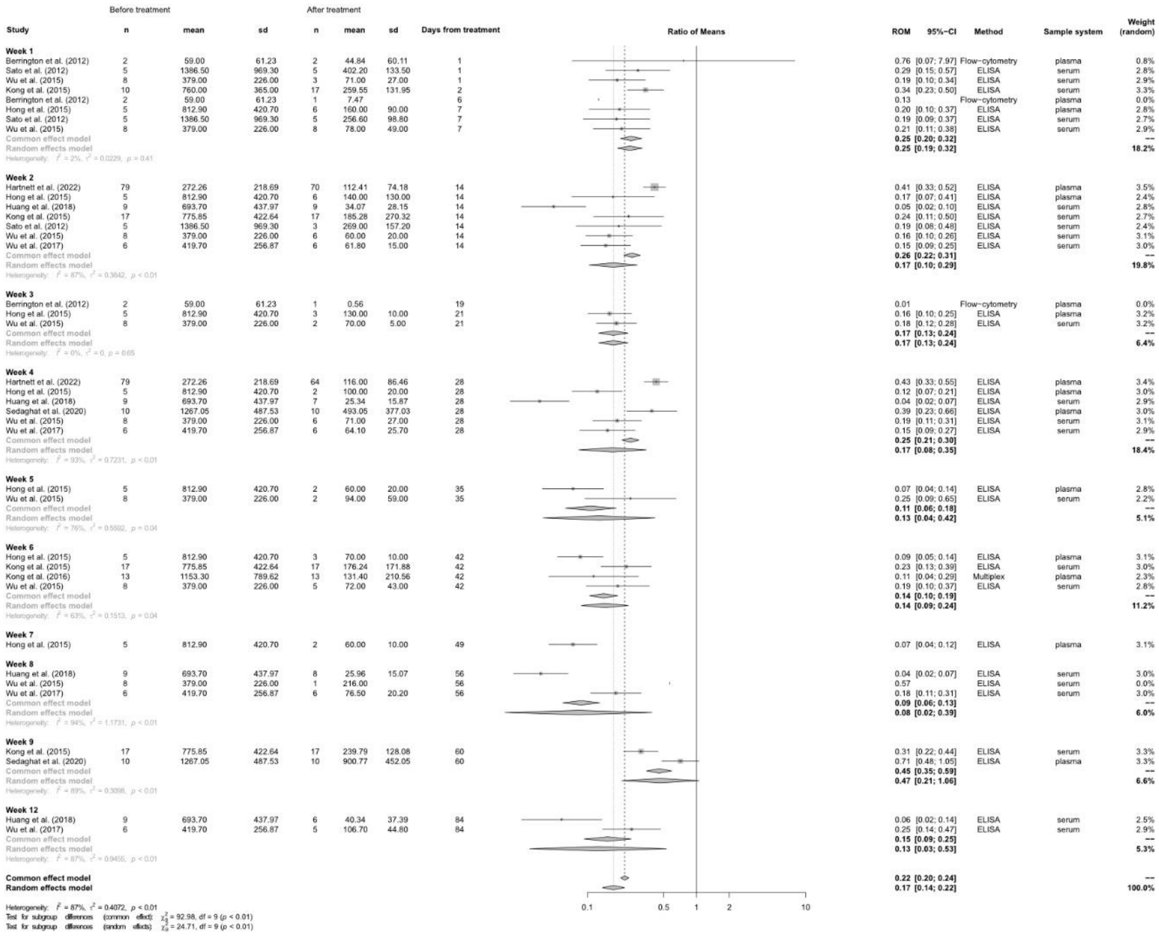

Figure S12 VEGF-levels after treatment with Bevacizumab per week

Ratio of means meta-analysis performed with meta.cont in RStudio for reported vascular endothelial growth factor (VEGF) concentrations after treatment of retinopathy of prematurity (ROP) with Bevacizumab, ratio of VEGF concentrations calculated against before treatment including a subgroup analysis comparing the weeks after treatment.

Abbreviations: SD: standard deviation, CI: confidence interval

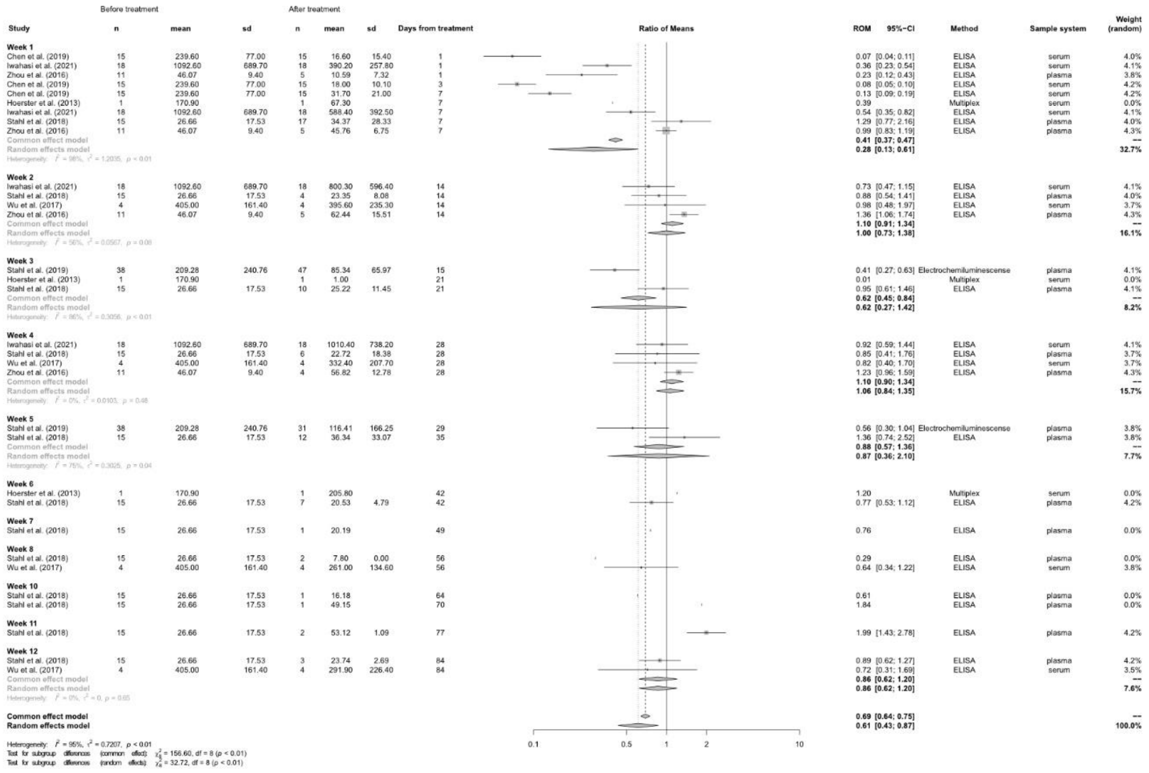

Figure S13 VEGF-levels after treatment with Ranibizumab per week

Ratio of means meta-analysis performed with meta.cont in RStudio for reported vascular endothelial growth factor (VEGF) concentrations after treatment of retinopathy of prematurity (ROP) with Ranibizumab, ratio of VEGF concentrations calculated against before treatment including a subgroup analysis comparing the weeks after treatment.

Abbreviations: SD: standard deviation, CI: confidence interval

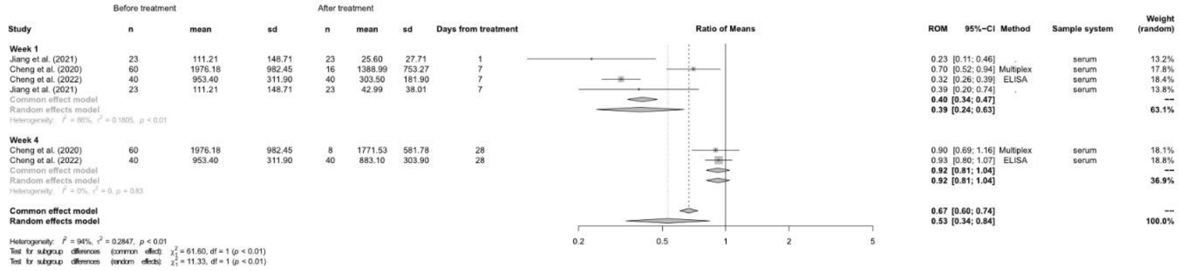

Figure S14 VEGF-levels after treatment with Conbercept per week

Ratio of means meta-analysis performed with meta.cont in RStudio for reported vascular endothelial growth factor (VEGF) concentrations after treatment of retinopathy of prematurity (ROP) with Conbercept, ratio of VEGF concentrations calculated against before treatment including a subgroup analysis comparing the weeks after treatment.

Abbreviations: SD: standard deviation, CI: confidence interval

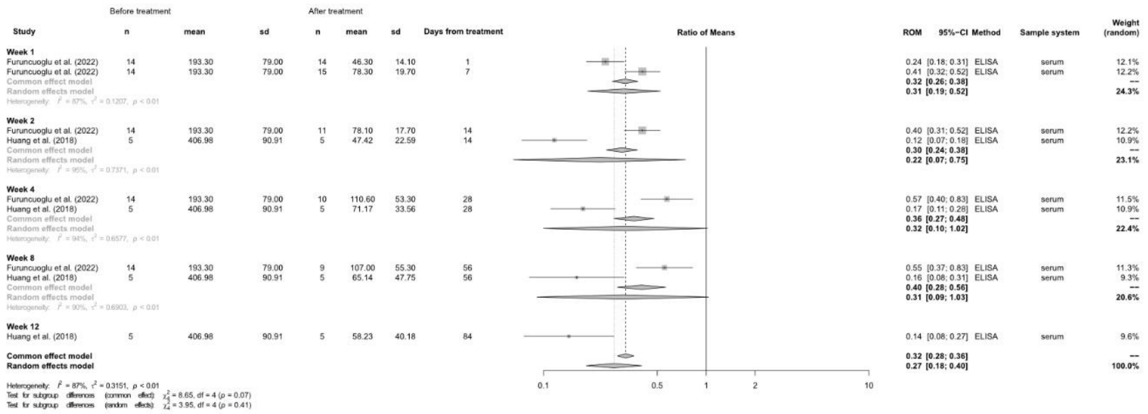

Figure S15 VEGF-levels after treatment with Aflibercept per week

Ratio of means meta-analysis performed with meta.cont in RStudio for reported vascular endothelial growth factor (VEGF) concentrations after treatment of retinopathy of prematurity (ROP) with Aflibercept, ratio of VEGF concentrations calculated against before treatment including a subgroup analysis comparing the weeks after treatment.

Abbreviations: SD: standard deviation, CI: confidence interval

Subgroup analysis treatment- Time and sample system

a)

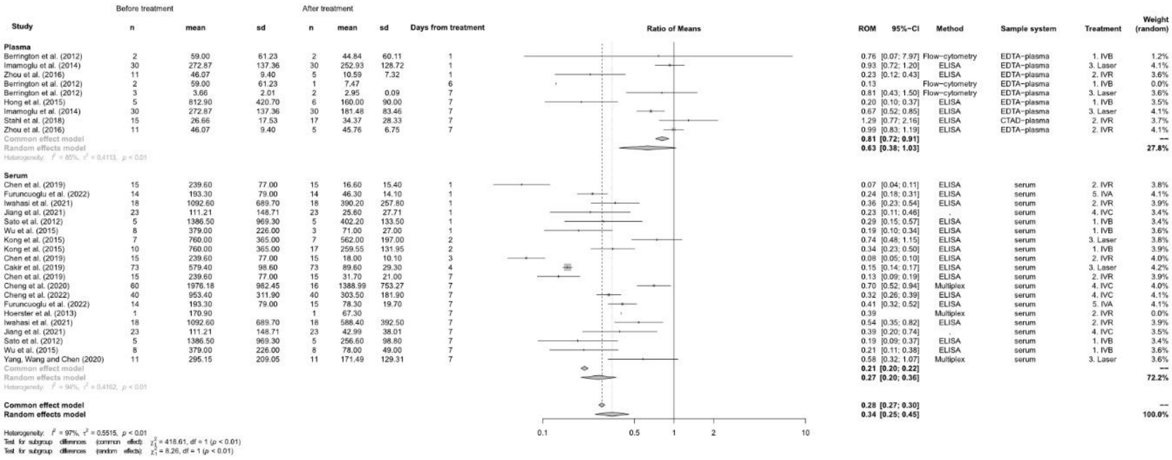

b)

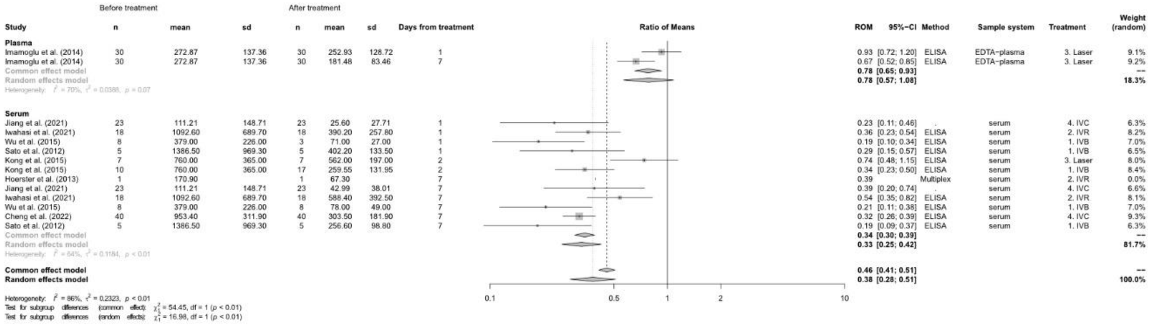

Figure S16 The change of VEGF levels per sample system the first week after treatment

a) Ratio of means meta-analysis performed with meta.cont in RStudio for reported vascular endothelial growth factor (VEGF) concentrations after treatment of retinopathy of prematurity (ROP), ratio of VEGF concentrations calculated against before treatment including a subgroup analysis comparing the sample system (serum vs. plasma).
b) Publications with a high risk of bias and the study by Cakir et.al. (2019) identified as an outlier excluded.

Abbreviations: SD: standard deviation, CI: confidence interval

a)

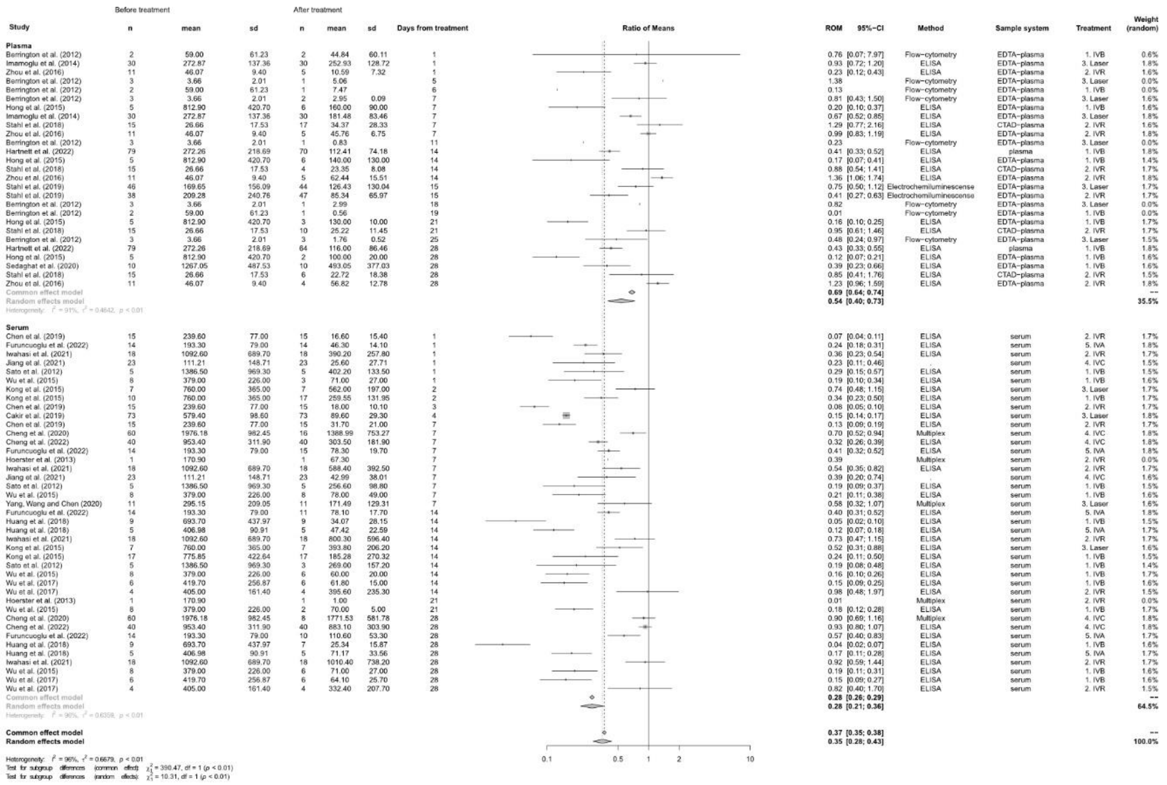

b)

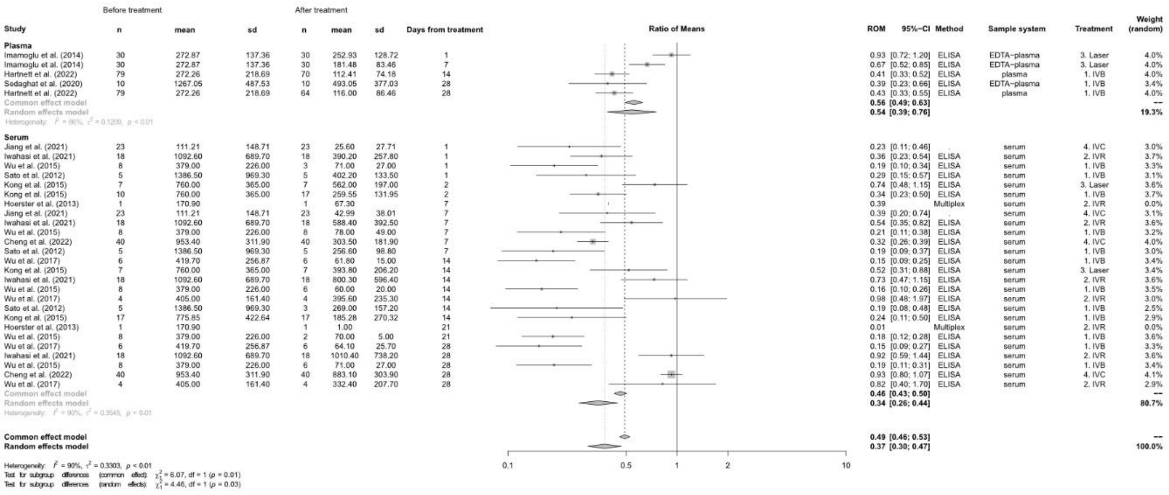

Figure S17 The change of VEGF levels per sample system the first month after treatment

a) Ratio of means meta-analysis performed with meta.cont in RStudio for reported vascular endothelial growth factor (VEGF) concentrations after treatment of retinopathy of prematurity (ROP), ratio of VEGF concentrations calculated against before treatment including a subgroup analysis comparing the sample system (serum vs. plasma).
b) Publications with a high risk of bias and the study by Cakir et.al. (2019) identified as an outlier excluded.

Abbreviations: SD: standard deviation, CI: confidence interval, IVA: intravitreal aflibercept, IVB: intravitreal bevacizumab, IVC: intravitreal conbercept, IVR: intravitreal ranibizumab

Subgroup analysis treatment- Time, sample system and treatment type

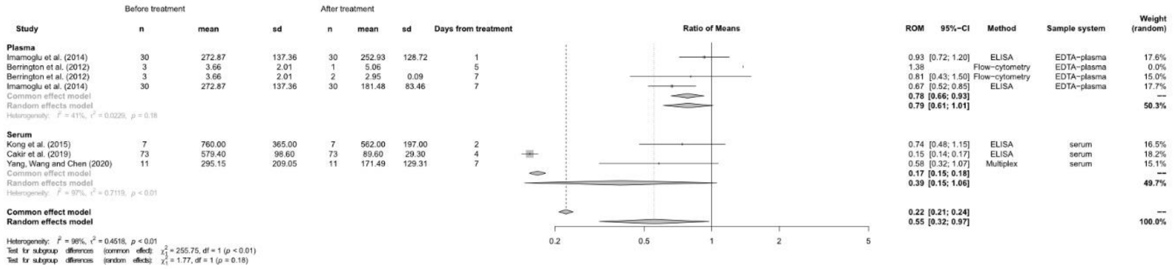

Figure S19 The change of VEGF levels per sample system the first week after treatment with Laser

Ratio of means meta-analysis performed with meta.cont in RStudio for reported vascular endothelial growth factor (VEGF) concentrations after treatment of retinopathy of prematurity (ROP) with Laser, ratio of VEGF concentrations calculated against before treatment including a subgroup analysis comparing the sample system (serum vs. plasma).

Abbreviations: SD: standard deviation, CI: confidence interval

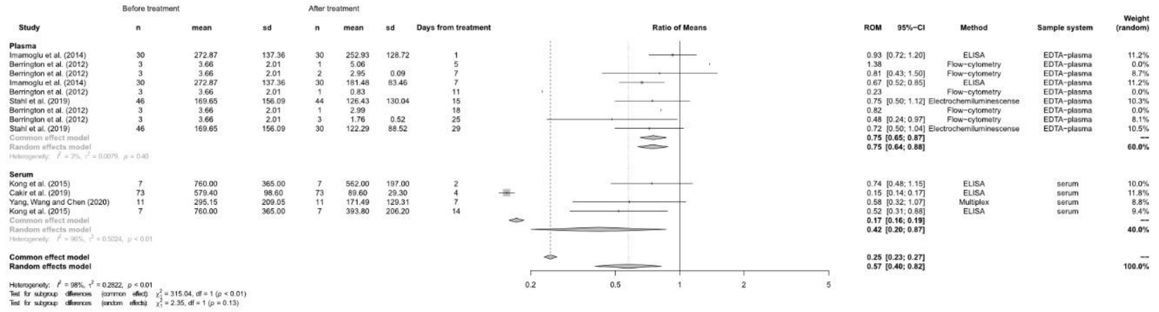

Figure S20 The change of VEGF levels per sample system the first month after treatment with Laser

Ratio of means meta-analysis performed with meta.cont in RStudio for reported vascular endothelial growth factor (VEGF) concentrations after treatment of retinopathy of prematurity (ROP) with Laser, ratio of VEGF concentrations calculated against before treatment including a subgroup analysis comparing the sample system (serum vs. plasma).

Abbreviations: SD: standard deviation, CI: confidence interval

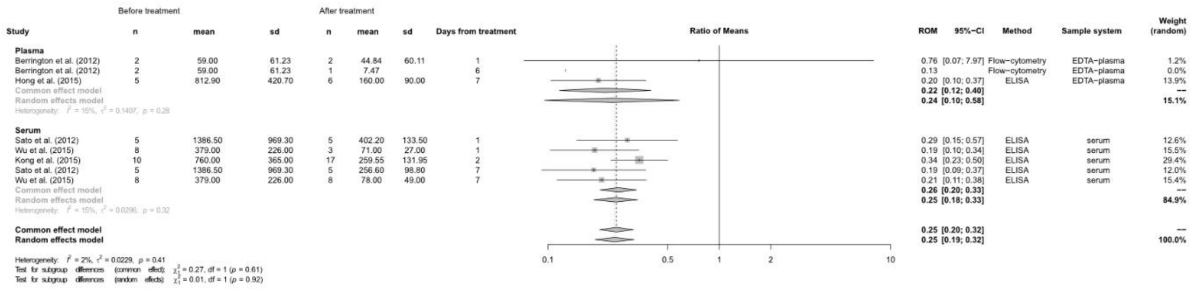

Figure S21 The change of VEGF levels per sample system the first week after treatment with Bevacizumab

Ratio of means meta-analysis performed with meta.cont in RStudio for reported vascular endothelial growth factor (VEGF) concentrations after treatment of retinopathy of prematurity (ROP) with Bevacizumab, ratio of VEGF concentrations calculated against before treatment including a subgroup analysis comparing the sample system (serum vs. plasma).

Abbreviations: SD: standard deviation, CI: confidence interval

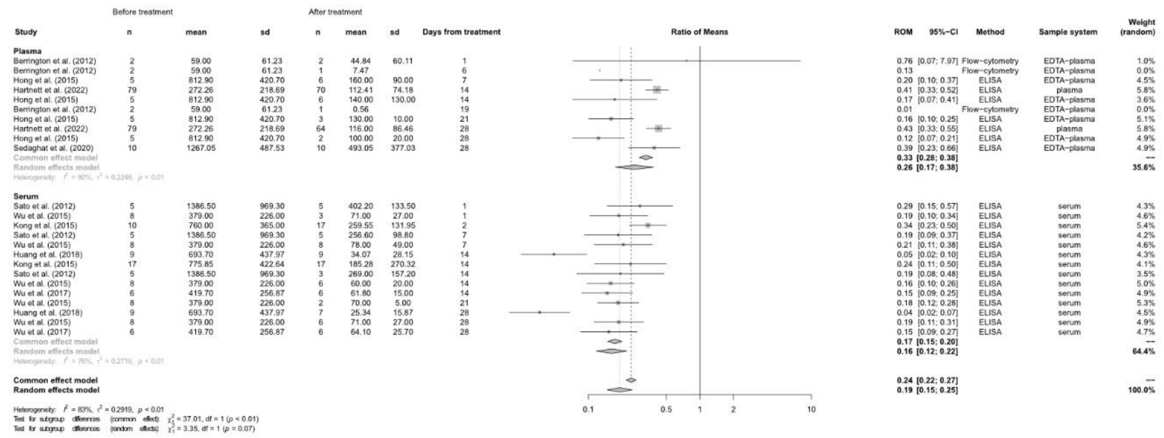

Figure S22 The change of VEGF levels per sample system the first month after treatment with Bevacizumab

Ratio of means meta-analysis performed with meta.cont in RStudio for reported vascular endothelial growth factor (VEGF) concentrations after treatment of retinopathy of prematurity (ROP) with Bevacizumab, ratio of VEGF concentrations calculated against before treatment including a subgroup analysis comparing the sample system (serum vs. plasma).

Abbreviations: SD: standard deviation, CI: confidence interval

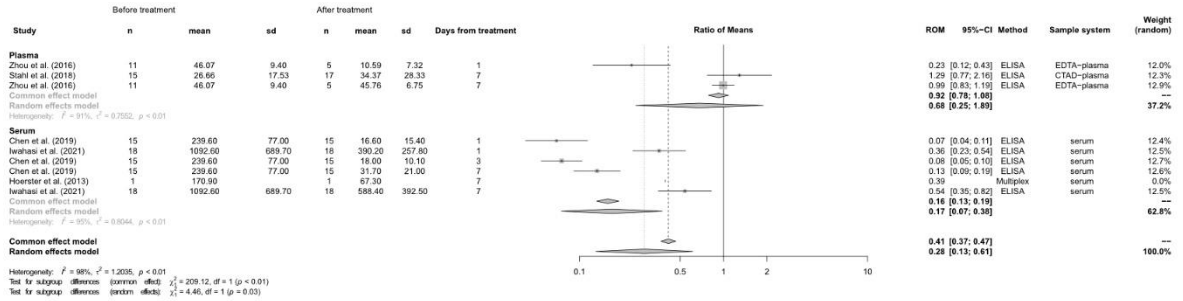

Figure S23 The change of VEGF levels per sample system the first week after treatment with Ranibizumab

Ratio of means meta-analysis performed with meta.cont in RStudio for reported vascular endothelial growth factor (VEGF) concentrations after treatment of retinopathy of prematurity (ROP) with Ranibizumab, ratio of VEGF concentrations calculated against before treatment including a subgroup analysis comparing the sample system (serum vs. plasma).

Abbreviations: SD: standard deviation, CI: confidence interval

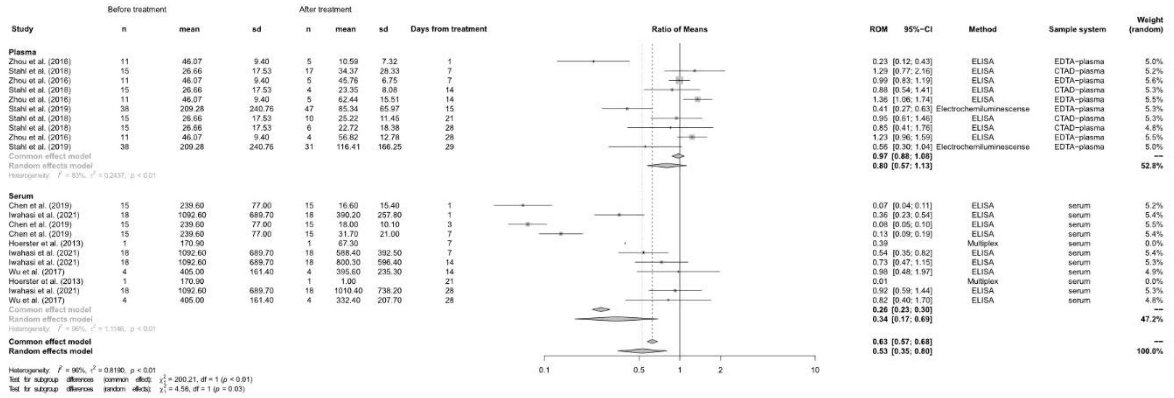

Figure S24 The change of VEGF levels per sample system the first month after treatment with Ranibizumab

Ratio of means meta-analysis performed with meta.cont in RStudio for reported vascular endothelial growth factor (VEGF) concentrations after treatment of retinopathy of prematurity (ROP) with Ranibizumab, ratio of VEGF concentrations calculated against before treatment including a subgroup analysis comparing the sample system (serum vs. plasma).

Abbreviations: SD: standard deviation, CI: confidence interval

Funnel plot- Treatment

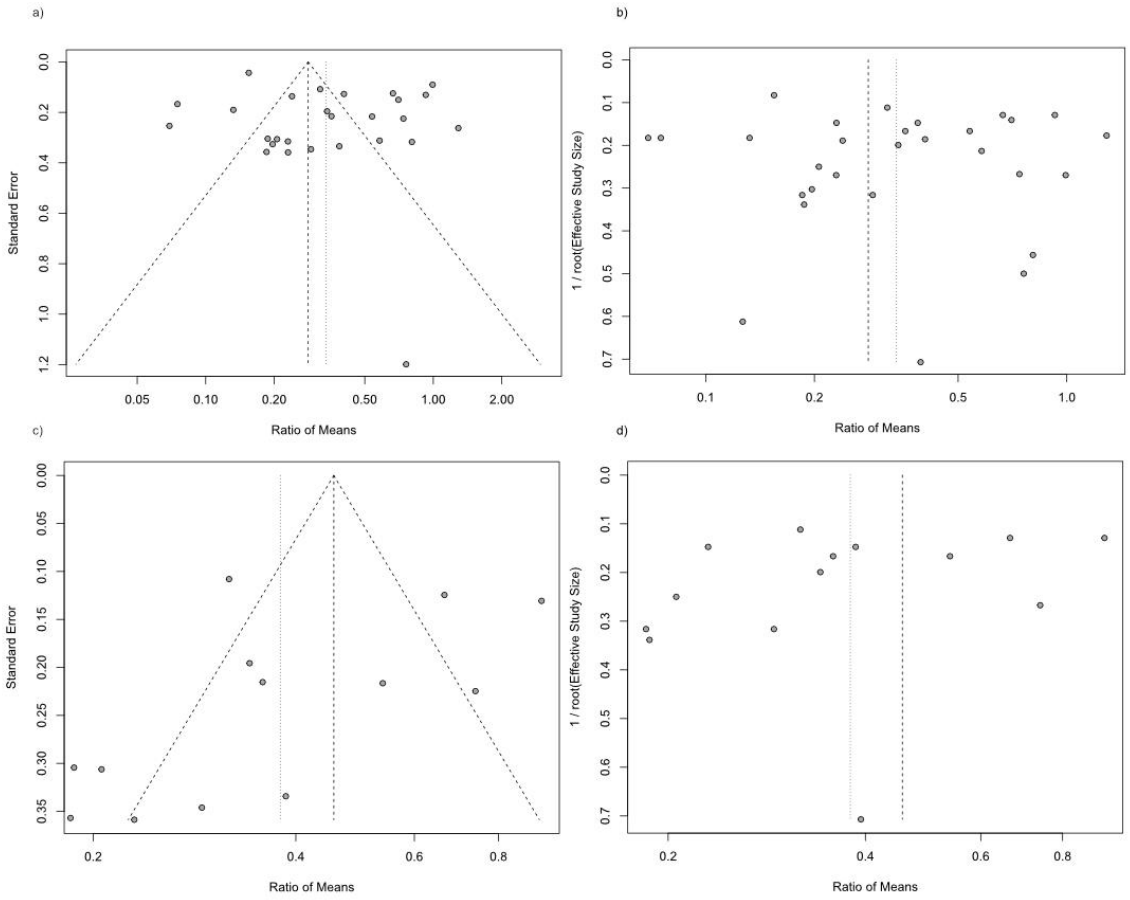

Figure S25 Funnel plots for publications investigating VEGF levels one week after treatment of ROP

Ratio of means calculated against baseline levels before treatment and plotted against

a) against standard error
b) 1/√(effective study size)
c) against standard error excluding publications with a high risk of bias and outliers
d) 1/√(effective study size)

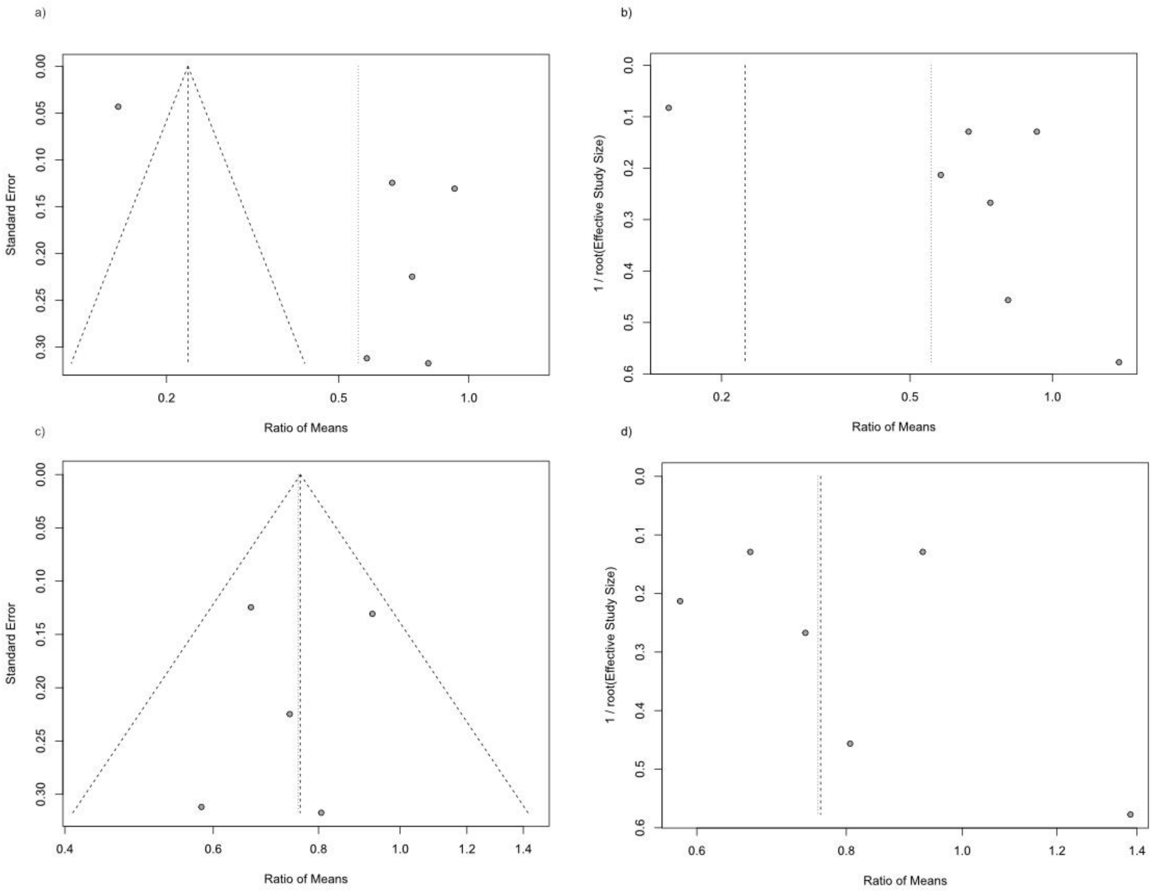

Figure S26 Funnel plots for publications investigating VEGF levels one week after laser treatment of ROP

Ratio of means calculated against baseline levels before treatment and plotted against

a) against standard error
b) 1/√(effective study size)
c) against standard error excluding one outlier
d) 1/√(effective study size) excluding one outlier

a)

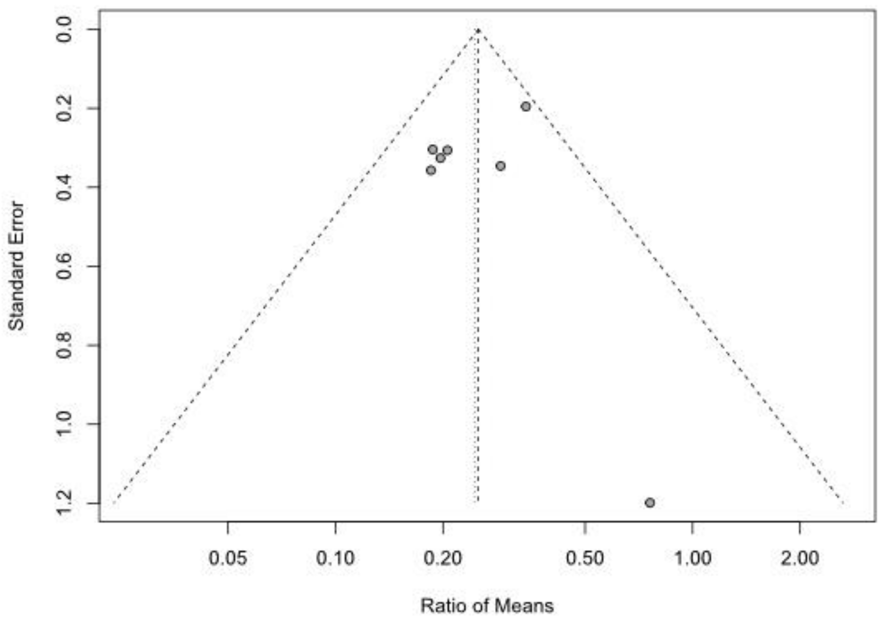

b)

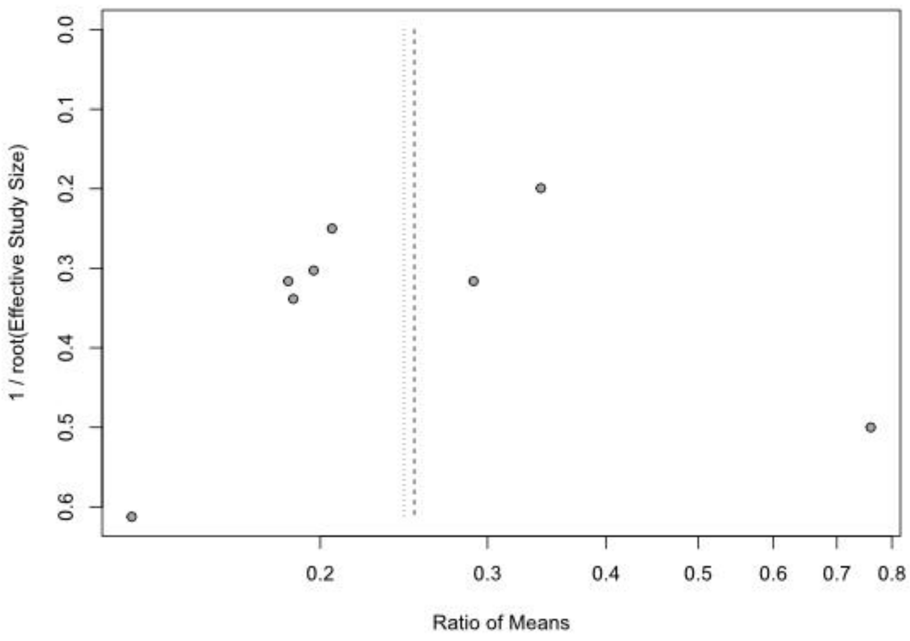

Figure S27 Funnel plots for publications investigating VEGF levels one week after treatment with Bevacizumab for ROP

Ratio of means calculated against baseline levels before treatment and plotted against

a) against standard error
b) 1/√(effective study size)

a)

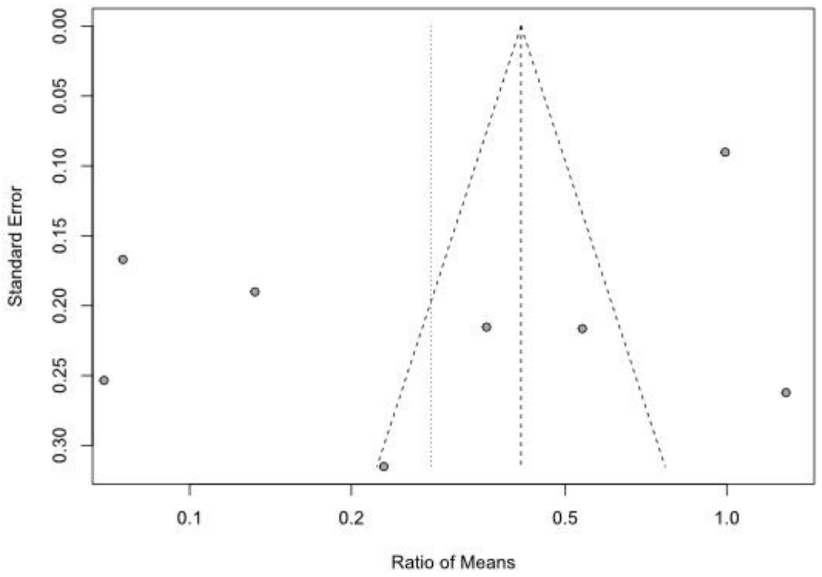

b)

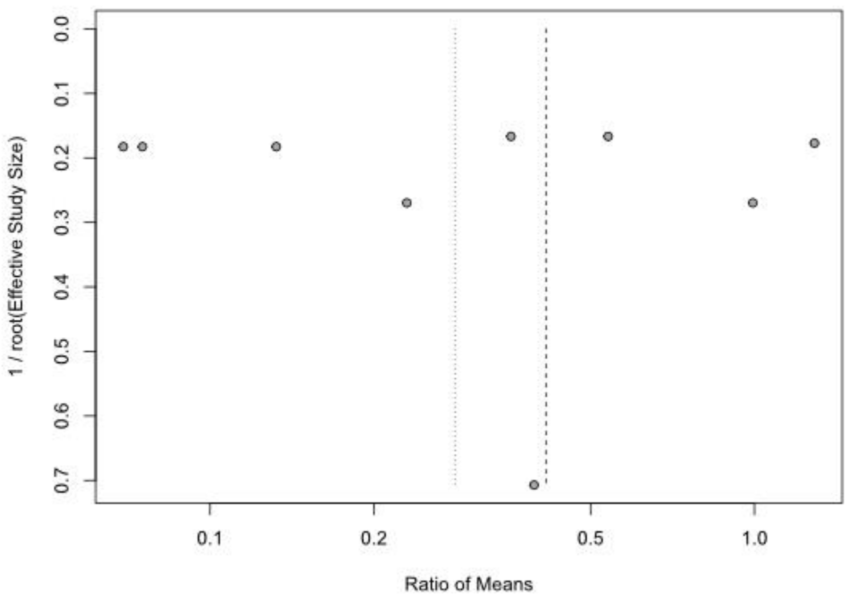

Figure S28 Funnel plots for publications investigating VEGF levels one week after treatment with Ranibizumab for ROP

Ratio of means calculated against baseline levels before treatment and plotted against

a) against standard error
b) 1/√(effective study size)

Subgroup analysis ROP as a biomarker for ROP- PNA and time

a)

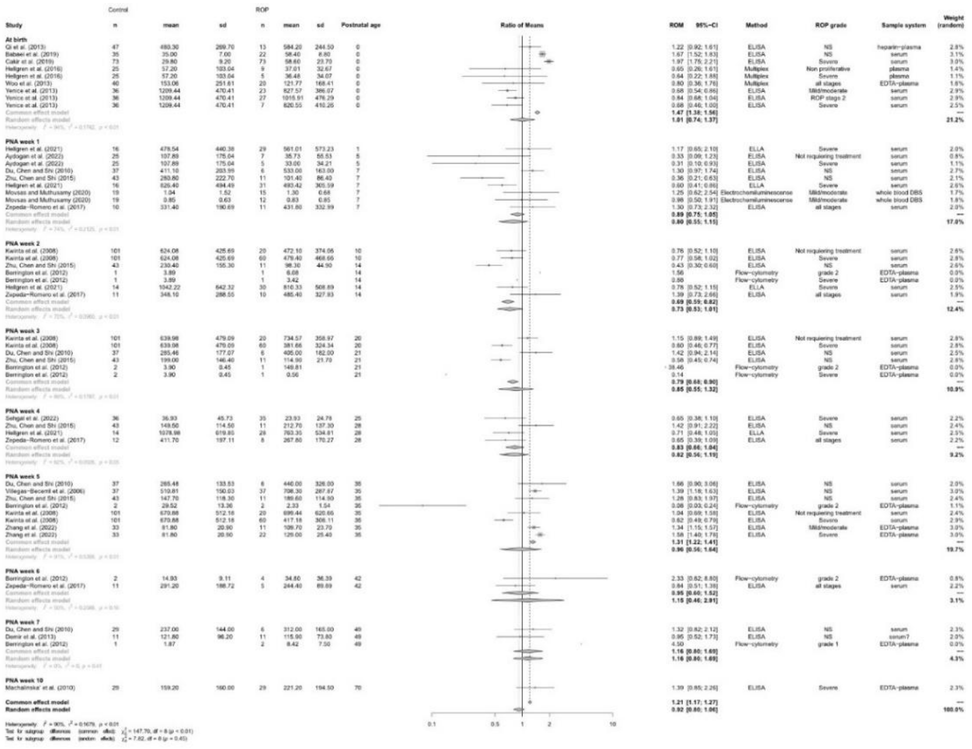

b)

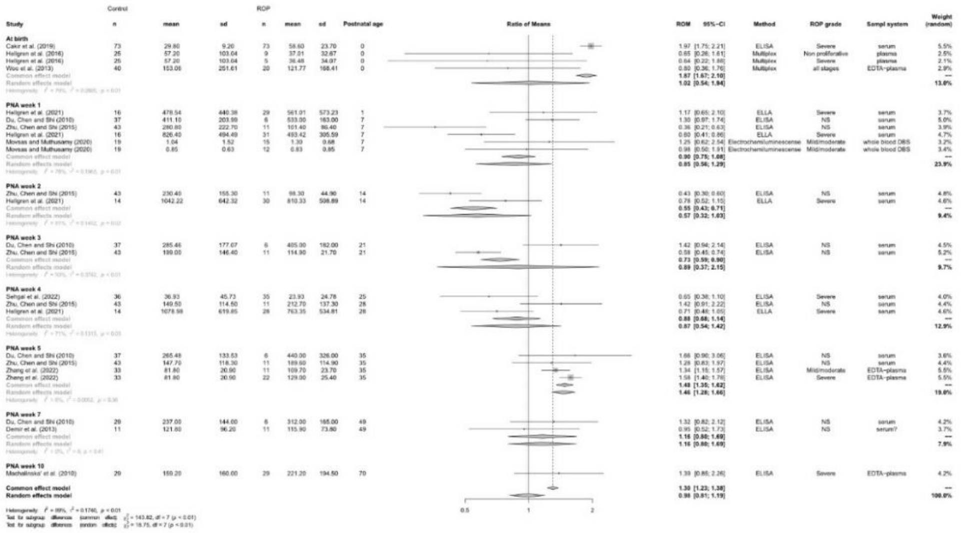

Figure S30 VEGF levels as a biomarker in relation to ROP for postnatal ages

a) Ratio of mean meta-analysis performed with meta.cont in RStudio for reported vascular endothelial growth factor (VEGF) concentrations for infants with ROP compared against a control group, ratio of VEGF concentrations calculated for the ROP group against the control group, including a subgroup analysis of postnatal weeks.
b) Publications with a high risk of bias excluded.

Abbreviations: CI: confidence interval, NS: Not specified, PNA: postnatal age, ROP: retinopathy of prematurity, SD: standard deviation

Subgroup analysis ROP as a biomarker for ROP- PNA, time and ROP severity

a)

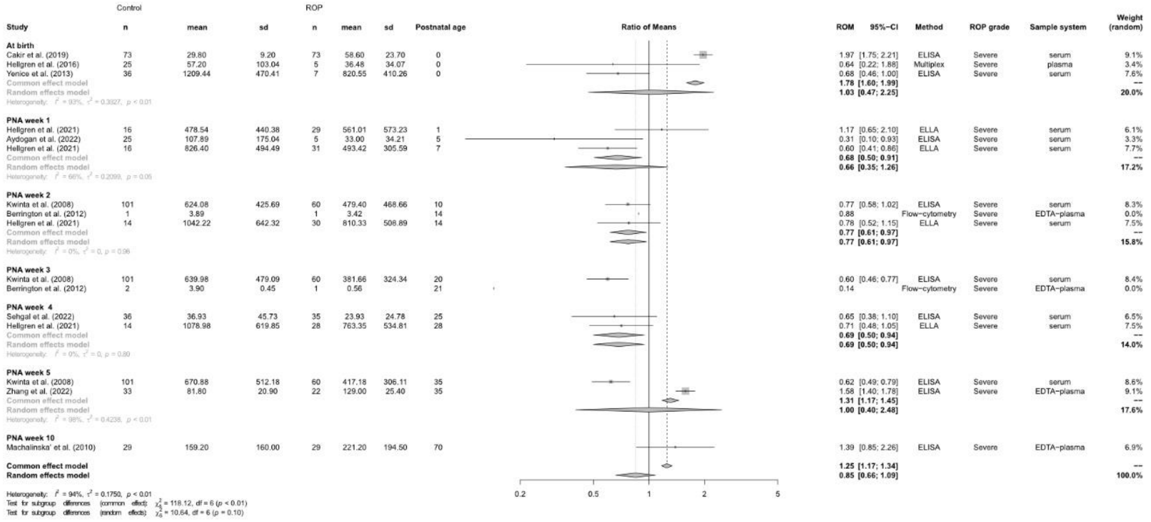

b)

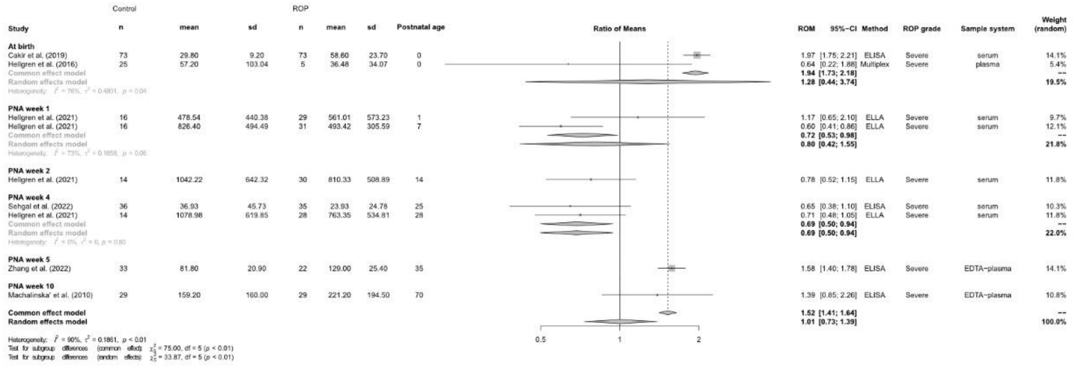

Figure S32 VEGF levels as a biomarker in relation to severe ROP for postnatal ages

a) Ratio of mean meta-analysis performed with meta.cont in RStudio for reported vascular endothelial growth factor (VEGF) concentrations for infants with severe ROP compared against a control group, ratio of VEGF concentrations calculated for the ROP group against the control group, including a subgroup analysis of postnatal weeks.
b) Publications with a high risk of bias excluded.

Abbreviations: CI: confidence interval, NS: Not specified, PNA: postnatal age, ROP: retinopathy of prematurity, SD: standard deviation

Subgroup analysis ROP as a biomarker for ROP- PNA, sample system and time

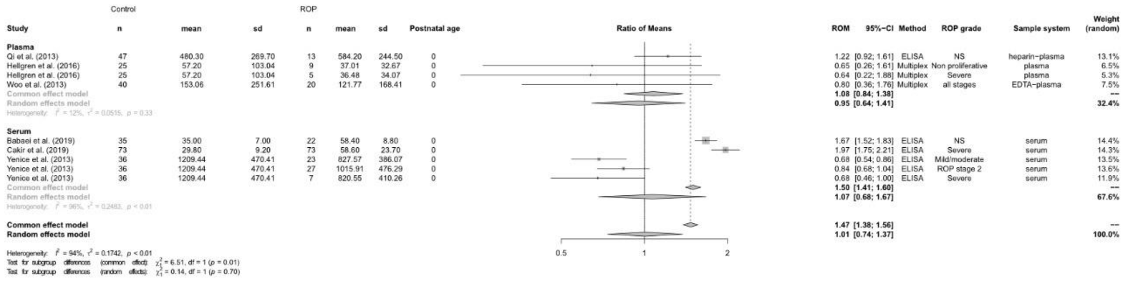

Figure S32 VEGF levels in serum and plasma at birth as biomarker in relation to ROP

Ratio of mean meta-analysis performed with meta.cont in RStudio for reported vascular endothelial growth factor (VEGF) concentrations for infants with ROP compared against a control group during the first day of life, ratio of VEGF concentrations calculated for the ROP group against the control group, including a subgroup analysis of sample system used for the measurement (serum vs. plasma).

Abbreviations: CI: confidence interval, NS: Not specified, ROP: retinopathy of prematurity, SD: standard deviation

Subgroup analysis ROP as a biomarker for ROP- PMA and time

a)

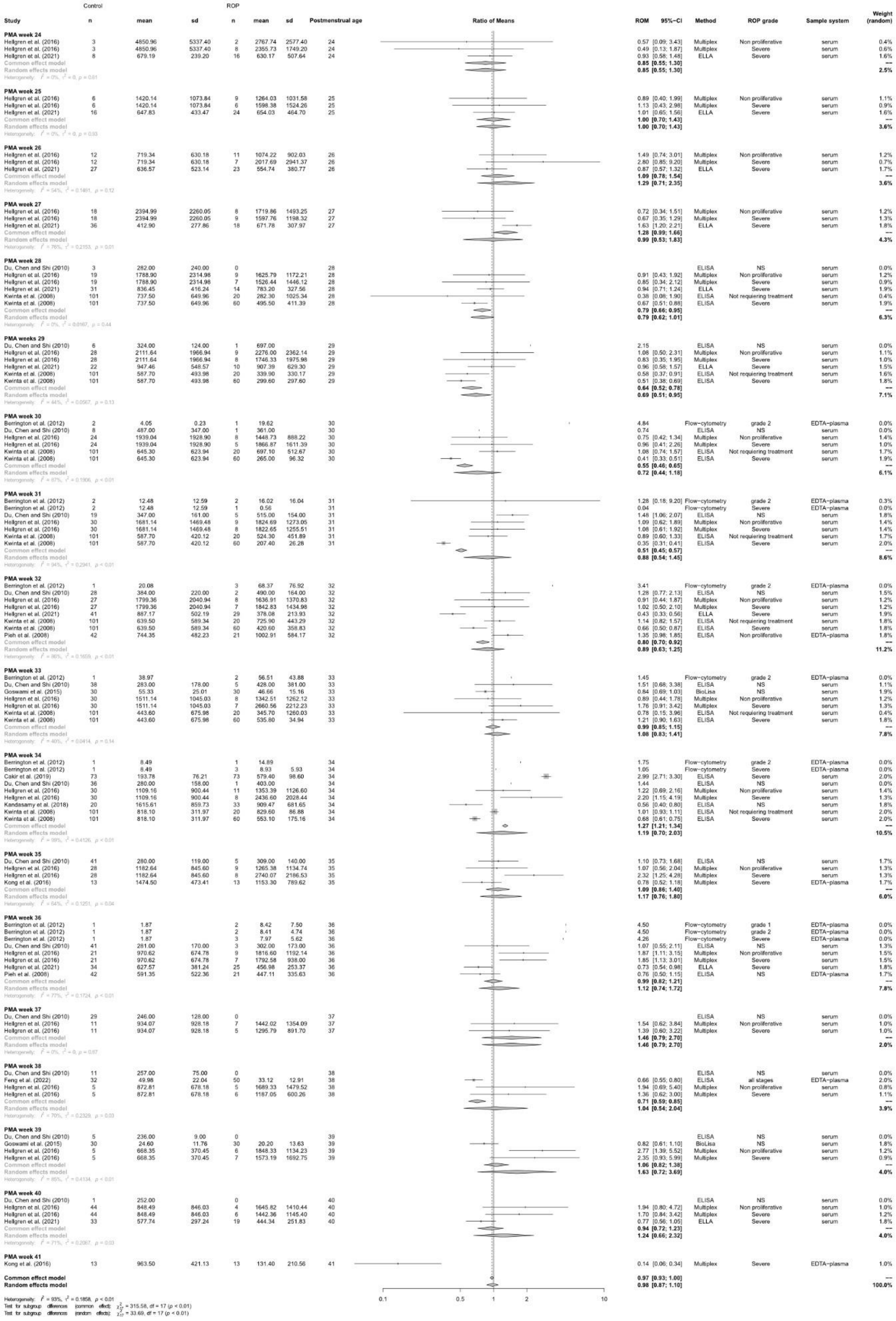

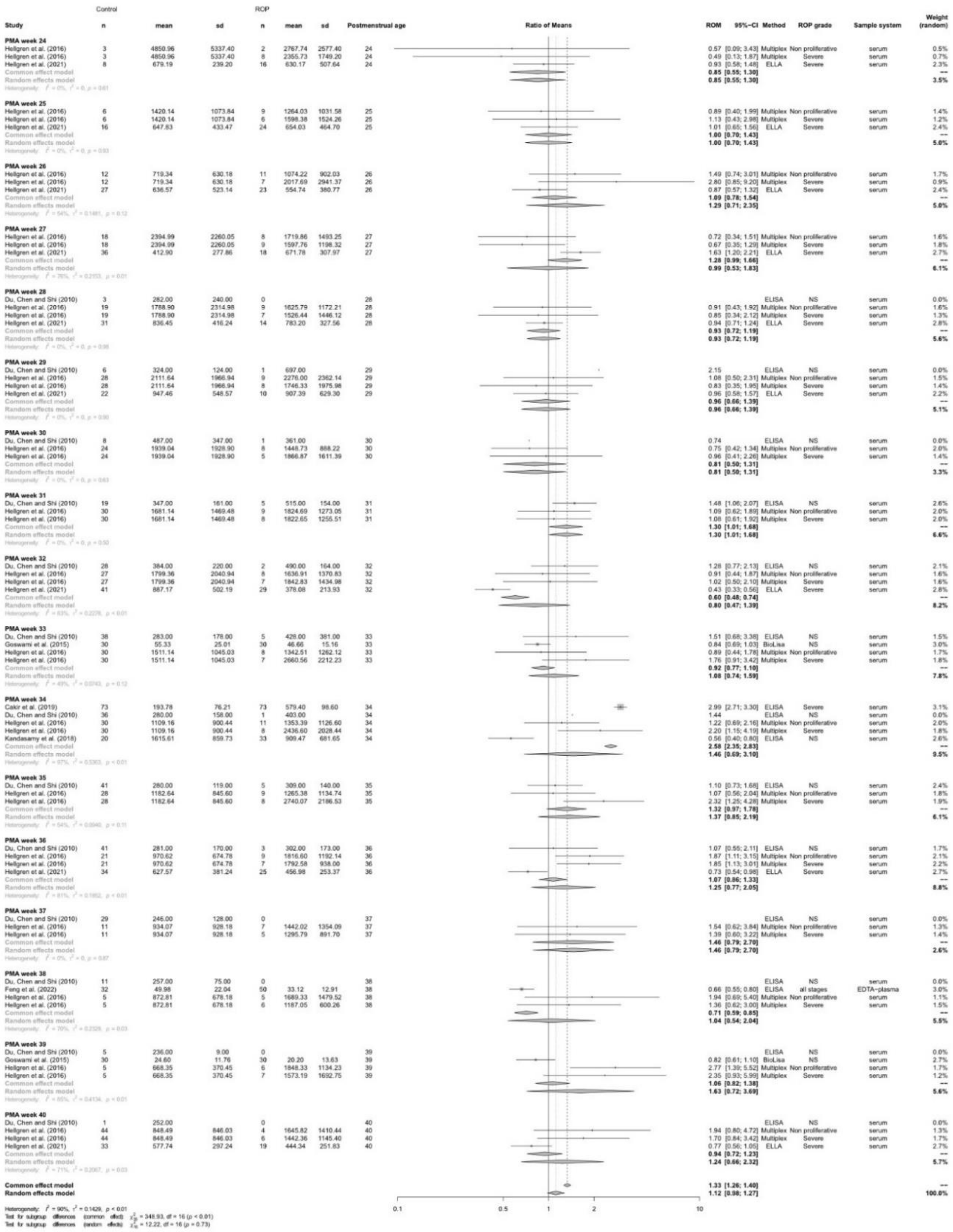

b)

Figure S34 VEGF levels as a biomarker in relation to ROP for postmenstrual ages

a) Ratio of mean meta-analysis performed with meta.cont in RStudio for reported vascular endothelial growth factor (VEGF) concentrations for infants with ROP compared against a control group, ratio of VEGF concentrations calculated for the ROP group against the control group, including a subgroup analysis of postmenstrual weeks.
b) Publications with a high risk of bias excluded.

Abbreviations: CI: confidence interval, NS: Not specified, PMA: postmenstrual age, ROP: retinopathy of prematurity, SD: standard deviation

Subgroup analysis ROP as a biomarker for ROP- PMA, time and ROP severity

a)

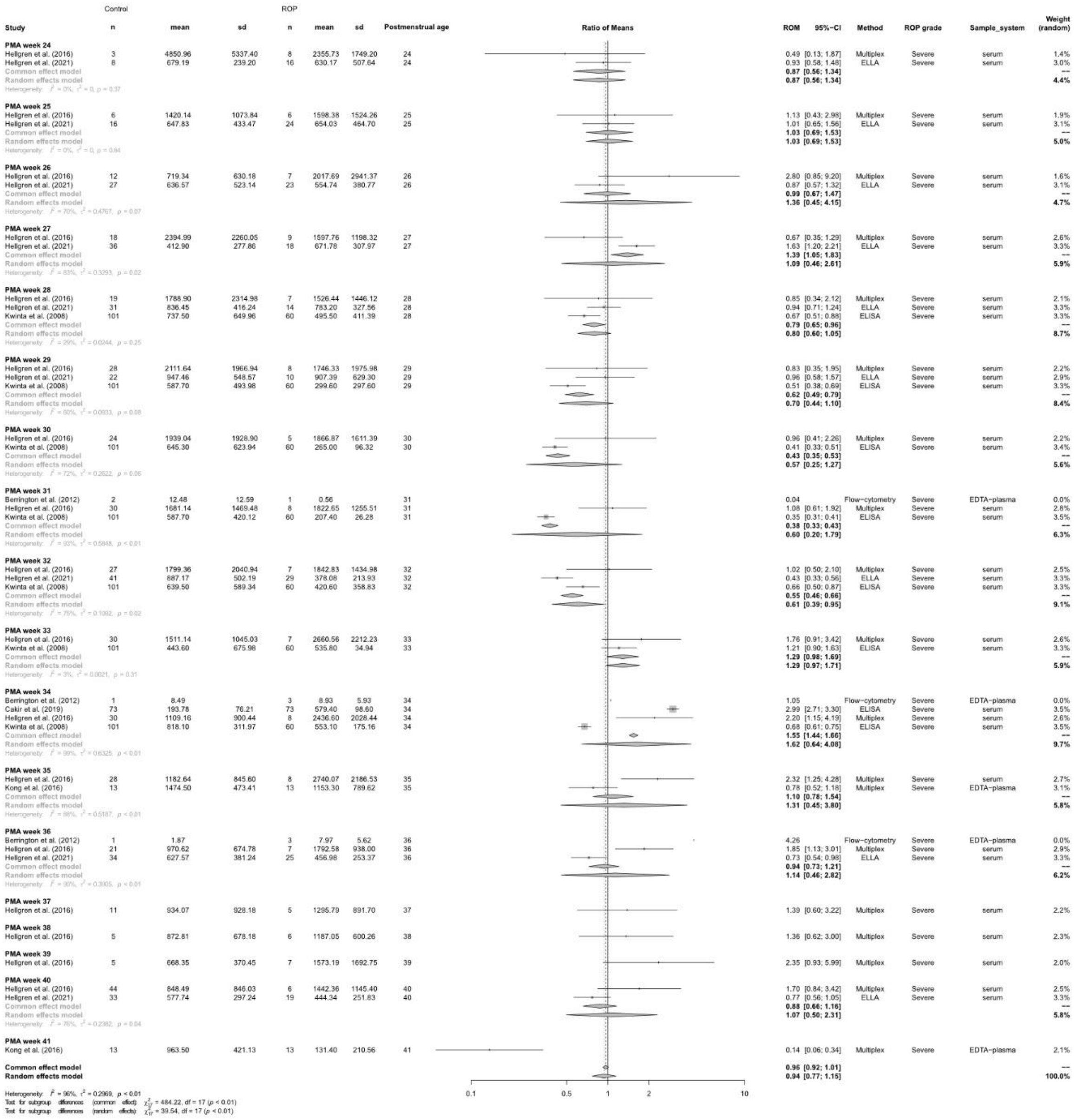

b)

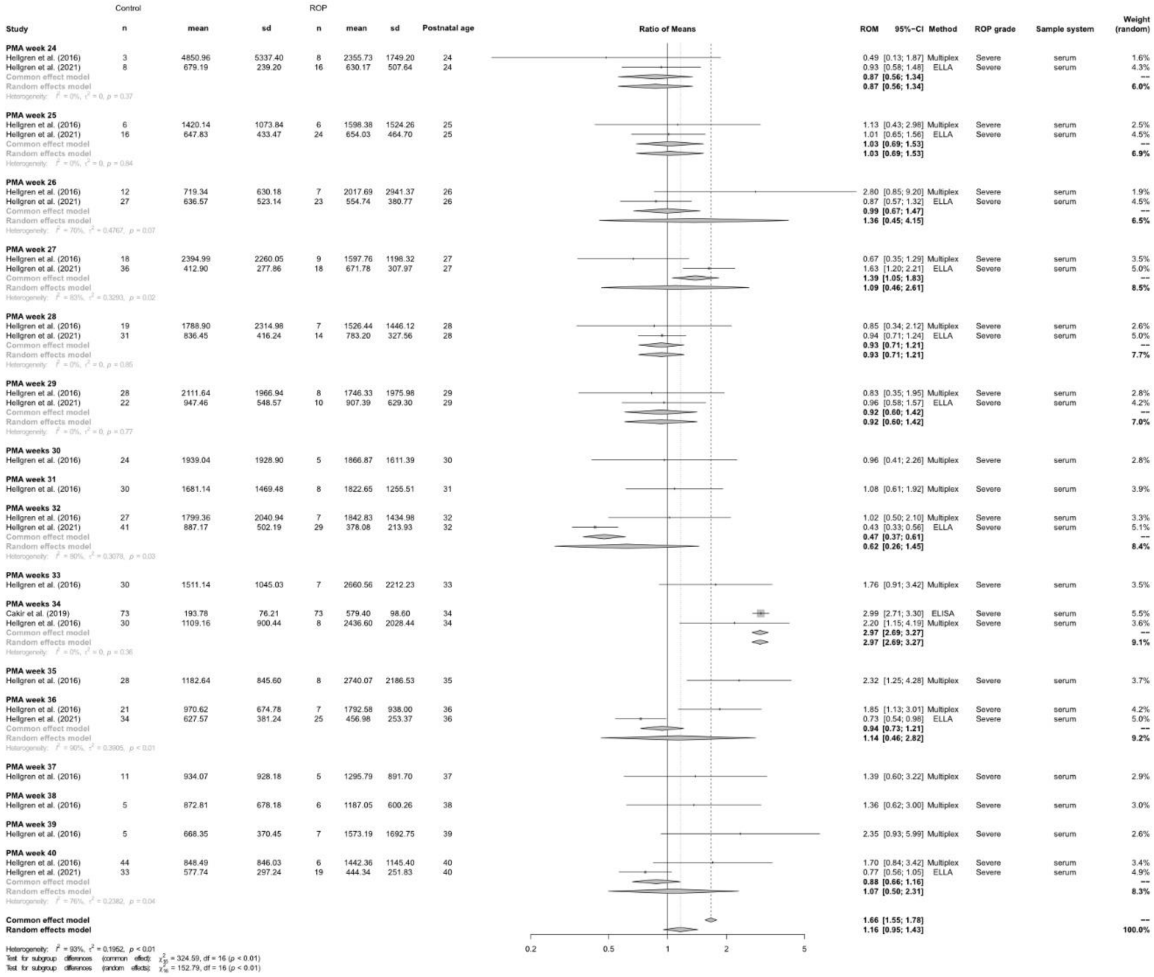

SFigure 35 VEGF levels as a biomarker in relation to severe ROP for postmenstrual ages

a) Ratio of mean meta-analysis performed with meta.cont in RStudio for reported vascular endothelial growth factor (VEGF) concentrations for infants with severe ROP compared against a preterm control group, ratio of VEGF concentrations calculated for the ROP group against the control group, including a subgroup analysis of postmenstrual weeks.
b) Publications with a high risk of bias excluded.

Abbreviations: CI: confidence interval, NS: Not specified, PMA: postmenstrual age, ROP: retinopathy of prematurity, SD: standard deviation

Funnel plot VEGF as a biomarker for ROP

a)

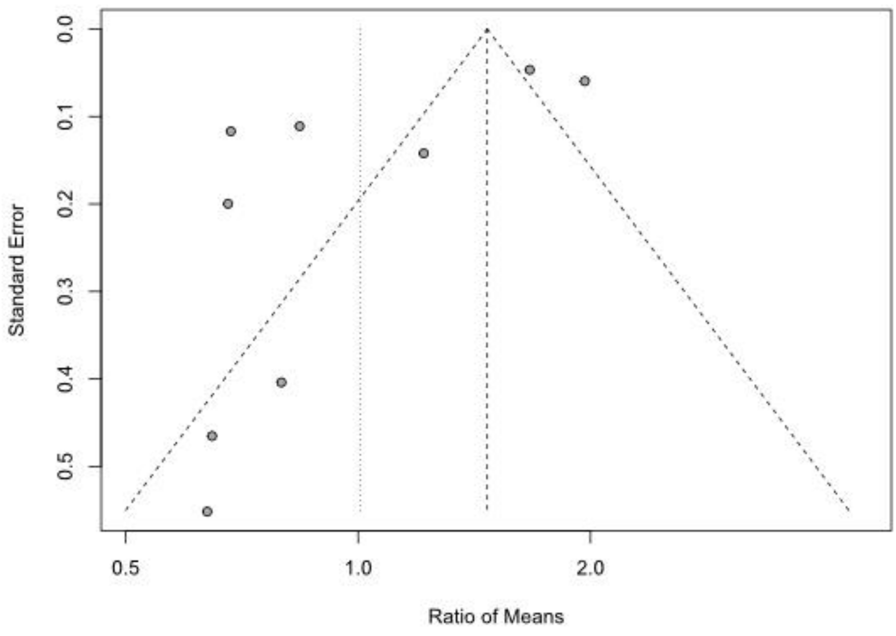

b)

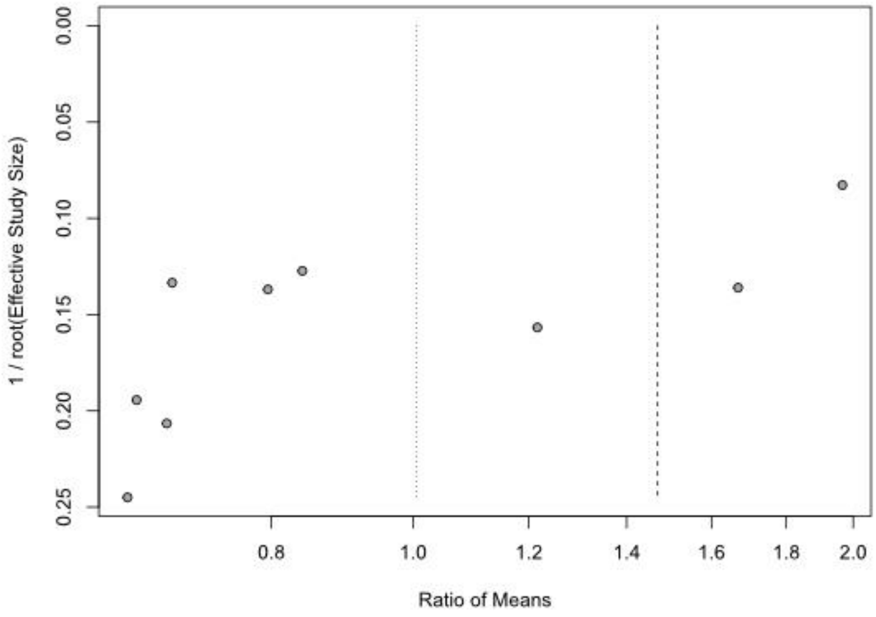

Figure S36 Funnel plots for publications investigating VEGF levels the first postnatal week after birth and comparing levels between a group with ROP and a control.

Ratio of means calculated against the control group and plotted against

a) against standard error
b) 1/√(effective study size)
