# Supplementary material for: Circulating VEGF-A Levels in Relation to Retinopathy of Prematurity and Treatment Effects: A Systematic Review and Meta-Analysis
Source: Ophthalmol Sci. 2024 May 7;4(6):100548. doi: 10.1016/j.xops.2024.100548 (PMC11342886; doi:10.1016/j.xops.2024.100548)
Supplement: Supplement Appendix 2 [file mmc2.pdf]

Supplement Appendix 2

Circulating VEGF-A levels in relation to retinopathy of prematurity and treatment effects: A systematic review and meta-analysis

Short title- VEGF levels in ROP

Précis: Systemic VEGF levels were reduced after treatment of retinopathy of prematurity. Serum shows a more pronounced reduction in VEGF levels than plasma after the intraocular injection of anti-VEGF.

Ulrika Sjöbom[1,2 *], Tove Hellqvist[2], Jhangir Humayun[1], Anders K. Nilsson[2], Hanna Gyllensten[1], Ann Hellström[2], Chatarina Löfqvist[1,2]

Extraction protocol.

- Title
- Publication year
- Study authors
- Study design
- Funding or conflict of interest (fundings reported= F, conflicts reported=C, both F&C)
- Time for study
- Country and place for the study
- Ethics (Following Helsinki declaration, Informed consent (parents), approved by ethical board)
- Aim of the study
- Number of participants
- Descriptions of participants
- Definition of ROP and reason for treatment (if applicable)
- ROP treatment-type (if applicable)
- Results
- Analytical method
- Pre-analytical procedure

VEGF concentrations

- Number of samples
- Time-point for sample
- ROP/ROP grade/No-ROP
- Postmenstrual age at sampling
- Postnatal age at sampling
- VEGF-type measured
- Sample system
- Mean
- Median
- SD
- min
- max
- q1
- q3
- SEM
- Unit

Risk of bias evaluation/QUADAS-2

- Patient selection
    - Describe methods of patient selection- well described?
    - Could the selection of patients have introduced bias? Risk low, high, or unclear
    - Is the description of included patients clear (prior testing, presentation intended use of index test and setting)

  - Is there concern that the included patients do not match the review question? Concern low, high or unclear
- Index test or method used for comparison
  - The specificity of the assay (will be collected from the company that market the assay if not mentioned in the article)
  - How is the experience and the execution of the method used described? (training, established tests, GPC, clinical practice, manufacturer guidance, validation, atypical procedures)
  - How is the performance of the comparison described according to randomisation of samples, singlicate/duplicate measurement etc.
  - Was the uncertainty in the results reported? Results from the reproducibility for the measurements: intra- and or inter assay coefficient of variation
  - Is there concern regarding the performance of the comparison regarding experience, procedure used and uncertainty in the results?
  - How was the comparison performed and described? (each sample according own baseline, groups of samples etc.)
  - Which comparison was performed?
  - Statistic methods, levels of significance
  - Could the conduct or interpretation of comparison have introduced bias? Risk low, high, or unclear
- Reference standard
  - Was the used method/s calibrated against a reference standard?
  - Were the results verified using the same reference standard?
  - Could the reference standard, its conduct, or its interpretation have introduced bias? Risk: low, high, or unclear
  - Is there concern that the target condition (VEGF measurements) as defined by the reference standard does not match the review question? Concern: low, high, or unclear
- Flow and timing
  - Were all patients included in the comparison? (If not, were they described?)
  - Were all samples included in the comparison? (If not, were there any reason?)
  - Is there concern that dropouts have introduced bias? Concern: low, high, or unclear.
  - Were all samples analysed using the same method and reagent batch?
  - Were the samples measured simultaneously? Were all included individuals analysed at the same time?
  - If measurements were separated in time and measurement batches, were the uncertainty from the inter-assay variance used when interpreting the results?
  - Did the whole sample undergo both tests (or one test, if study was randomised)?
  - Was there a difference in the number of uninterpretable or indeterminate results between tests, which is likely to have biased the study results? Reasons for such results could be related to test or patient characteristics (possible bias issue) or could be due to chance, for example, equipment failure, which may be common when using developing technology (although this will reduce study power, it is unlikely to be an important bias issue).
  - Was there an appropriate interval between the index and the comparator tests? Was the same reagent batch used for each of the included tests?
  - Could the flow and timing in the comparison have introduced bias? Risk: low, high, or unclear
