# Supplementary material for: Circulating VEGF-A Levels in Relation to Retinopathy of Prematurity and Treatment Effects: A Systematic Review and Meta-Analysis
Source: Ophthalmol Sci. 2024 May 7;4(6):100548. doi: 10.1016/j.xops.2024.100548 (PMC11342886; doi:10.1016/j.xops.2024.100548)
Supplement: Supplement Appendix 1 [file mmc3.pdf]

Supplement Appendix 1

Circulating VEGF-A levels in relation to retinopathy of prematurity and treatment effects: A systematic review and meta-analysis

Short title- VEGF levels in ROP

Précis: Systemic VEGF levels were reduced after treatment of retinopathy of prematurity. Serum shows a more pronounced reduction in VEGF levels than plasma after the intraocular injection of anti-VEGF.

Ulrika Sjöbom[1,2 *], Tove Hellqvist[2], Jhangir Humayun[1], Anders K. Nilsson[2], Hanna Gyllensten[1], Ann Hellström[2], Chatarina Löfqvist[1,2]

Search strategy

Scopus: TITLE-ABS-KEY ("Vascular Endothelial Growth Factor A" OR vegf OR vegf-a OR "Vascular Endothelial Growth Factor" OR "Vascular Permeability Factor") AND (TITLE-ABS-KEY ("Retinopath*") AND TITLE-ABS-KEY ("Prematur*")) OR (TITLE-ABS-KEY ("Retrolental") AND TITLE-ABS-KEY ("Fibroplas*")) OR (TITLE-ABS-KEY ("Terry") AND TITLE-ABS-KEY ("Syndrom*")) AND TITLE-ABS-KEY (blood OR serum OR plasma OR "whole blood" OR cytokines OR biomarkers OR measurement OR measurements OR "blood specimen" OR "blood samples" OR "blood sample" OR concentration* OR level*)

PubMed: (Vascular Endothelial Growth Factor A[mesh] OR vascular Endothelial Growth Factor A[Title/Abstract] OR VEGF[Title/Abstract] OR VEGF-A[Title/Abstract] OR Vascular Endothelial Growth Factor[Title/Abstract] OR Vascular Permeability Factor[Title/Abstract]) AND (((Retinopathy) AND Prematur*) OR ((Terry) AND Syndrom*) OR ("ROP"[Title/Abstract]) OR "Retinopathy of Prematurity"[Mesh]) AND (blood[Title/Abstract] OR serum[Title/Abstract] OR plasma[Title/Abstract] OR "whole blood"[Title/Abstract] OR "Vascular Endothelial Growth Factor A/blood"[MESH] OR "Retinopathy of Prematurity/blood"[MeSH] OR "Cytokines/blood"[Title/Abstract] OR "Biomarkers/blood"[MeSH] OR measurement[Title/Abstract] OR measurements[Title/Abstract] OR blood specimen[Title/Abstract] OR blood samples[Title/Abstract] OR blood sample[Title/Abstract] OR "Cytokines/blood"[MESH] OR "Neoplasm Proteins/blood"[MESH] OR "Proteoglycans/blood"[MESH] OR blood[MESH] OR concentration*[Title/Abstract] OR level*[Title/Abstract])
